# Setting stakeholder-led research priorities for advancing Sexual and Reproductive Health and Rights in Bangladesh using CHNRI method: an icddr,b initiative

**DOI:** 10.7189/jogh.15.04186

**Published:** 2025-07-11

**Authors:** Abu Sayeed, Nondo Saha, Shafiqul Ameen, Ema Akter, Lubna Hossain, Md. Mehedi Hasan, Fariya Rahman, Sahar Raza, Saraban Tahura Ether, Sabit Saad Shafiq, Hassan Rushekh Mahmood, Ashfia Saberin, Sabina Ashrafee, Husam Md. Shah Alam, Palash Kumar Saha, Sabbir Haider, Supriya Sarkar, Mustufa Mahmud, Md. Jahurul Islam, Shumona Sharmin Salam, Quamrun Nahar, Shams El Arifeen, Anisuddin Ahmed, Ahmed Ehsanur Rahman

**Affiliations:** 1 Maternal and Child Health Division (MCHD), International Centre for Diarrhoeal Disease Research Bangladesh (icddr,b), Dhaka, Bangladesh; 2 National Newborn Health Program & Integrated Management of Childhood Illness (NNHP & IMCI), Directorate General of Health Services, Ministry of Health and Family Welfare, Government of Bangladesh, Dhaka, Bangladesh; 3 Institute of Epidemiology, Disease Control and Research (IEDCR), Directorate General of Health Services, Ministry of Health and Family Welfare, Government of Bangladesh, Dhaka, Bangladesh; 4 Hospital Service Management (HSM), Directorate General of Health Services, Ministry of Health and Family Welfare, Government of Bangladesh, Dhaka, Bangladesh; 5 Maternal, Newborn, Child & Adolescent Health (MNC&AH), Directorate General of Health Services, Ministry of Health and Family Welfare, Government of Bangladesh, Dhaka, Bangladesh; 6 The University of Sheffield, Sheffield, UK; 7 Global Health and Migration Unit, Department of Women’s and Children’s Health, Uppsala University, Uppsala, Sweden

## Abstract

**Background:**

Sexual and reproductive health and rights (SRHR) are essential for individuals' health, well-being, survival, and economic development. A stakeholder-led approach to research prioritisation was essential to guide SRHR-related research in Bangladesh. Accordingly, we conducted a research prioritisation exercise to identify health research priorities related to SRHR in Bangladesh.

**Methods:**

We adopted the Child Health and Nutrition Research Initiative (CHNRI) method for this study. Five themes – adolescent Health (AH), fertility, gynaecological issues (GI), maternal and neonatal health (MNH), and SRH of key populations (SRHKP) – were selected from the broader field of SRHR. Seventy-six experts submitted 454 research questions (RQs), which were then condensed into 197 unique RQs and distributed to all experts for scoring based on five pre-selected criteria. Weighted and unweighted research priority scores (RPS) and average expert agreement (AEA) were calculated to compile a list of top-ranked RQs.

**Results:**

The weighted RPSs for the 197 RQs ranged from 0.944 to 0.623, with a median of 0.848. Among the top 20 list, six RQs belonged to AH, one to Fertility, two to GI, six to MNH, and five to SRHKP. For AH, top ranked RQs included adolescent pregnancy, sexual health education, and mental health. Promoting proper birth spacing among newlywed and underaged married women were top RQs for fertility. GI priorities emphasised early detection of gynaecological cancers, including HPV testing for cervical cancer screening. The MNH research focused on Newborn Stabilizing Units at sub-district hospitals, PPH bundle approaches, and counselling on danger signs to prevent adverse birth outcomes. The top-ranked RQs in SRHKP addressed stigma and discrimination towards key populations (KPs) and their impact on SRH behaviours. There was significant overlap between the top 20 RQs ranked by RPS and AEA.

**Conclusions:**

The study emphasises the need for intervention research to address barriers, assess effectiveness, and enhance the uptake of evidence-based and innovative interventions for SRHR in Bangladesh.

Sexual and reproductive health and rights (SRHR) have been regarded solely as isolated health concerns for a long, without acknowledging their crucial role in individuals' overall health and happiness [1]. Sexual and reproductive health is the state of complete well-being of individuals in relation to their sexuality and reproductive system, encompassing physical, mental, emotional, and social aspects [2,3]. Meanwhile, sexual and reproductive rights aim to safeguard against discrimination, coercion, and violence, while also promoting access to the highest quality of reproductive health care [4,5]. In order to lead healthy and fulfilling lives and realise their complete potential, it is significant to fulfil and uphold the SRHR [1]. However, globally, sexual and reproductive health and rights (SRHR) remain challenged by persistent inequities. Nearly 4.3 billion individuals of reproductive age will experience inadequate access to sexual and reproductive health care [6,7]. Each year, an estimated 25 million unsafe abortions occur [1], nearly 2 million people are newly diagnosed with HIV [8], and one in three women experience intimate partner or sexual violence [6]. Additionally, over 200 million women in low- and middle-income countries express a desire to avoid pregnancy but lack access to modern contraceptive methods [9].

With a population of 173 million, Bangladesh is the world’s 8th most populous country and continues to struggle with significant SRHR challenges despite remarkable advancements [10]. Approximately three out of every five Bangladeshi girls are married before the age of 18, contributing to one of the highest adolescent fertility rates globally [11,12]. Although total fertility has declined over recent decades, the rate has plateaued at 2.3, with a persistent unmet need for effective contraception, particularly long-acting reversible and permanent methods [13,14]. Coverage of essential maternal health services remains low. Only 41% of pregnant women attend four or more antenatal visits, and 21% receive quality ANC [13]. Although Bangladesh has made significant progress in reducing child mortality, improvements in neonatal survival have lagged behind and this accounts for more than 60% of all under-five deaths [15]. Key populations including sex workers and people who inject drugs face disproportionate risks, with HIV prevalence reaching 18.1% in the latter group [16]. These trends underscore critical gaps in SRHR agenda in Bangladesh and the urgent need for locally grounded, evidence-based solutions.

After making significant strides of Millennium Development Goals (MDGs) in advancing SRHR globally, the United Nation Sustainable Development Goals (SDGs) represent a universal, integrated and transformative vision for a sustainable world concerning SRHR [17]. SRHR are critical for sustainable development due to their links to women's well-being, their impact on maternal, newborn, and adolescent health, and their roles in shaping future economic development and environmental sustainability [1]. At the core of the 2030 Agenda is a list of 17 Sustainable Development Goals (SDGs) to end poverty, hunger and inequality; two targets (3.7 and 5.6) were outlined specifically address sexual and reproductive health), ensuring that SRHR remains a priority on the global development agenda [1]. From the 169 targets of SGDs to be achieved by 2030, Bangladesh sets 39 indicators to reach the targets by leaving no one behind in most possible short time under the instructions of SDG Working Committee of The Prime Minister’s Office [18]. Although Bangladesh has already met two SDGs as of 2022, it is still facing major and significant challenges for 14 SDGs related to SRHR [19].

In order to meet the commitment of achieving SDGs related to SRHR by 2030, research prioritisation (RP) is crucial to address critical issues regarding SRHR in Bangladesh, where the current conditions are significantly lacking. RP plays a key role in driving policy and developing evidence-based programmes, which essential for identifying and addressing the most urgent health challenges [20]. By minimising resource waste, RP ensures that attention is focused on urgent needs and sets the agenda for effectively addressing future health concerns. Additionally, RP supports policymakers, programme implementers, and academicians by ensuring that research efforts align with the specific needs of communities and stakeholders. By concentrating on true needs, RP facilitates the development of better policies and programmes that effectively address the most pressing issues [21,22].

The Department of Foreign Affairs, Trade and Development (DFADT) funded the Advancing Sexual and Reproductive Health and Rights (AdSEARCH) project by International Centre for Diarrhoeal Disease Research, Bangladesh (icddr,b) which aimed to improve sexual and reproductive health (SRH) outcomes and realise rights among different population groups with distinct SRH needs in Bangladesh [23]. This multi-year research project would explore, analyse and evaluate some key components of SRHR among high-burden population groups with distinct needs and vulnerabilities through the lenses of gender equality, feminist approach and human rights-based approach. Prioritising research areas are recommended to plan for achieving efficient and impactful investment of limited resources against a large number of competing researches options [24,25]. Failure to align the research agenda with local needs and context may perpetuate disharmony, inequity, and inefficiency in health services, ultimately hindering policy achievement [26,27]. Thus, the utilising a systematic, transparent, objective, and inclusive approach to prioritisation could assist policymakers and research funding agencies in aligning their investment decisions more effectively [26]. Previously, numerous research priorities have been conducted globally on various topics. However, the majority of them have focused on global or regional contexts, with limited attention given to the specific local context of Bangladesh [20]. The needs and challenges faced by Bangladesh may differ significantly from those in global or regional settings, necessitating a more localised approach to research. In the current era, there is a growing emphasis on localisation, which allows for more tailored and context-specific solutions. Although global and regional studies provide valuable insights, they may not adequately capture the specific priorities and challenges unique to Bangladesh. Embracing localisation in research is essential for creating relevant and impactful strategies, ensuring that decision-making processes are led by local expertise and context. This approach is critical to advancing research that aligns with the country’s specific needs and priorities. Another important consideration is that research agendas are typically set by researchers and academics, rather than by policymakers, practitioners, or other stakeholders. As a result, there often exists a gap between programme design and practical implementation. Therefore, to address the gap by engaging a diverse range of stakeholders, including donors, clinicians, policymakers, programme managers, and researchers, the AdSEARCH by icddr,b aimed to identify research priorities related to SRHR issues in Bangladesh using the Child Health and Nutrition Research Initiative (CHNRI) method. CHNRI started as an initiative of the Global Forum for Health Research in Geneva, Switzerland for setting health research priorities [28]. Over the last decade, CHNRI has emerged as a prominent prioritisation methodology, with more than 50 published examples in the literature [20,29]. Here, we present the stakeholder-led research priorities for advancing SRHR in Bangladesh.

## METHODS

### Study design

**Figure 1:**
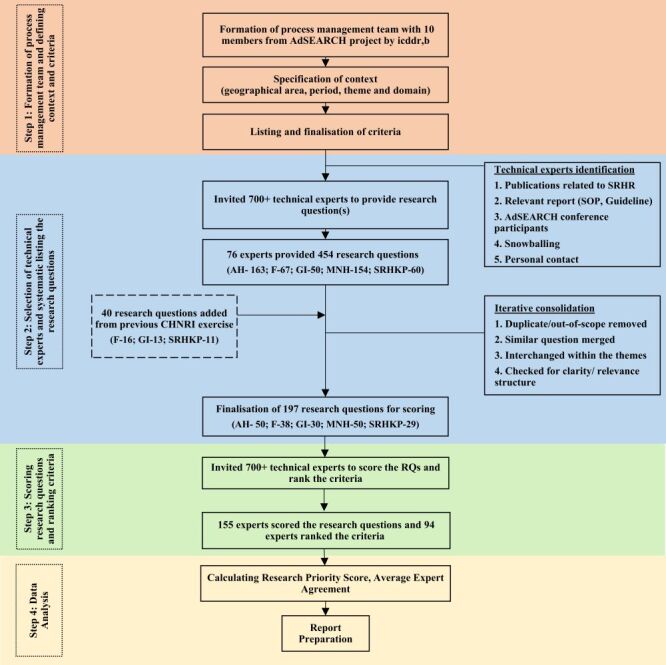
Steps in the Child Health and Nutrition Research Initiative (CHNRI) research priority setting process.

The CHNRI method was adopted to establish research priorities for advancing SRHR in Bangladesh [24]. The process encompassed four key phases aimed at formulating a set of priority research questions, as illustrated in **Figure 1**.

### Step 1: formation of process management team and defining the context and criteria

A process management team consisting of 10 members was identified and established from the AdSEARCH project by icddr,b [23]. The team was responsible for coordinating the research priority-setting exercise, with each member contributing extensive expertise in diverse disciplines such as social science, epidemiology, health systems, innovation, public health research, and statistics. The process management team engaged in detailed discussions to articulate and define the parameters of the research priority exercise. This involved specifying the geographical area, period, research theme, and research domains (**Table 1**). The goal of this study was to identify research questions that could advance the SRHR in Bangladesh and accelerate progress towards achieving SDGs Targets 3, 4, and 5. Therefore, the exercise focused on the national SRHR research agenda and SDG targets to choose the five research themes (Adolescent health (AH), Fertility, Gynaecological issues (GI), and Maternal and neonatal health (MNH), SRH of KP (SRHKP)) (Table S1 in the **Online Supplementary Document**). The CHNRI exercise encompassed research questions from all four broad research domains (4Ds) (**Table 1**).

**Table 1:** Context for the research priority setting exercise

Geographical area	Bangladesh
**Time period**	SDG period (2021–2030)
**Research theme**	AH, fertility, GI, MNH, and SRHKP
**Research domain**	Delivery (research to improve how interventions are delivered, financed, and taken-up), description (research to assess the burden of the problem, its determinants, and effectiveness of interventions to address the problem), development (research to improve interventions that already exist), and discovery (research that leads to innovation *i.e*. entirely new nutrition interventions)

AH – adolescent health, GI – gynaecological issues, MNH – maternal and neonatal health, SDG – sustainable development goals, SRHKP – sexual and reproductive health of key populations

The process management team (PMT) conducted a systematic ranking exercise, followed by an in-depth discussion, to identify four to five key criteria crucial for the priority-setting exercise. To streamline this process, the team thoroughly reviewed all the criteria outlined by Rudan et al. [20] and assigned scores to each criterion based on its perceived relative importance in scoring research questions, utilising a scale ranging from 1 to 10. The top five criteria (Answerability, Deliverability, Effectiveness, Equity, Maximum impact on burden) were meticulously selected for incorporation into the priority-setting exercise from this comprehensive scoring exercise.

### Step 2: selection of technical expert and systematic listing of research questions

We employed both structured and unstructured approaches to identify technical experts in the field of SRHR. This involved conducting a bibliometric search of the PubMed database to identify researchers specialising in SRHR topics such as AH, Fertility, GI, and MNH, SRHKP in Bangladesh, ensuring their contact details were available. We included both national and local researchers with publications relevant to SRHR in Bangladesh. Additionally, we searched government reports, guidelines, and standard operating procedures (SoP) related to SRHR to identify further experts. Furthermore, we enlisted experts from a list of participants who attended a previous AdSEARCH conference on SRHR in Bangladesh. We also encouraged invited experts to distribute the survey links within their networks to enhance participation.

A wide range of technical experts, including researchers, clinicians, policymakers, and programme managers, were invited to participate in the survey. An online platform was developed (Appendix S1 in the **Online Supplementary Document**), and email invitations were distributed to over 700 technical experts to take part in the research priority-setting exercise. In the initial round of the online survey, experts were asked to systematically list research questions on SRHR across the designated themes, with all research methodologies and study designs accepted. Each participant could submit up to five priority research questions per theme based on their expertise. A total of 76 technical experts submitted 454 research questions, averaging 6 questions per person, between January and April 2023.

We incorporated 40 research questions from the prior CHNRI exercise for Fertility, GI, and SRHKP themes, as we received fewer questions from technical experts in these areas. It is notable that the prior CHNRI study also incorporated research questions from previous CHNRI exercise. A subset of the process management team subsequently reviewed and organised the research questions by theme, removing irrelevant questions, categorising and consolidating similar ones, and eliminating duplicates. The resulting list was then shared with the broader process management group, who further refined it to 197 unique questions through collaborative consultation.

### Step 3: scoring of research questions and criteria ranking

The technical experts initially invited to propose research questions were subsequently invited to evaluate the final list of 197 questions through a separate online platform (Appendix S2 in the **Online Supplementary Document**). In the second round of the online survey, experts were requested to evaluate each research question against five criteria: answerability, effectiveness, deliverability, maximum potential for burden reduction, and effect on equity. Scoring options included ‘I agree’ (1 point), ‘I neither agree nor disagree’ (0.5 points), ‘I disagree’ (0 points), and ‘Not well informed’ (blank). To mitigate scoring bias, research questions were randomly presented to experts. Responses were considered valid if experts had scored at least one criterion for each research question. Throughout September to December 2023, a total of 155 technical experts assessed the questions according to five predetermined criteria.

In the second round of the online survey, all experts were requested to rank each predefined criterion based on their perceived relative importance using a five-point Likert scale (1 = most important, 5 = least important). We calculated the observed average rank for each criterion by summing the total ranking scores it received and dividing by the number of responses. These average ranks were then used to determine the weight of each criterion. Ninety-four experts participated in ranking the criteria. The criteria for effectiveness, deliverability and answerability received the highest ranks, followed by max potential for burden reduction, and equity (**Table 2**).

**Table 2:** List of selected criteria used to score research questions

Criteria	Explanation	Observed average rank	Weights
Answerability	The proposed research questions can be ethically answered	2.96	1.01
Deliverability	The interventions resulting from the proposed research question will be affordable, deliverable and sustainable	2.71	1.21
Effectiveness	The proposed research question will be more likely to generate/improve truly effective health interventions	2.49	1.11
Equity	The intervention resulting from the proposed research will be accessible to vulnerable groups thus decreasing inequity	3.53	0.91
Maximum impact on burden	The research question has greater potential to reduce disease burden	3.31	0.85

### Step 4: data analysis

We utilised a password-protected Microsoft Structured Query Language (SQL) Server 2008 R2 as the central database to ensure the data security and quality. To minimise inconsistencies and errors during data entry, we frequently implemented validation rules, including consistency checks, logical checks, and skip patterns.

We computed intermediate, unweighted, and weighted research priority scores (RPS) for each of the five scoring criteria utilised to differentiate among the 197 research questions in this exercise [24]. Experts provided responses for each research question, with 1 indicating ‘I agree’, 0 indicating ‘I disagree’, 0.5 ‘I neither agree nor disagree’, and blank indicating insufficient information to assess a research question. The intermediate RPS was calculated by summing all informed responses (1, 0, or 0.5) and dividing this sum by the total number of informed responses, excluding blanks from both the numerator and denominator. The intermediate RPS, ranging from 0 to 1, represented the collective optimism of both scorers and informed experts regarding the likelihood that a research question would fulfil a specific criterion. This approach to handling missing answers/blanks enhances prediction accuracy by enabling experts who may not possess sufficient knowledge to score a research question adequately against each criterion to abstain from answering [30,31]. The unweighted RPS was calculated as the mean of all five intermediate priority scores.

In the subsequent stage, we determined weights by dividing the observed average rank for each criterion by the expected average rank, where all five criteria are equally valued (which would be 3.00) [32]. In our analysis, for every scored research question, the intermediate score for effectiveness and deliverability criteria were augmented by 21 and 11%, whereas there was minimal alteration (1%) in score for the answerability criterion. Conversely, the score decreased by 15 and 9% for equity and max potential for burden reduction criteria, respectively (**Table 2**). We then multiplied these weights with the intermediate scores of each criterion to compute weighted intermediate scores and subsequently calculated the weighted RPS as the mean of all the weighted intermediate scores for each RQ [33].



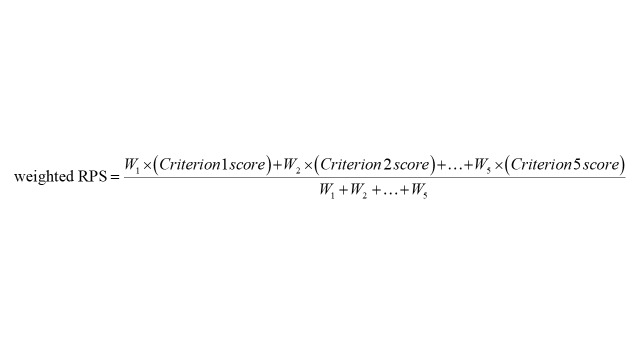



Additionally, we computed the average expert agreement (AEA) for each of the 197 research questions using the following formula [24]. The AEA provided insight into the proportion of scorers who provided the same most frequent response from the four responses [34,35]. It serves as a measure of concurrence or disagreement among scorers' opinions regarding the RPS and remains unaffected by the differing number and composition of scorers per criterion [36].



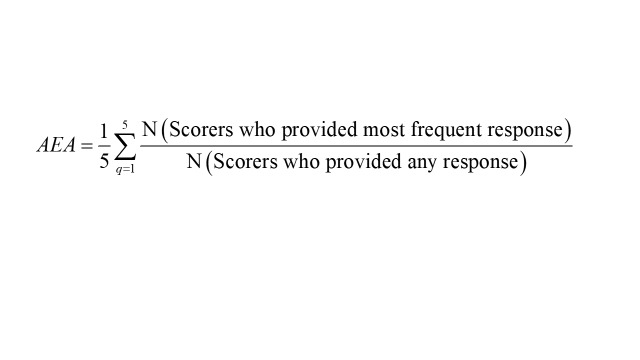



## RESULTS

### Characteristics of technical experts

Demographic characteristics of the experts participated in this study are presented in the **Table 3**. Experts with age group of 31–40 years were highest responders, accounting for 25 respondents (42.4%) in Phase 1 and 50 respondents (32.3%) in Phase 2. Males were prominent among all the experts in both phase (56.6 and 53.6% respectively). Most respondents (84.9 and 79.4%) in both phases reported their primary affiliation as being a researcher. Regarding academic qualification, more than 3 out of 5 experts reported the completion of master/equivalent level in Phase 1 (61.1%) and Phase 2 (65.8%). Experts were experienced on overage of 14.2 years in Phase 1 and 14.1 years in Phase 2. The MNH was the most reported area of expertise in both phase (74.3 and 71.6% respectively), where AH was the second dominant area of expertise in phase 1 (51.4%) and SRHKP in Phase 2 (49.0%).

**Table 3:** Sociodemographic characteristics of the participants (n = 231)

Variables	Phase 1:	Phase 2:
**listing research questions (n = 76)**	**scoring research questions (n = 155)**
**Age (mean ± SD)**	40.75 ± 10.23	41.73 ± 12.39
**Age category**		
Less than 30 y	10 (16.95)	37 (23.87)
31–40 y	25 (42.37)	50 (32.26)
41–50 y	12 (20.34)	34 (21.94)
More than 50 y	12 (20.34)	34 (21.94)
**Gender**		
Female	33 (43.42)	72 (46.45)
Male	43 (56.58)	83 (53.55)
Role of the participants*		
Researcher	62 (84.93)	123 (79.35)
Academic	18 (24.66)	38 (24.52)
Funder/doner	1 (1.37)	7 (4.52)
Clinician	9 (12.33)	16 (10.32)
Policy maker	1 (1.37)	10 (6.45)
Programme manager	12 (16.44)	11 (7.10)
Others**†**	6 (8.22)	21 (23.55)
**Academic qualification**		
Bachelor degree/MBBS/equivalent	6 (8.33)	18 (11.61)
Master of Science (MSc)/MPH/equivalent	44 (61.11)	102 (65.81)
PhD or higher	22 (30.56)	35 (22.58)
**Years of experience (mean ± SD)**	14.16 ± 10.52	14.12 ± 10.88
**Years of experience category**		
Less than 10 y	35 (52.24)	74 (48.37)
11–20 y	15 (22.39)	44 (28.76)
21–30 y	13 (19.40)	22 (14.38)
More than 30 y	4 (5.97)	13 (8.50)
Area of expertise *		
Adolescent health	38 (51.35)	72 (46.45)
Fertility	10 (13.51)	29 (18.71)
Maternal and neonatal health	55 (74.32)	111 (71.61)
Gynaecological issues	7 (9.46)	20 (12.90)
Sexual and reproductive health of KP	21 (28.38)	76 (49.03)
Theme-wise participant count*		
Adolescent health	50 (65.79)	68 (43.87)
Fertility	18 (23.68)	69 (44.52)
Maternal and neonatal health	48 (63.16)	70 (45.16)
Gynaecological issues	15 (19.74)	59 (38.06)
Sexual and reproductive health of KP	21 (27.63)	67 (43.23)

KP – key populations, MBBS – Bachelor of Medicine, Bachelor of Surgery, MPH – Master of Public Health, SD – standard deviation

*Multiple answer applicable.

**†**Stand for public health professionals, advisor, non-governmental organisation professional, activist and statistician.

### Research priorities for Bangladesh on SRHR

**Figure 2:**
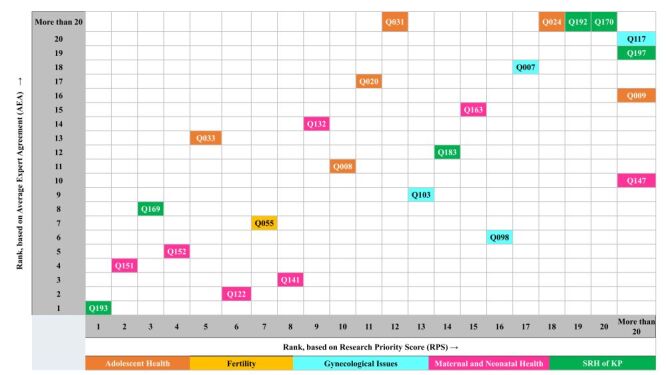
Top 20 research questions based on cross-cutting value of research priority scores (RPS) and average expert agreement (AEA).

The overall weighted RPSs for the 197 RQs ranged from 0.944 (highest) to 0.623 (lowest), with a median score of 0.848. The full set of 197 research questions with theme, domain, criteria-wise intermediate score, unweighted RPS, weighted RPS, and AEA can be found in the data set available in Table S2 in the **Online Supplementary Document****.** **Table 4** represents the top 20 RQs ranked by the weighted RPS. Among the top 20 list, six RQs were from AH theme (Q5, Q10, Q11, Q12, Q17, and Q18), one was from fertility theme (Q7), two were for GI theme (Q13 and Q16), six were from MNH theme (Q2, Q4, Q6, Q8, Q9, and Q15), and the remaining five RQs were from the theme of SRHKP (Q1, Q3, Q14, Q19, and Q20). Half of the top 20 RQs were from description domain. Seven (35%) and three (15%) of the top 20 RQs were from delivery and discovery domain. No RQ of the top 20 RQs were from development domain. **Figure 2** denotes the top ranked RQ based on the value of weighted RPS and AEA. There was considerable overlap between top 20 RQs based on weighted RPS and top 20 RQs based on AEA. Analysis showed that among the top 20 RQs ranked by weighted RPS, 16 RQs were among the top 20 RQs ranked by weighted AEA.

**Table 4:** Top 20 research questions with domain, theme, scores for each criterion, weighted research priority score (RPS), and average expert agreement (AEA)

Question No.	Rank	Research question	Domain	Theme	Answerability intermediate RPS	Deliverability intermediate RPS	Effectiveness intermediate RPS	Equity intermediate RPS	Maximum impact on burden intermediate RPS	Weighted RPS	AEA
Q193	1	How can stigma and discrimination against Key Populations be reduced to improve their sexual and reproductive health?	Discovery	SRHKP	0.962	0.913	0.971	0.913	0.962	0.945	0.906
Q151	2	What barriers hinder implementing Newborn Stabilizing Units in upazila hospitals?	Delivery	MNH	0.981	0.894	0.940	0.922	0.922	0.932	0.891
Q169	3	What are the obstacles in raising awareness about the Sexual and Reproductive Health Rights (SRHR) of Key Populations (KPs) in Bangladesh?	Delivery	SRHKP	0.982	0.891	0.909	0.891	0.963	0.926	0.882
Q152	4	What is the impact of the Postpartum Haemorrhage (PPH) bundle approach on the management of PPH cases?	Description	MNH	0.971	0.910	0.912	0.890	0.942	0.925	0.890
Q033	5	How is adolescent pregnancy associated with child undernutrition, maternal anemia, and the risk of NCDs such as diabetes, and hypertension of the mother?	Description	AH	0.989	0.907	0.932	0.807	0.977	0.925	0.869
Q122	6	Does the enhancement of counselling on danger signs during antenatal care (ANC) contribute to a significant reduction in adverse birth outcomes?	Description	MNH	0.952	0.912	0.923	0.902	0.933	0.925	0.895
Q055	7	What key factors hinder or support birth spacing strategies for newlywed and underage married women, and how can they be integrated into health care systems?	Delivery	F	0.958	0.872	0.957	0.926	0.904	0.924	0.885
Q141	8	What is the burden and risk factors for mental health issues among pregnant women?	Description	MNH	0.980	0.875	0.929	0.875	0.958	0.924	0.893
Q132	9	What are the bottlenecks, barriers, and challenges in identifying and managing high-risk pregnancies in low- and middle-income countries like Bangladesh?	Delivery	MNH	0.971	0.882	0.933	0.880	0.942	0.922	0.868
Q008	10	What is the impact of school-based comprehensive sexuality education given to young adolescents on prevention of gender-based violence, unsafe abortion, and smooth transitioning to adolescence from childhood?	Description	AH	0.946	0.886	0.933	0.889	0.943	0.920	0.872
Q020	11	What are the barriers faced by health care provider to provide mental health services among adolescents?	Delivery	AH	0.951	0.898	0.931	0.885	0.922	0.919	0.861
Q031	12	What is the impact of adolescent pregnancy on maternal mortality/morbidity?	Description	AH	0.958	0.896	0.927	0.894	0.904	0.917	0.854
Q103	13	What are the challenges, key factors, and enablers of implementing HPV testing for cervical cancer screening?	Delivery	GI	0.967	0.864	0.944	0.884	0.909	0.915	0.878
Q183	14	What strategies need to be implemented to get better sexual and reproductive health & family planning service from government health system by key populations (KP)?	Discovery	SRHKP	0.931	0.888	0.923	0.865	0.962	0.914	0.870
Q163	15	What are the most effective interventions for the prevention and treatment of low birth weight infants?	Description	MNH	0.950	0.880	0.929	0.878	0.920	0.912	0.867
Q098	16	What strategies can be implemented to scaling up the early detection of gynecological cancers in Bangladesh?	Discovery	GI	0.946	0.898	0.933	0.860	0.911	0.912	0.888
Q007	17	What is the impact of introducing life skills lessons in the school educational curriculum on empowerment of adolescent girls and prevention of child marriage?	Description	AH	0.978	0.889	0.913	0.848	0.911	0.910	0.861
Q024	18	What is the impact of health education on the preference of using menstrual kit among adolescent girl?	Description	AH	0.979	0.904	0.948	0.819	0.862	0.908	0.841
Q192	19	How do stigma and discriminatory practices impact the sexual and reproductive health behaviors of Key Populations?	Description	SRHKP	0.958	0.880	0.880	0.880	0.930	0.905	0.842
Q170	20	What challenges do key populations faced during COVID-19 testing, treatment when COVID-19 positive, and the administration of vaccination against COVID-19?	Delivery	SRHKP	0.969	0.833	0.930	0.896	0.890	0.904	0.848

AH – adolescent health, F – fertility, GI – gynaecological issues, MNH – maternal and neonatal health, SRHKP – sexual and reproductive health of key populations, NCDs – non-communicable diseases, HPV – human papillomavirus

The RQs from AH theme among top 20 RQs were focused on exploring the effect of adolescent pregnancy on stunting, anaemia, other non-communicable chronic diseases and maternal mortality (Q5 and Q12), comprehensive sexual and health education and life skill lessons for adolescent (Q10, Q17, and Q18), barriers for providing mental health services (Q11). The only RQ from fertility theme among top 20 RQs were focused on promoting proper birth spacing among newlywed and underage married women (Q7). RQs from GI theme among top 20 RQs were focused on early detection of gynaecological cancers including human papillomavirus (HPV) testing for cervical cancer screening (Q13 and Q16). RQs from MNH theme among top 20 RQs were focused on implementation of Newborn Stabilizing Unit (NSU) at sub-district level hospital (Q2), Postpartum Haemorrhage (PPH) bundle approach (Q4), counselling on danger signs during antenatal care (ANC) (Q6), burden of mental health among pregnant women (Q8), identifying and managing high-risk pregnancies (Q9), prevention and treatment of low birthweight infants (Q15). The top-ranked RQ among top 20 RQs was identifying strategies to address stigma and discriminatory practices towards KPs and mitigate their negative effects on the SRH behaviours of KPs (Q1), which was from SRHKP theme. Rest five RQs from this theme among top 20 RQs were focused on obstacles in raising awareness about SRHR among KPs in Bangladesh (Q3), receiving sexual and reproductive health & family planning service from government health system by KPs (Q14), impacts of stigma and discriminatory practices on SRH behaviours of KPs (Q19) and challenges faced by KPs during regarding COVID-19 testing, treatment and vaccination (Q20).

### Theme wise research priorities for Bangladesh

We conducted theme wise analyses for identifying top 10 RQs for the five pre-defined research themes.

#### AH theme

Among the 50 RQs related to adolescent health, the highest weighted RPS was 0.940 and the lowest was 0.744. **Table 5** denotes the top 10 prioritised questions within AH theme. Four RQs were entered into the top 10 RQs of AH theme, apart from the six previously selected RQs into the overall top 20 RQs. The newly selected four RQs from the top 10 RQs within the AH theme were focused on effects of unwanted pregnancy on peripartum depression (Q25), knowledge and decision-making skills of adolescent (Q27), educating young adolescents about adverse effects of self-termination of pregnancy’s using traditional medicine (Q31) and adolescent’s well-being and mental health (Q36). Eight (80%) of the top 10 research priorities were categorised into description domain or epidemiological research, one as research to develop existing interventions (10%), and one as research to scale-up an intervention.

**Table 5:** Top 10 research questions by Adolescent Health theme with domain, scores for each criterion, weighted research priority score (RPS), and average expert agreement (AEA)

Question No.	Overall Rank	Theme wise Rank	Research Question	Domain	Answerability intermediate RPS	Deliverability intermediate RPS	Effectiveness intermediate RPS	Equity intermediate RPS	Maximum impact on burden intermediate RPS	Weighted RPS	AEA
Q033	5	1	How is adolescent pregnancy associated with child undernutrition, maternal anemia, and the risk of NCDs such as diabetes, and hypertension of the mother?	Description	0.989	0.907	0.932	0.807	0.977	0.925	0.869
Q008	10	2	What is the impact of school-based comprehensive sexuality education given to young adolescents on prevention of gender-based violence, unsafe abortion, and smooth transitioning to adolescence from childhood?	Description	0.946	0.886	0.933	0.889	0.943	0.920	0.872
Q020	11	3	What are the barriers faced by health care provider to provide mental health services among adolescents?	Delivery	0.951	0.898	0.931	0.885	0.922	0.919	0.861
Q031	12	4	What is the impact of adolescent pregnancy on maternal mortality/morbidity?	Description	0.958	0.896	0.927	0.894	0.904	0.917	0.854
Q007	17	5	What is the impact of introducing life skills lessons in the school educational curriculum on empowerment of adolescent girls and prevention of child marriage?	Description	0.978	0.889	0.913	0.848	0.911	0.910	0.861
Q024	18	6	What is the impact of health education on the preference of using menstrual kit among adolescent girl?	Description	0.979	0.904	0.948	0.819	0.862	0.908	0.841
Q021	25	7	What are the effects of unwanted pregnancy on peripartum depression among adolescent?	Description	0.939	0.875	0.910	0.816	0.949	0.899	0.833
Q027	27	8	How does the quality and accessibility of health care services for adolescents impact their knowledge and decision-making skills related to health?	Description	0.977	0.821	0.893	0.869	0.942	0.899	0.837
Q032	31	9	What is the most acceptable and effective strategy for educating young adolescents about adverse effects of self-termination of pregnancy’s using traditional medicine?	Development	0.922	0.867	0.889	0.864	0.943	0.896	0.817
Q018	36	10	What psychosocial interventions are available to support adolescent’s well-being and mental health in Bangladesh?	Description	0.948	0.862	0.898	0.840	0.908	0.892	0.834

NCDs – non-communicable diseases

#### Fertility theme

The highest weighted RPS was 0.939 among the 38 RQs of this theme, whereas the lowest weighted RPS was 0.634. **Table 6** denotes the top 10 prioritised questions within fertility theme. Nine RQs were entered into the top 10 RQs of fertility theme, apart from the only one previously selected RQs into the overall top 20 RQs. The newly selected nine RQs from the top 10 RQs within the fertility theme were focused on menstrual regulation among reproductive aged women (Q32), women's access to sexual and reproductive health services (Q47), Fertility services (Q50, Q53, and Q75), access to contraception (Q57) and family planning (Q65, Q72 and Q73). Four out 10 RQs were categorised as description/epidemiological research, three as developmental research, two as discovery research, and the remaining one as delivery research.

**Table 6:** Top 10 research questions by Fertility theme with domain, scores for each criterion, weighted research priority score (RPS), and average expert agreement (AEA)

Question No.	Overall rank	Theme wise rank	Research question	Domain	Answerability intermediate RPS	Deliverability intermediate RPS	Effectiveness intermediate RPS	Equity intermediate RPS	Maximum impact on burden intermediate RPS	Weighted RPS	AEA
Q055	7	1	What key factors hinder or support birth spacing strategies for newlywed and underage married women, and how can they be integrated into health care systems?	Delivery	0.958	0.872	0.957	0.926	0.904	0.924	0.885
Q088	32	2	What is the knowledge, attitude and practice of menstrual regulation among reproductive aged women in Bangladesh?	Description	0.977	0.860	0.886	0.841	0.909	0.895	0.831
Q081	47	3	What would be the most cost-effective, affordable, and feasible package of interventions for influencing women's access to sexual and reproductive health services?	Development	0.956	0.867	0.889	0.822	0.864	0.882	0.804
Q078	50	4	What strategies can be implemented to make infertility services more available, accessible, and affordable for marginalized populations?	Discovery	0.926	0.865	0.906	0.819	0.875	0.881	0.824
Q082	53	5	What is the role of gender in fertility preferences and practices, and how does it influence access to sexual and reproductive health services for women?	Description	0.949	0.840	0.888	0.823	0.883	0.878	0.796
Q059	57	6	What are the most effective strategies to increase access to contraception in low resource settings like Bangladesh?	Development	0.927	0.840	0.906	0.802	0.880	0.874	0.810
Q085	65	7	What are the impacts of measurement of the quality index indicators (good counselling, privacy and confidentiality during service provision, infection control and waste management) on maintaining quality of PPFP services?	Description	0.939	0.854	0.890	0.793	0.854	0.869	0.795
Q083	72	8	What is the impact of postpartum family planning (PPFP) focused community awareness meeting among pregnant women and PPFP-focused training among service providers on PPFP services?	Description	0.956	0.844	0.856	0.822	0.844	0.866	0.809
Q065	73	9	What strategies can be adapted to improve the quality of care regarding family planning across different tiers of health systems?	Discovery	0.911	0.856	0.856	0.822	0.880	0.866	0.788
Q067	75	10	What would be the most cost-effective, affordable, and feasible package of interventions for infertility among general and working women?	Development	0.927	0.859	0.878	0.798	0.848	0.865	0.780

PPFP – Postpartum family planning

#### GI theme

Among 30 RQs in gynaecological issues, the weighted RPS ranged from 0.930 (highest) to 0.730 (lowest). **Table 7** denotes the top 10 prioritised questions within GI theme. Eight RQs were entered into the top 10 RQs of GI theme, apart from the only one previously selected RQs into the overall top 20 RQs. The newly selected eight RQs from the top 10 RQs within the GI theme were focused on gynaecological education (Q29), gynaecological cancer management (Q41, Q60, Q81 and Q89) and menstrual health (Q46, Q79, Q83). Among the top 10 RQs, five were categorised as descriptive/epidemiological research, three were innovative research, and remaining two were research to deliver, finance, or scale up.

**Table 7:** Top 10 research questions by Gyneocological Issue theme with domain, scores for each criterion, weighted research priority score (RPS), and average expert agreement (AEA)

Question No.	Overall Rank	Theme wise Rank	Research question	Domain	Answerability intermediate RPS	Deliverability intermediate RPS	Effectiveness intermediate RPS	Equity intermediate RPS	Maximum impact on burden intermediate RPS	Weighted RPS	AEA
Q103	13	1	What are the challenges, key factors, and enablers of implementing HPV testing for cervical cancer screening?	Delivery	0.967	0.864	0.944	0.884	0.915	0.915	0.878
Q098	16	2	What strategies can be implemented to scaling up the early detection of gynecological cancers in Bangladesh?	Discovery	0.946	0.898	0.933	0.860	0.912	0.912	0.888
Q117	29	3	What is the impact of school or academic institution’s support on adolescents in dealing with their gynecological concerns?	Description	0.933	0.889	0.889	0.859	0.897	0.897	0.855
Q090	41	4	What is the cost-effectiveness of different gynecological cancer screening approaches in Bangladesh considering the local health system and socio-economic context?	Description	0.939	0.869	0.917	0.817	0.887	0.887	0.821
Q110	46	5	What effects do free or low-cost menstrual supplies have on the health of women who experience menstruation?	Description	0.979	0.819	0.902	0.851	0.884	0.884	0.829
Q091	60	6	What is the impact of a cancer screening registry (collects, utilizes, and stores cancer screening data on individuals) on program management and reporting?	Description	0.905	0.841	0.913	0.829	0.872	0.872	0.807
Q111	79	7	What are the optimal tools, instruments, approaches, or measures for assessing the impact of interventions addressing menstrual health across different programmatic levels (*e.g*. local, national, global)?	Discovery	0.907	0.837	0.900	0.773	0.862	0.862	0.795
Q104	81	8	What are the potential strategies to scale-up human papillomavirus (HPV) vaccination in Bangladesh?	Discovery	0.891	0.849	0.878	0.778	0.861	0.861	0.799
Q113	83	9	How can information on menstruation be integrated into existing formal and non-formal educational curriculam, health services (*e.g*. contraceptive services, HPV vaccination, FGM support, psychosocial support), social norms, and gender equality interventions/programmes?	Delivery	0.927	0.778	0.915	0.798	0.860	0.860	0.774
Q101	89	10	What are the prevalence and factors associated with the human papillomavirus (HPV) infection among reproductive-aged women in Bangladesh?	Description	0.946	0.807	0.856	0.811	0.856	0.856	0.796

HPV – human papillomavirus

#### MNH theme

The highest weighted RPS of MNH was 0.947, whereas the lowest was 0.752. **Table 8** denotes the top 10 prioritised questions within MNH theme. Four RQs were entered into the top 10 RQs of MNH theme, apart from the six previously selected RQs into the overall top 20 RQs. The newly selected four RQs from the top 10 RQs within the MNH theme were focused on MNH services in hard-to-reach areas (Q22), ANC and PNC (Q26), depression screening (Q28) and premature and low birth weight (Q30). Six RQs were categorised as descriptive/epidemiological research and remining four was categorised as delivery or research to deliver, finance, or scale up.

**Table 8:** Top 10 research questions by Maternal and Neonatal Health theme with domain, scores for each criterion, weighted research priority score (RPS), and average expert agreement (AEA)

Question No.	Overall Rank	Theme wise rank	Research question	Domain	Answerability intermediate RPS	Deliverability intermediate RPS	Effectiveness intermediate RPS	Equity intermediate RPS	Maximum impact on burden intermediate RPS	Weighted RPS	AEA
Q151	2	1	What barriers hinder implementing Newborn Stabilizing Units in upazila hospitals?	Delivery	0.981	0.894	0.940	0.922	0.922	0.932	0.891
Q152	4	2	What is the impact of the Postpartum Haemorrhage (PPH) bundle approach on the management of PPH cases?	Description	0.971	0.910	0.912	0.890	0.942	0.925	0.890
Q122	6	3	Does the enhancement of counseling on danger signs during antenatal care (ANC) contribute to a significant reduction in adverse birth outcomes?	Description	0.952	0.912	0.923	0.902	0.933	0.925	0.895
Q141	8	4	What is the burden and risk factors for mental health issues among pregnant women?	Description	0.980	0.875	0.929	0.875	0.958	0.924	0.893
Q132	9	5	What are the bottlenecks, barriers, and challenges in identifying and managing high-risk pregnancies in low- and middle-income countries like Bangladesh?	Delivery	0.971	0.882	0.933	0.880	0.942	0.922	0.868
Q163	15	6	What are the most effective interventions for the prevention and treatment of low birth weight infants?	Description	0.950	0.880	0.929	0.878	0.920	0.912	0.867
Q147	22	7	What are the current availability, quality, and accessibility of maternal and newborn health (MNH) services in hard-to-reach areas?	Description	0.972	0.849	0.894	0.865	0.942	0.904	0.874
Q124	26	8	What is the status, impacts, and barriers of antenatal and postnatal care seeking of newborn both at facility and community?	Delivery	0.948	0.872	0.906	0.867	0.898	0.899	0.846
Q121	28	9	How does the implementation of depression screening tools in antenatal and postnatal care affect the early detection of peripartum depression?	Description	0.939	0.865	0.924	0.851	0.906	0.899	0.845
Q168	30	10	What strategy can be adapted to improve newborn interventions (ENC, KMC, Newborn Signal Functions *etc*.) for providing better support for premature and low birth weight newborns across all levels of health care facilities?	Discovery	0.934	0.863	0.892	0.882	0.913	0.896	0.833

ENC – Essential newborn care, KMC – Kangaroo mother care

#### SHRKP theme

Among 29 RQs, the weighted RPS ranged from 0.961 (highest) to 0.723 (lowest). **Table 9** denotes the top 10 prioritised questions within SRHKP theme. Five RQs were entered into the top 10 RQs of SRHKP theme, apart from the five previously selected RQs into the overall top 20 RQs. The newly selected five RQs from the top 10 RQs within the SRHKP theme were focused on discrepancy between policy and the grounded reality regarding SRHR of KPs (Q21), improving SRHR of KPs (Q23), socio-structural challenges of KP’s SRHR (Q24), HIV testing services among KPs (Q38) and access to SRH services for socially neglected lesbian, gay, bisexual, transgender, queer or questioning (LGBTQ) individuals (Q40). Based on the research domain, five out of 10 were categorised as discovery research; three were description/epidemiological research; and the remaining two were delivery research.

**Table 9:** Top 10 research questions by Sexual and Reproductive Health of Key Populations theme with domain, scores for each criterion, weighted research priority score (RPS), and average expert agreement (AEA)

Question No.	Overall Rank	Theme wise Rank	Research question	Domain	Answerability intermediate RPS	Deliverability intermediate RPS	Effectiveness intermediate RPS	Equity intermediate RPS	Maximum impact on burden intermediate RPS	Weighted RPS	AEA
Q193	1	1	How can stigma and discrimination against Key Populations be reduced to improve their sexual and reproductive health?	Discovery	0.962	0.913	0.971	0.913	0.962	0.945	0.906
Q169	3	2	What are the obstacles in raising awareness about the Sexual and Reproductive Health Rights (SRHR) of Key Populations (KPs) in Bangladesh?	Delivery	0.982	0.891	0.909	0.891	0.963	0.926	0.882
Q183	14	3	What strategies need to be implemented to get better sexual and reproductive health & family planning service from government health system by key populations (KP)?	Discovery	0.931	0.888	0.923	0.865	0.962	0.914	0.870
Q192	19	4	How do stigma and discriminatory practices impact the sexual and reproductive health behaviors of Key Populations?	Description	0.958	0.880	0.880	0.880	0.930	0.905	0.842
Q170	20	5	What challenges do Key Populations encounter in the diagnosis of COVID-19, treatment when COVID-19 positive, and the administration of vaccination against COVID-19?	Delivery	0.969	0.833	0.930	0.896	0.890	0.904	0.848
Q197	21	6	What is the current situation regarding the Sexual and Reproductive Health and Rights (SRHR) of Key Populations (KPs) in Bangladesh and the discrepancy between policy and the grounded reality?	Description	0.949	0.867	0.908	0.854	0.939	0.904	0.859
Q196	23	7	What types of interventions and/or new technologies can be used to help key populations overcome the obstacles that threaten their sexual and reproductive health right (SRHR) outcomes?	Discovery	0.957	0.909	0.880	0.819	0.936	0.902	0.835
Q195	24	8	To what extent do key populations face socio-structural challenges that put their sexual and reproductive health right (SRHR) outcomes in jeopardy?	Description	0.940	0.896	0.878	0.888	0.908	0.901	0.833
Q179	38	9	What strategies and interventions can be adapted to improve access to and uptake of HIV testing services among Key Populations (KPs)?	Discovery	0.942	0.861	0.896	0.837	0.904	0.889	0.817
Q186	40	10	What strategy can be adapted to increase the access of socially neglected lesbian, gay, bisexual, transgender, queer or questioning (LGBTQ) individuals towards SRH services?	Discovery	0.915	0.856	0.913	0.844	0.902	0.887	0.821

LGBTQ – lesbian, gay, bisexual, and transgender

In addition, top-ranked research questions based on theme-wise experts’ input have been presented in Table S3–9 in the **Online Supplementary Document**. Table S10 in the **Online Supplementary Document** represents the scoring response patterns to assess how participation varied across research themes. Among the 155 experts who completed the scoring round, 45.2% scored questions from more than one theme, while 20.7% scored all five themes. Approximately 11.6% of scorers completed all 197 research questions. Additionally, partial scoring (*i.e*. leaving some questions unanswered due to lack of expertise) was most common in the GI and SRHKP themes, which also had fewer expert participants (Table S10 in the **Online Supplementary Document**). The proportion of blank responses was higher in these two themes, possibly reflecting lower familiarity or comfort in assessing questions in these domains. In addition, to enhance the utility of our findings for policymakers, a matrix mapping each of the top-priority research questions to specific SDG targets has been developed (Table S11 in the **Online Supplementary Document**), providing a clear pathway for integrating research priorities into national planning and monitoring efforts.

## DISCUSSION

The present study aimed to identify health research priorities related to SRHR in Bangladesh using the stakeholder-led CHNRI methodology. This well-established and validated approach facilitated a transparent process, incorporating a large number of participants, broader themes, and comprehensive criteria to identify research priorities. With only six years remaining to achieve the SDGs, Bangladesh requires special attention to improve the current situation and reach targets related to neonatal mortality, fertility and reproductive health, family planning, ANC services, and essential newborn care (ENC) services [13]. Additionally, Bangladeshi women encounter challenges and disadvantages across various aspects of their lives, including access to health care, employment opportunities, political participation, and financial autonomy [37]. Adolescent fertility, early marriage, and STDs are additional concerning issues for SRHR in Bangladesh [11,12,38]. Furthermore, sex workers, including female, male, and transgender individuals, face difficulties in earning their livelihoods and are susceptible to engaging in risky sexual behaviours [16], underscoring the scope of the unfinished SRHR agenda in Bangladesh. Therefore, the objective of this priority-setting exercise was to identify research questions that reflect the knowledge gaps requiring attention for accelerating progress in the selected themes in Bangladesh during the SDG era.

Among the top 20 RQs, only three questions pertained to the fertility and GI theme. A deeper analysis revealed an intriguing explanation for this observation. During the survey, we requested technical experts to specify their areas of expertise. Upon analysing the cross-tabulation of variables such as ‘area of expertise’ and ‘Theme-wise Participant Count’ we observed that fewer experts specialising in fertility (15 participants) and GI (12 participants) participated in scoring questions related to these themes (Table S10 in the **Online Supplementary Document**). Consequently, the RPS for questions within these themes was lower. In contrast, for AH, MNH and SRHKP themes, a higher number of experts (ranging from 36 to 62) participated in scoring, resulting in higher RPS for questions in those themes. Consequently, a greater number of questions from these themes made their way into the top 20 RQs. In addition, the Global Burden of Disease (GBD) study highlighted significant reductions in adolescent, maternal, and neonatal mortality between 1990 and 2015, however, progress has been uneven [39]. Countries with a lower sociodemographic index (SDI) contributed to a larger proportion of the mortality burden in 2015 compared to 1990. The majority of these deaths were concentrated in sub-Saharan Africa and South Asia, including Bangladesh. This disparity may explain why stakeholders in Bangladesh prioritised these themes, resulting in the highest number of RQs in these areas [40,41]. Moreover, national-level data further validate the alignment of top-ranked research questions with SRHR needs in Bangladesh. For instance, the BDHS 2022 reports high adolescent fertility (83 per 1000) and low modern contraceptive knowledge (47%), indicating unmet youth needs [42]. Similarly, Gaps in maternal and neonatal care persist, with only 21% receiving quality ANC, 50% of deliveries in facilities, and neonatal deaths comprising over 60% of under-five mortality [42,43]. These issues align with priority questions on ANC quality, PPH management, NSU, and care for low-birth weight infants. Additionally, structural inequities, such as stigma against KPs, limited mental health care access, and low cervical cancer screening uptake are also reflected in the prioritised questions, reinforcing their relevance for policy and programmes.

Furthermore, among the top 20 RQs, half were categorised under description domain emphasising a strong focus on understanding the current state of SRHR issues in Bangladesh. This prioritisation reflects a continued need to address foundational knowledge gaps, likely driven by persistent structural barriers to SRHR services. Prior studies underscore the critical role of robust descriptive data in enabling policymakers to develop context-specific and effective public health interventions [44]. In addition, the complete absence of priority from the development domain in the top 20 list is striking. Development research typically involves scaling innovations, establishing evidence-based guidelines, and refining existing interventions to ensure broader applicability and sustainability. Additionally, in our analysis, we observed that such questions, which involve health system innovation and policy translation, may have been undervalued due to limited exposure among respondents, mostly researchers or academics. These roles may prioritise discovery and descriptive research over systems-level or implementation scale-up studies. Furthermore, development questions may have been perceived as less feasible or contextually applicable within existing resource constraints, leading to lower scores in deliverability and impact criteria. This gap may reflect limited attention to systems-level changes required to achieve long-term progress in SRHR. Evidence from global contexts highlights that neglecting this domain can hinder the scalability and sustainability of even the most promising SRHR interventions [45]. This finding calls for a re-evaluation of current research priorities, particularly in the context of Government of Bangladesh’s (GoB) commitments to achieving the SDG by 2030.

The top ranked RQs are closely aligned with the several SDGs targets, particularly SDG 3, 5, and 10. For instance, RQs addressing stigma, discriminatory practices, and barriers to access SRHR for KPs contribute to SDG 10 by seeking to reduce inequalities and ensure inclusivity in health services. Similarly, questions on adolescent pregnancy, maternal health, and newborn care directly relate to SDG 3, which emphasises reducing maternal mortality and ensuring universal access to reproductive health care. Efforts to promote life skills education, comprehensive sexuality education, and awareness about SRHR support SDG 5 by empowering women and girls and reducing harmful practices such as child marriage. Moreover, RQs focusing on mental health, HPV screening, and the prevention and management of conditions like postpartum haemorrhage, stunting, and non-communicable diseases (NCDs) address SDG 3′s broader agenda of strengthening health systems and promoting well-being at all ages. By tackling key barriers, bottlenecks, and facilitators to effective health interventions, these RQs serve as a foundation for achieving equitable and sustainable health outcomes, consistent with GoB’s commitments to the 2030 Agenda for Sustainable Development [46].

Our findings show important alignment with other CHNRI exercises conducted in LMICs. Adolescent pregnancy and child birth, empowerment and gender-based violence (GBV) were the topics of top ranked question of AH theme. Similar finding was also observed in a study conducted in LMICs using CHNRI method, where the top ranked research question focused on optimising health care service care for pregnant adolescent girls [47]. A clear manifestation of early pregnancy and child birth and violence against adolescent girls, is the high prevalence of child marriage in Bangladesh – according to the most recent BDHS (2012) 50% of women aged 20-24 years were married before the age of 18 [13]. Additionally, a CHNRI priority-setting conducted in Kenya for adolescent sexual and reproductive health (SRH) highlighted interventions for vulnerable adolescent subgroups, including married adolescents and those out of school [48]. This aligns with our top-ranked questions focused on adolescent pregnancy, mental health, and life-skills education, underscoring the shared burden of adolescent SRHR across LMICs. In Ethiopia, a recent CHNRI exercise focused on maternal, newborn, and child health (MNCH) also emphasised strengthening health system responsiveness, especially in underserved populations and during pregnancy and postpartum periods—similar to our prioritisation of research questions on ANC quality, PPH bundles, and care for high-risk pregnancies [49]. Fertility service and family planning were the topics of top ranked question of fertility theme. Though the total fertility rate is not high for Bangladesh, hence an increasing amount of evidence shows that fertility is one of the important factors that have impacts on reducing maternal and child mortality [50]. Another CHNRI exercise prioritised about the effective strategies to implement good quality comprehensive contraceptive services for girls regarding fertility [51]. Though this study focused on the family planning services, RQ regarding contraceptive method was not mentioned in the top prioritisation list here. Most of the questions form the GI theme was on Gynaecological care including cancer screening registry. This is the way of digitalisation of National Cervical Cancer Screening Programme of Bangladesh [52]. No previous study was conducted on this topic using CHNRI method. However, it is evidenced that poor knowledge and awareness in diagnosis and management of gynaecological cancer make the outcome worse [53]. This might be the reasons to prioritise gynaecological cancer within the top priority list by the technical expert. Most of the questions from the MNH theme were from pregnancy and childbirth area. Maternal and neonatal mortality is still high in Bangladesh and careful attention to meet the SDG targets [18]. Finding of this study is consistent with another CHNRI exercise in Ethiopian context where the experts focused on strengthening antenatal and postpartum care-seeking as well as health system [53]. The RQs from the SRHKP theme were mostly on service coverage for the KP. It is a universal challenge for the KPs [54]. Finding of this study is consistent with previous studies, where they prioritised numerous barriers to raise awareness regarding SRHR of key populations [55–57]. As the global concern, the present study highlighted on the interventions to improve access to and uptake of HIV testing services among key populations within the top 10 priorities. Similar priority as strategies and interventions to improve access, uptake, and linkage to care, and self-testing, particularly for key populations was also revealed by another CHNRI exercise [58]. Rapid acceleration is required to improve the situation and to meet the SDG targets related to the SRHKP. In contrast, a CHNRI exercise in India placed comparatively more emphasis on implementation research for community-based interventions, reflecting India's policy shift toward integrated service delivery platforms like Health and Wellness Centres [59]. Our prioritisation was more focused on identifying structural barriers (*e.g*. stigma for key populations, adolescent mental health) and scaling up specific service components (*e.g*. HPV testing, NSU implementation), likely due to the persistent inequalities and service coverage gaps in Bangladesh.

Overall, participating experts have prioritised RQs primarily aimed at improving the SRHR situation in Bangladesh. The survey results have strongly prioritised intervention research to understand barriers and challenges and assess effectiveness and uptake of evidence-based interventions combined with new innovative intervention. This aligns with the findings from other CHNRI exercises, where research questions related to interventions were given higher priority compared to other types of research, mainly due to their potential to directly address the disease burden in low-resource settings such as Bangladesh [20]. The positive association between AEA and RPS also indicates substantial agreement in the high ranked priorities among experts, which was also similar with another study conducted in Bangladesh and LMICs [31].

The CHNRI approach has limitations in identifying complex, structural, or rights-based research priorities, as its scoring criteria. The scoring criteria, particularly answerability and deliverability, may unintentionally disadvantage questions like legal reform, gender-based violence, or health system accountability, even if these are crucial to advancing SRHR [24,60]. These areas, though crucial for advancing SRHR, may receive lower priority scores due to perceived challenges in immediate implementation. This methodological feature has been observed in prior CHNRI exercises as well, where technical feasibility often outweighed normative or structural concerns in priority rankings [26,60]. To address this, future exercises could incorporate additional criteria or stakeholder deliberation phases to better capture the importance of transformative and policy-oriented research. In addition, our analysis showed that expert-assigned weights reduced the influence of ‘equity’ (−15%) and ‘burden reduction’ (−9%) on final scores. This likely reflects a preference for more actionable criteria like effectiveness and deliverability but raises concerns about deprioritising structurally important SRHR research in Bangladesh context. Similar trends have been noted in previous CHNRI applications, where rights-based or transformative questions scored lower due to perceived feasibility challenges [33,60].

### Strength and limitation

There were multiple strengths to our research prioritisation exercise. This is the first study in Bangladesh to systematically document and report on a priority setting on SRHR. A key strength lies in its adaptation of the CHNRI methodology, a well-established approach, which facilitated a transparent process in identifying research priorities. Beyond transparency, the method proved to be structured, flexible, cost-effective, and conducive to online execution. Yoshida et al. found that when an expert group ranks RQs, the collective opinion stabilises rapidly, leading to a high level of reproducibility in the top 15–20 ranked questions with as few as 45–55 experts [20]. In our study, more than 50 participants contributed to each of the five themes. Additionally, we also applied five criteria to score the RQs, enhancing the overall strength of this study.

Our approach was subject to a few limitations also. During the process of eliciting study questions and assigning scores, we had low survey response rates. This is typical of this approach [20] and could lead to bias in self-selection. We listed a sizable pool of experts using both organised and unstructured approaches, and we periodically reminded and requested them to participate in the study in order to increase response rates and guarantee that a varied set of experts took part. We extended the invitation to a broader group of experts (not only those who submitted RQs) to take part in the scoring process. This approach boosted participation and provided an opportunity for individuals who did not submit RQs [20]. The majority of the experts were either researchers or academic, while the health care professionals and policy makers comprised a smaller proportion of respondents that limited the representativeness of the sample. Furthermore, during the iterative consolidation, there was a reduction in the quantity of initially submitted RQs for certain themes to enhance response rates during the scoring phase. This strategy inadvertently led to the oversight of crucial inquiries, introducing a systematic error in response options. The extensive list of RQs was also time-consuming, resulting in scorer fatigue. For the 197 research questions, a respondent would need to provide a total of 985 scores (197 × 5) if they answered all the questions, which took nearly an hour to complete. Given the substantial workload associated with scoring, we encouraged participants to focus on themes of their interest rather than attempting to assess all themes. However, approximately 45% of participants scored more than one theme and 20% scored all the five themes. Additionally, more than 10% participants scored all the 197 RQs. To mitigate preferential bias stemming from scoring fatigue, we randomised the presentation of research themes and questions to scorers, ensuring equal opportunity for each theme and question to be chosen and scored [31]. In addition to that, we chose to utilise only 5 criteria without sub-criteria for scoring RQs [47], which may lead to reduced variability in scores. Nonetheless, we made this decision to prevent scoring fatigue.

## CONCLUSIONS

In order to meet the country-specific SDGs target regarding SRHR by 2030, we should reconsider intervention research on priority to understand barriers and challenges related to SRHR services in Bangladesh. In addition to that, assessing the effectiveness and uptake of evidence-based interventions combined with new innovative interventions is also required. The findings will support the AdSEARCH project, as well as the GoB and other donors, in shaping their research agendas aimed at improving sexual and reproductive health (SRH) outcomes and advancing rights for various population groups with diverse SRH needs in Bangladesh. In addition to that, the findings will help bring further attention and secure additional funding from donors in Bangladesh. Finally, the findings will assist policymakers, researchers and funders in understanding how different research domains and questions can enhance SRHR outcomes.

## Additional material


Online Supplementary Document
