# Supplementary material for: Setting stakeholder-led research priorities for advancing Sexual and Reproductive Health and Rights in Bangladesh using CHNRI method: an icddr,b initiative
Source: J Glob Health. 2025 Jul 11;15:04186. doi: 10.7189/jogh.15.04186 (PMC12247414; doi:10.7189/jogh.15.04186)
Supplement: Online Supplementary Document [file jogh-15-04186-s001.pdf]

**Supplement to: Sayeed A, Saha N, Ameen S, Akter E, Hossain L, Hasan MM, Rahman F, Raza S, Ether ST, Shafiq SS, Mahmood HR, Saberin A, Ashrafee S, Alam HMS, Saha PK, Haider S, Sarkar S, Mahmud M, Islam MJ, Salam SS, Nahar Q, Arifeen SE, Ahmed A, Rahman AE. Setting stakeholder-led research priorities for advancing Sexual and Reproductive Health and Rights in Bangladesh using CHNRI method: an icddr,b Initiative. J Glob Health. 2025;15:04186.**

**Table S1.** Linkages between themes related to SRHR and the SDGs

| Sustainable Development Goals (SDGs) | Adolescent Health (AH) | Maternal and Neonatal Health (MNH) | Fertility (F) | Gynecological Issues (GI) | Sexual and Reproductive Health of Key Populations (SRHKP) |
|---|---|---|---|---|---|
| **SDG 3: Ensure healthy lives and promote wellbeing for all at all ages** | | | | | |
| 3·1 By 2030, reduce the global maternal mortality ratio to <70 per 100 000 livebirths | Linked | Stated | - | - | Linked |
| 3·2 By 2030, end preventable deaths of newborns and children aged <5 years, with all countries aiming to reduce neonatal mortality to at least ≤12 per 1000 livebirths and under-5 mortality to at least ≤25 per 1000 livebirths | Linked | Stated | - | Linked | Linked |
| 3·3 By 2030, end the epidemics of AIDS, tuberculosis, malaria, and neglected tropical diseases, and combat hepatitis, water-borne diseases, and other communicable diseases | Linked | Linked | - | Stated | Stated |
| 3·4 By 2030, reduce by one third premature mortality from non-communicable diseases through prevention and treatment, and promote mental health and well | - | - | - | Linked | - |
| 3·7 By 2030, ensure universal access to sexual and reproductive health-care services, including for family planning, information and education, and the integration of reproductive health into national strategies and programmes | Stated | Stated | Linked | Linked | Stated |
| 3·8 Achieve universal health coverage, including financial risk protection, access to quality essential health-care services, and access to safe, effective, quality, and affordable essential medicines and vaccines for all | Linked | Stated | Linked | Linked | Stated |
| **SDG 4: Ensure inclusive and equitable quality education and promote lifelong learning opportunities for all** | | | | | |
| 4·7 By 2030, ensure that all learners acquire the knowledge and skills needed to promote sustainable development, including education for sustainable development and sustainable lifestyles, human rights, gender equality, promotion of a culture of peace and non-violence, global citizenship, and appreciation of cultural diversity and of culture's contribution to sustainable development | Linked | - | - | - | - |
| **SDG 5: Achieve gender equality and empower all women and girls** | | | | | |
| 5·1 End all forms of discrimination against all women and girls everywhere | Linked | Linked | - | Linked | Linked |

| | | | | | |
|---|---|---|---|---|---|
| 5·2 Eliminate all forms of violence against all women and girls in the public and private spheres, including trafficking, and sexual and other types of exploitation | Linked | Linked | - | Linked | Linked |
| 5·3 Eliminate all harmful practices, such as child, early, and forced marriage, and female genital mutilation | Linked | - | - | - | - |
| 5·6 Ensure universal access to SRHR as agreed in accordance with the Programme of Action of the ICPD and the Beijing Platform for Action, and the outcome documents of their review conferences | Stated | Stated | - | Stated | Linked |
| 5.c Adopt and strengthen sound policies and enforceable legislation for the promotion of gender equality and the empowerment of all women and girls at all levels | Linked | - | - | - | - |
| **SDG 16: Promote peaceful and inclusive societies for sustainable development, provide access to justice for all, and build effective, accountable, and inclusive institutions at all levels** | | | | | |
| 16·1 Significantly reduce all forms of violence and related death rates everywhere | Linked | - | - | - | - |
| 16·2 End abuse, exploitation, trafficking, and all forms of violence against and torture of children | Linked | - | - | - | - |

**Stated** means the area is mentioned in the target or indicator language.
**Linked** means the area relates to the SDG target but is not specifically mentioned in the target. SRHR=sexual and reproductive health and rights.
SDGs=sustainable development goals.
SRH=sexual and reproductive health.
STIs=sexually transmitted infection.
ICPD=International Conference on Population and Development.
**Source:** Accelerate progress—sexual and reproductive health and rights for all: report of the Guttmacher–Lancet Commission

# Appendix S1

# Online Data Collection Platform (Phase 1)

## Prioritisation of Research Questions for Advancing the Understanding of Sexual and Reproductive Health and Rights (SRHR) in Bangladesh

# PHASE 1

### Welcome to phase-1 of the AdSEARCH research prioritisation exercise on SRHR!!!!!!!

**About AdSEARCH**

The Department of Foreign Affairs, Trade and Development (DFADT) funded the Advancing Sexual and Reproductive Health and Rights (AdSEARCH) project of icddr,b which aims to improve sexual and reproductive health (SRH) outcomes and realise rights among different population groups with distinct SRH needs in Bangladesh.

**About AdSEARCH research priority setting exercise**

One of the objectives of AdSEARCH is to Prioritisation of Research Questions for Advancing the Understanding of Sexual and Reproductive Health and Rights (SRHR) in Bangladesh. AdSEARCH research prioritisation team has adopted the **Child Health and Nutrition Research Initiative (CHNRI)** method for the research prioritisation exercise involving technical experts and stakeholders.

**Your role**

You are approached to participate in the research prioritisation exercise as a **Technical Expert** based on your expertise and significant contribution in the field of SRHR (Adolescent Health/Fertility/Maternal and Neonatal Health/Gynecological issues and sexual health/Sexual and reproductive health of key populations). The exercise requires your input in **two phases.**

- **In the first phase,** we require you to propose **Research questions** based on the theme(s) (Adolescent Health/Fertility/Maternal and Neonatal Health/Gynecological issues/Sexual and reproductive health of key populations) of interest. The first phase will take approximately 15 minutes. Your participation is completely voluntary, and you can withdraw your participation at any time during the process. Anonymity and confidentiality will be strictly maintained throughout the process. However, you will be acknowledged for your contribution in the planned scientific outputs of this research i.e., publications and reports (subject to your consent).
- We will communicate you for your further support in the **second phase.**

☐ **I agree to participate in phase 1 of the exercise after reading the consent form**

Register

Privacy Policy

For any query or assistance, please contact us at adsearch_rp@icddrb.org

In partnership with Canada icddr,b

**Layout 1:** Welcome Page of RP Research Prioritisation Exercise Phase 1

**Prioritisation of Research Questions for Advancing the Understanding of Sexual and Reproductive Health and Rights (SRHR) in Bangladesh**

**PHASE 1**

## Consent Form

**Title of the study:** Prioritisation of Research Questions for Advancing the Understanding of Sexual and Reproductive Health and Rights (SRHR) in Bangladesh

**Principal Investigator: Abu Sayeed**

**Research Organizations:** This research is part of the Advancing Sexual and Reproductive Health and Rights (AdSEARCH) research prioritisation exercise, conducted by International Centre for Diarrheal Disease Research, Bangladesh (icddr,b).

**Purpose of the research:**
Dear Sir/Mam,

We are contacting you from the International Centre for Diarrheal Disease Research, Bangladesh (icddr,b). We are conducting a research named **"Prioritisation of Research Questions for Advancing the Understanding of Sexual and Reproductive Health and Rights (SRHR) in Bangladesh"** to identify health research priorities related to SRHR in Bangladesh.

**Why have we invited you?**
Given your expertise in research related to SRHR (adolescent health / fertility/ maternal and neonatal health/ gynecological issues / sexual and reproductive health of key populations) and/or your publications in this area, we are inviting you to kindly participate in this study. Your participation will help us by providing valuable expert information related to the SRHR of Bangladesh.

**Methods and Procedures:**
If you agree to participate in this study, we will ask you to **submit one to five most promising research questions related to SRHR (adolescent health / fertility/ maternal and neonatal health/ gynecological issues/ sexual and reproductive health of key populations) of Bangladesh.** And you will be requested to submit your questions within four weeks. After that, we will send you a scoring sheet with brief instruction and a set of collated research questions. We will ask you to provide your score according to the instructions. It will take around 15 minutes to submit research questions and during the scoring of the combined research questions it will take around half an hour.

**Risks and Benefits:**
There will be no or minimal risks related to your participation in this study. The information collected from you will be kept confidential and will only be used for research purpose. Participation in this study may not benefit you directly but the information you share will be very useful for improving SRHR in Bangladesh.

**Privacy, anonymity and confidentiality:**
All information collected from you during the study will be kept strictly confidential and not shared with anyone outside the study team. The data will be coded so that the personal identity and individual data from are traceable only with the code key which will be held by the study researchers, no one else will have access to it.

**Future use of information:**
Anonymous or abstracted information and data may be shared with other researchers. However, this will not conflict with or violate the maintenance of privacy, anonymity and confidentiality of information identifying participants in any way.

**Right not to participate and withdraw:**
You have the sole authority to decide about your own participation in this study. You would also be able to withdraw from participation any time during the study, without any explanation required.

**Principle of compensation:** Your participation in this study is completely voluntary and you will not get any payment for participating in this study.

**Contact person:**
If you have questions about this study or if you feel that you have been treated unfairly or have been affected in any way by joining the study, you may communicate with the Principal Investigator of the study Abu Sayeed, Maternal and Child Health Division, icddr,b by calling Mobile: +8801753837266 .

You can also contact icddr,b secretariat, Mr. M. A. Salam Khan (Phone: (+88 02) 9827084 or PABX (+88 02) 9827001-10, ext. 3206).

If you agree to our proposal of enrolling in our study, please indicate that by adding your signature in the specified space below.

Thank you for your consideration.

**Participant:** I certify that all the above information was adequately explained to me and I understand the explanation.

☐ **I agree to participate in phase 1 of the exercise**

Submit

In partnership with Canada icddr,b

**Layout 2:** Consent Form Page for Participation

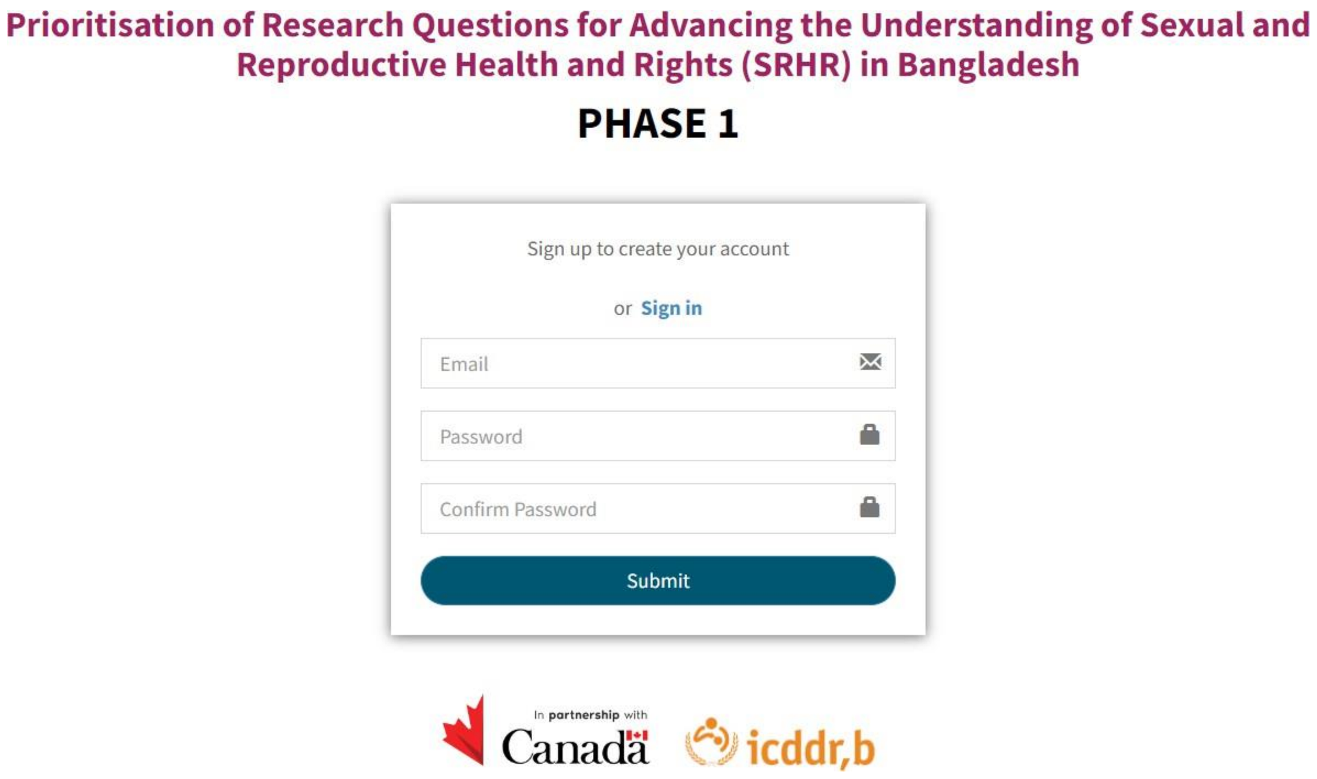


**Layout 3:** Online Registration Page (Blank)

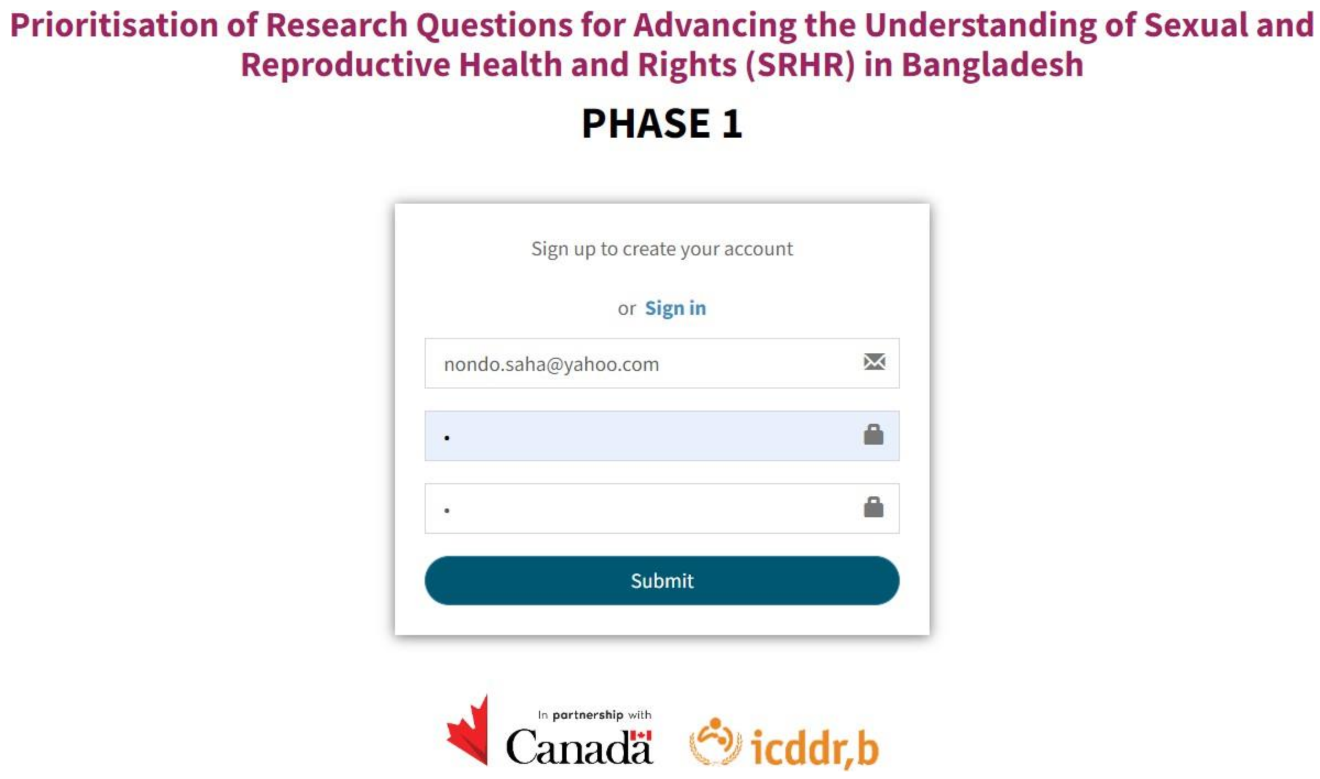


**Layout 4:** Online Registration Page (Filled)

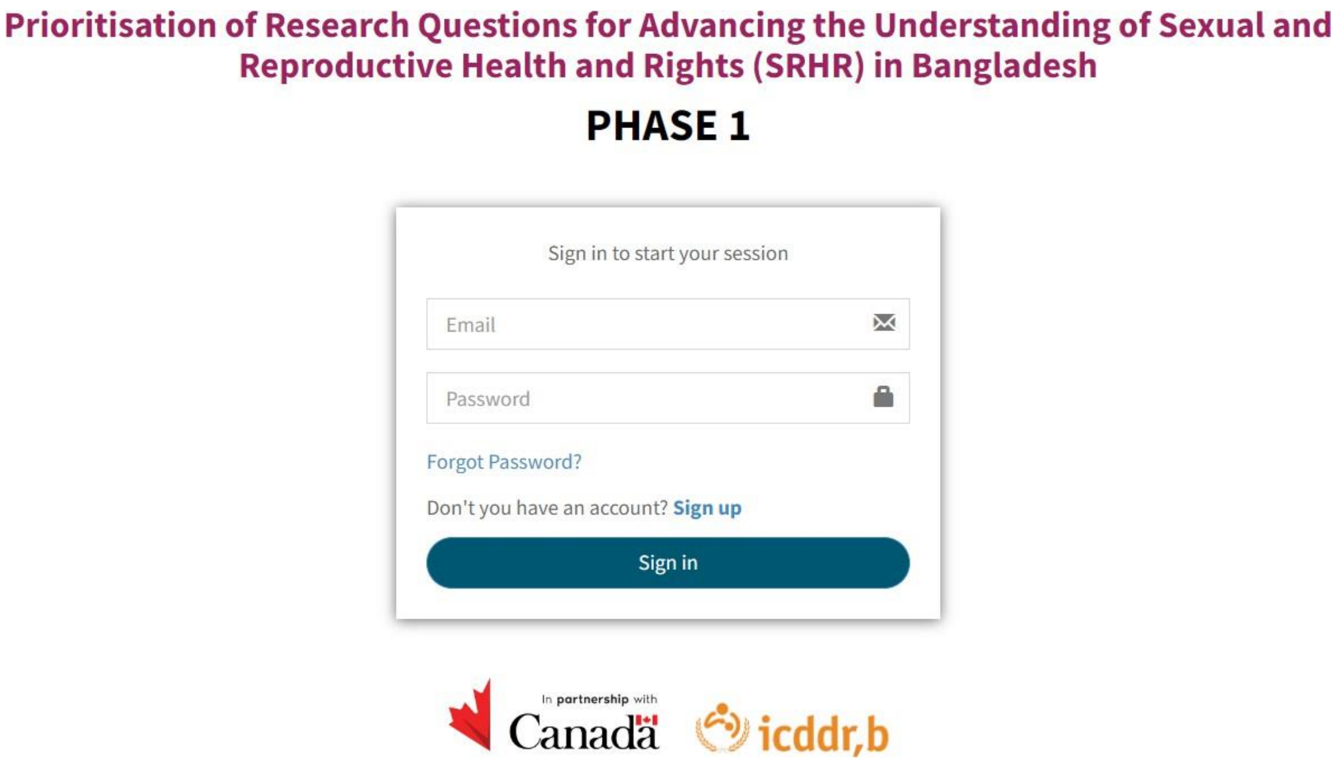


**Layout 5:** Online Log in Page (Blank)

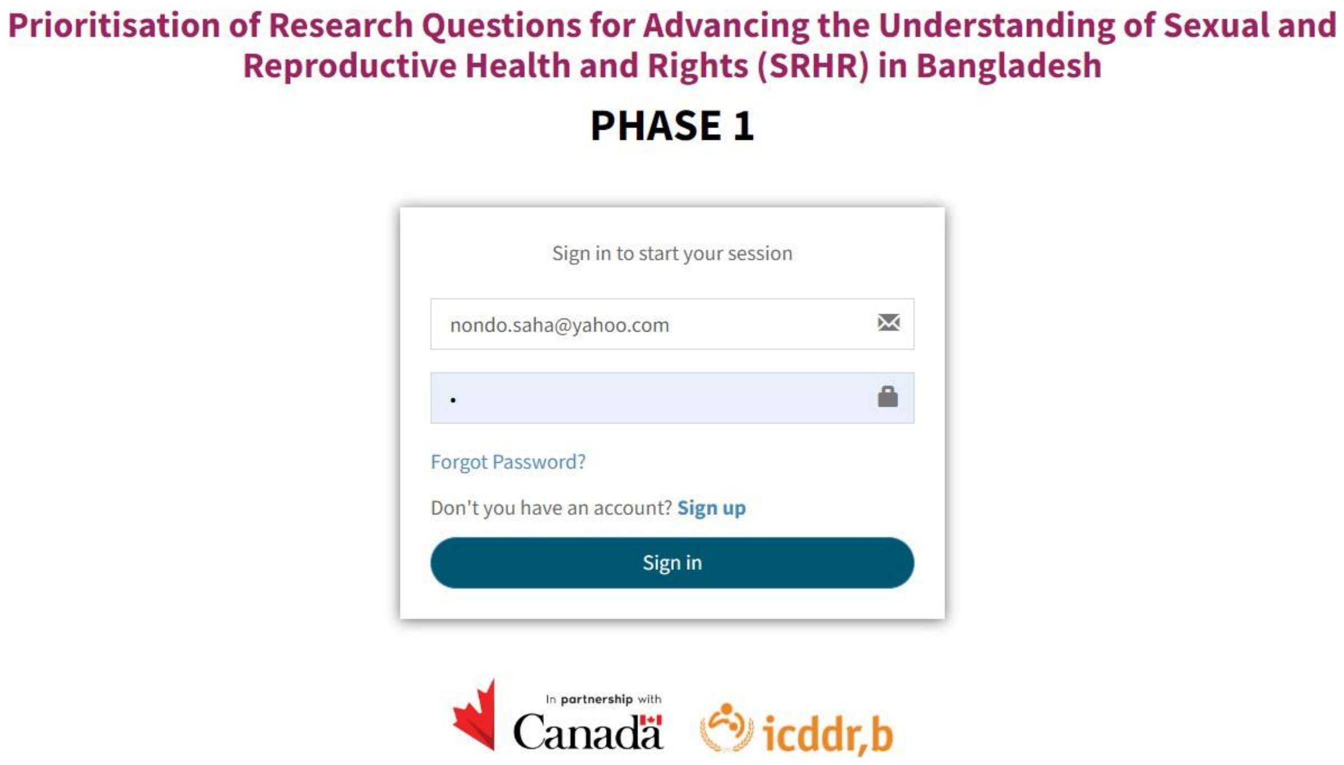


**Layout 6:** Online Log in Page (Filled)

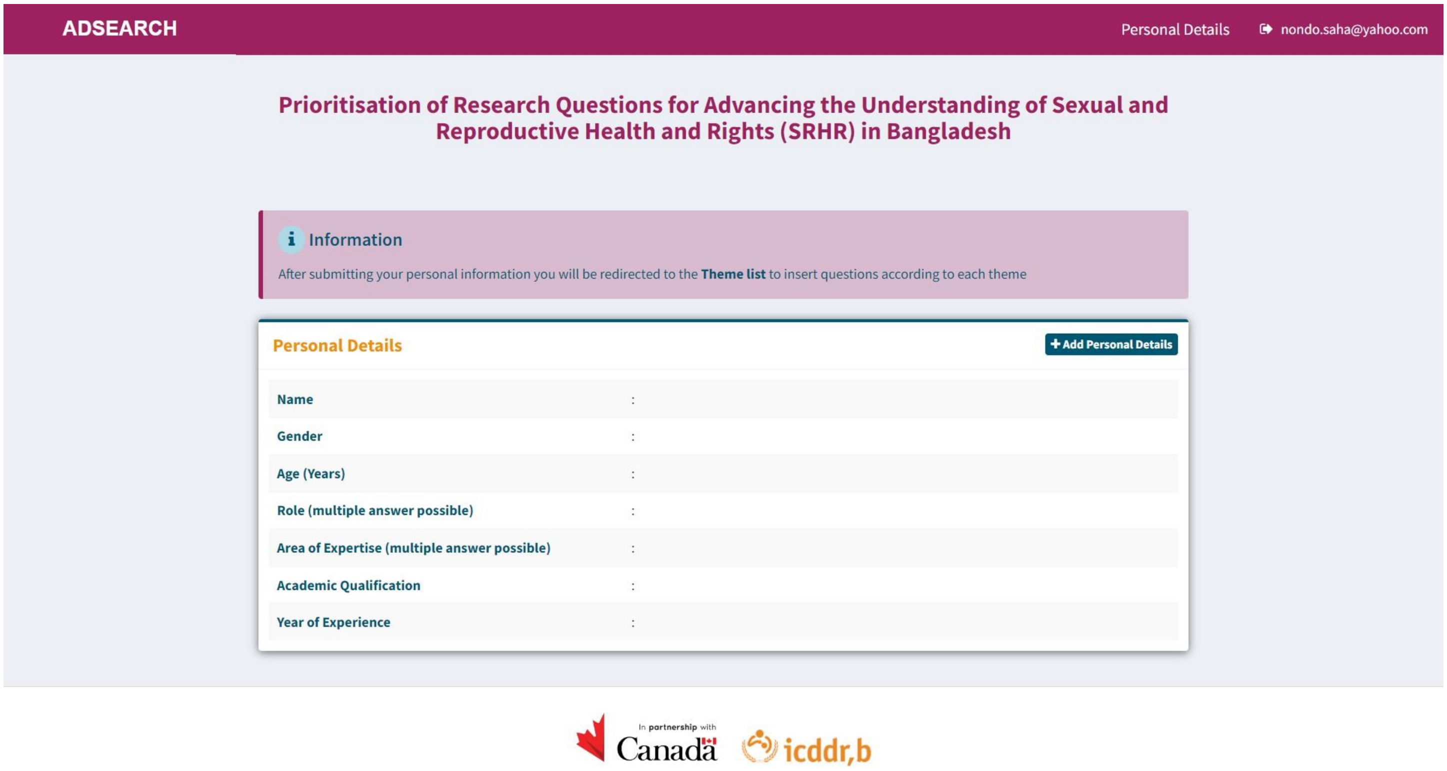


**Layout 7:** Personal details Page (Blank)

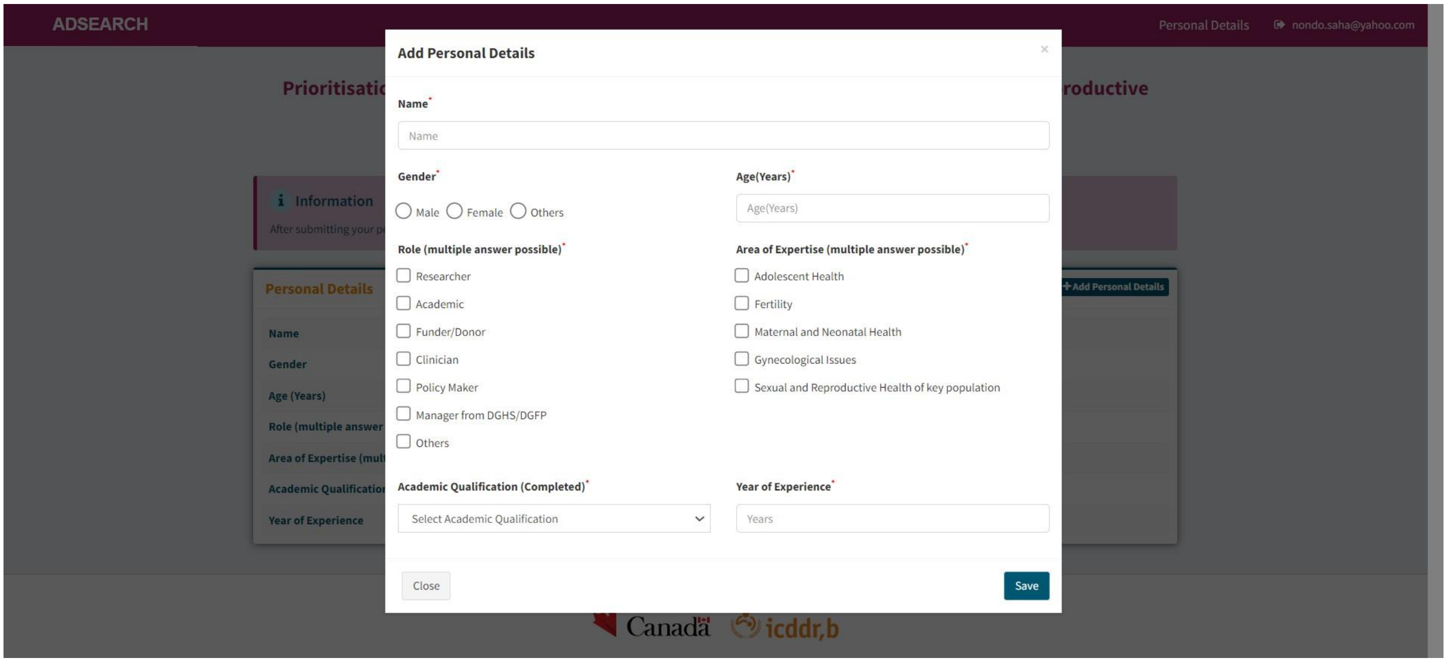


**Layout 8:** Pop UP Personal details Page (Blank)

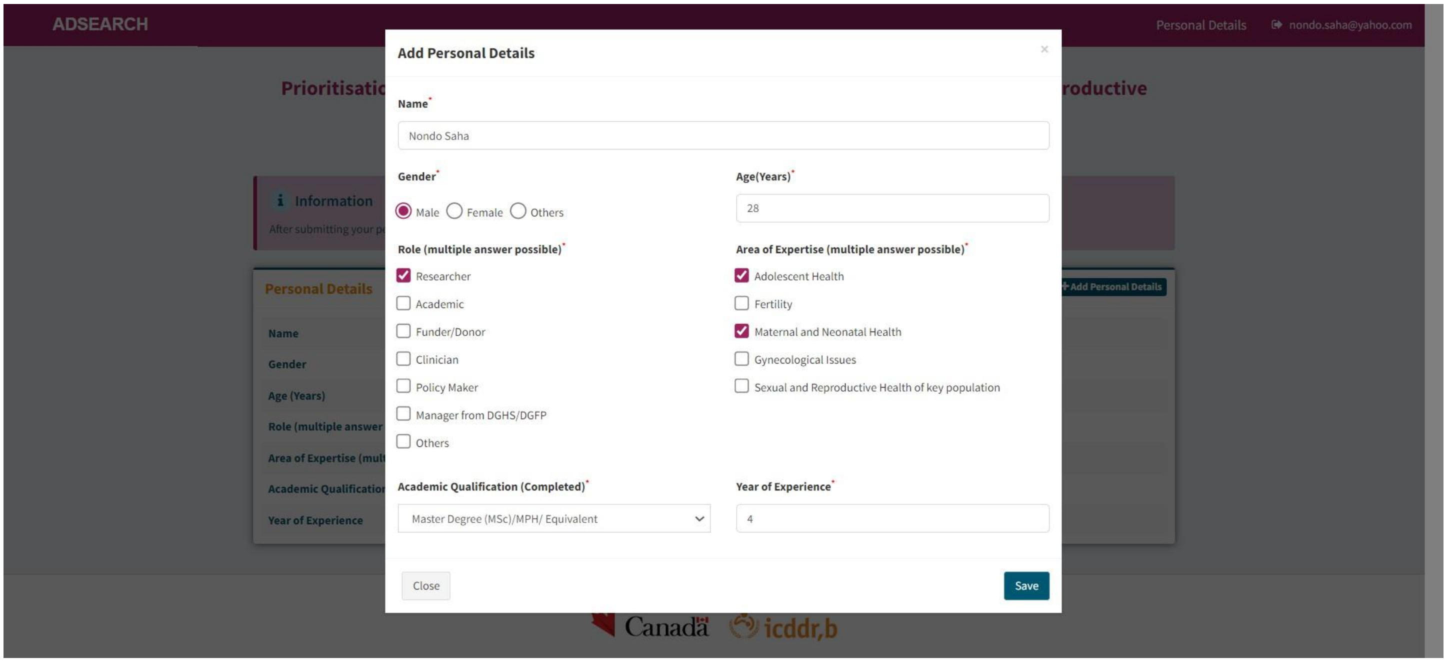


**Layout 9:** Pop UP Personal details Page (Filled)

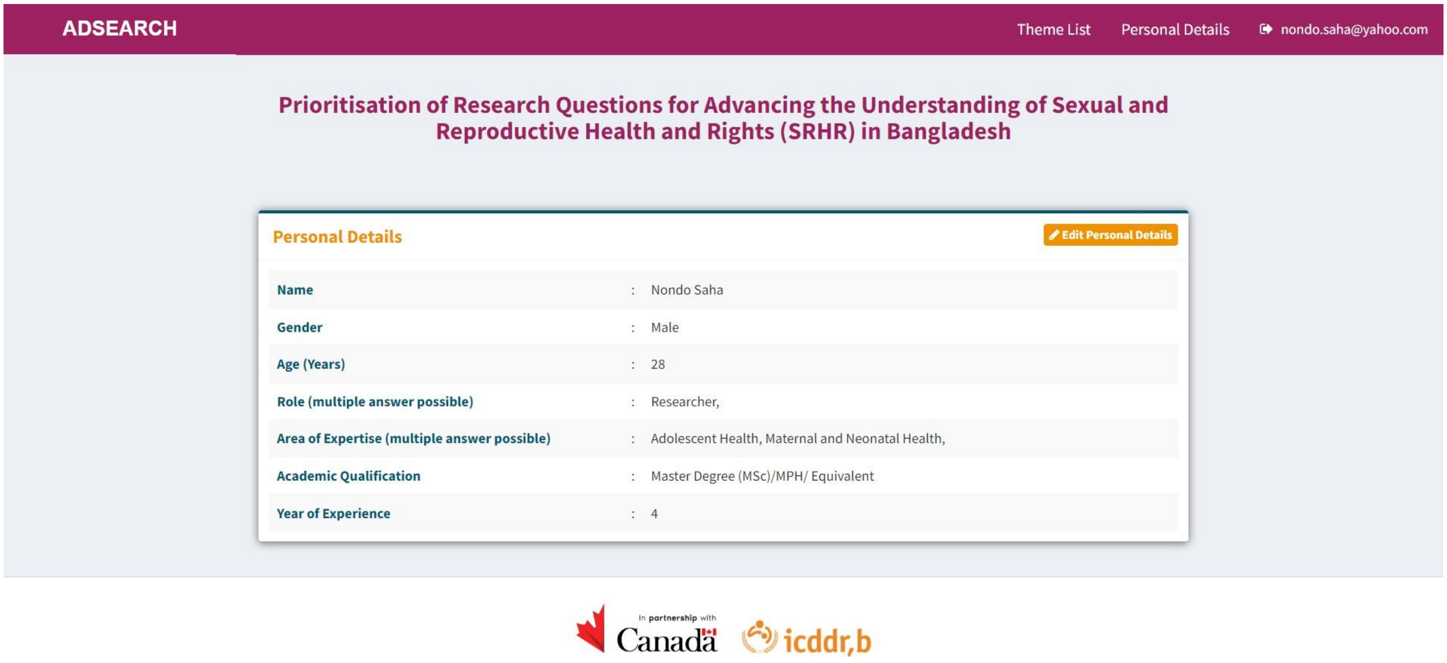


**Layout 10:** Personal details Page (Filled)

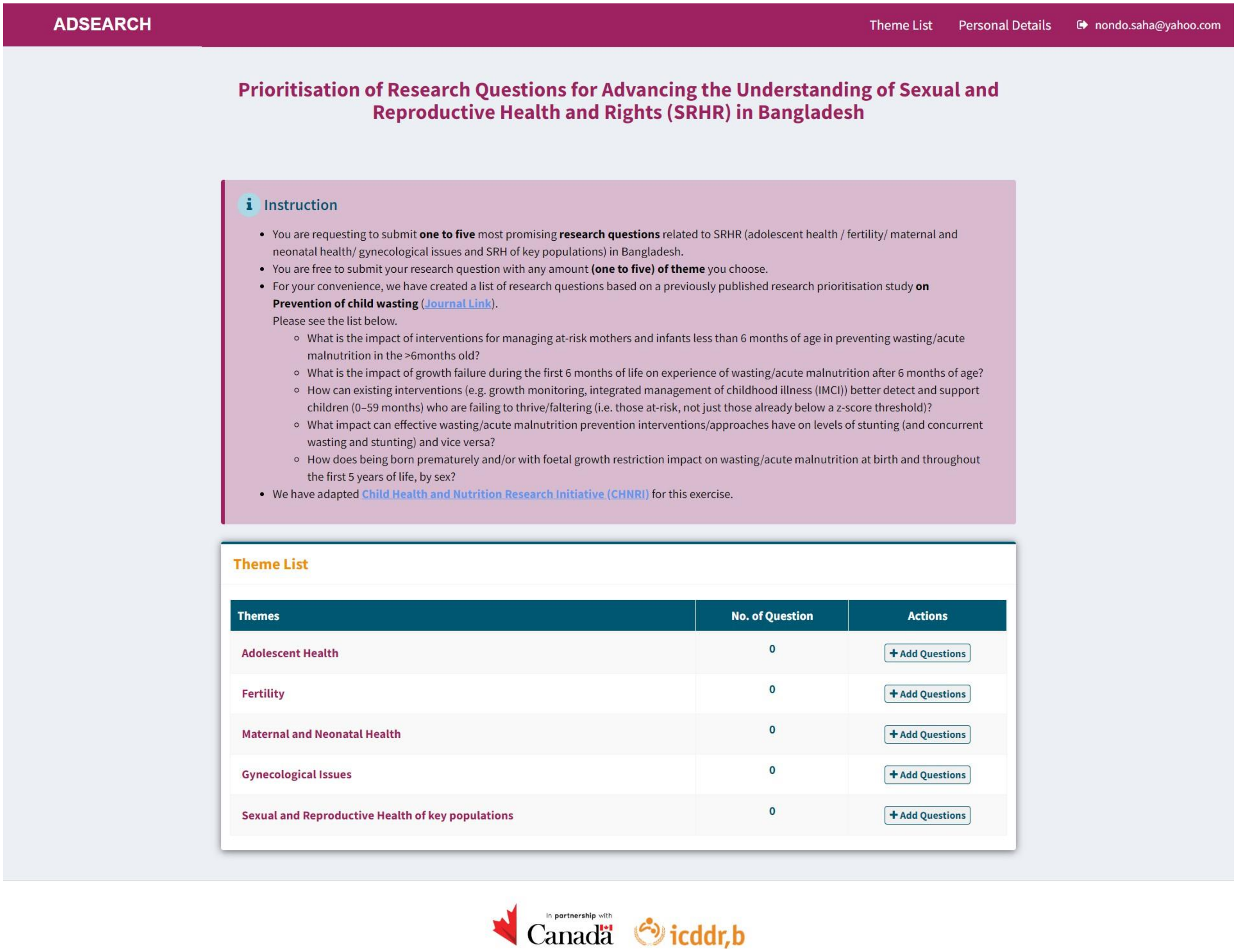


**Layout 11:** Instruction for Submitting RQs

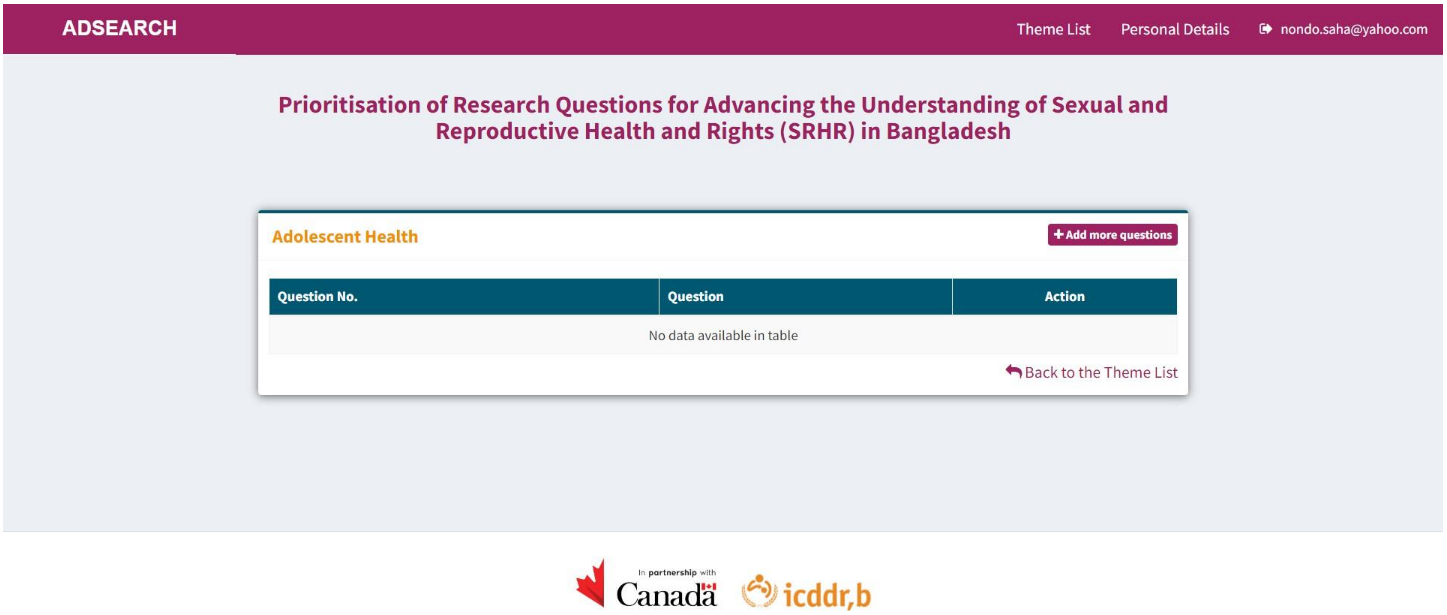


**Layout 12:** RQs Submitting Page (Blank)

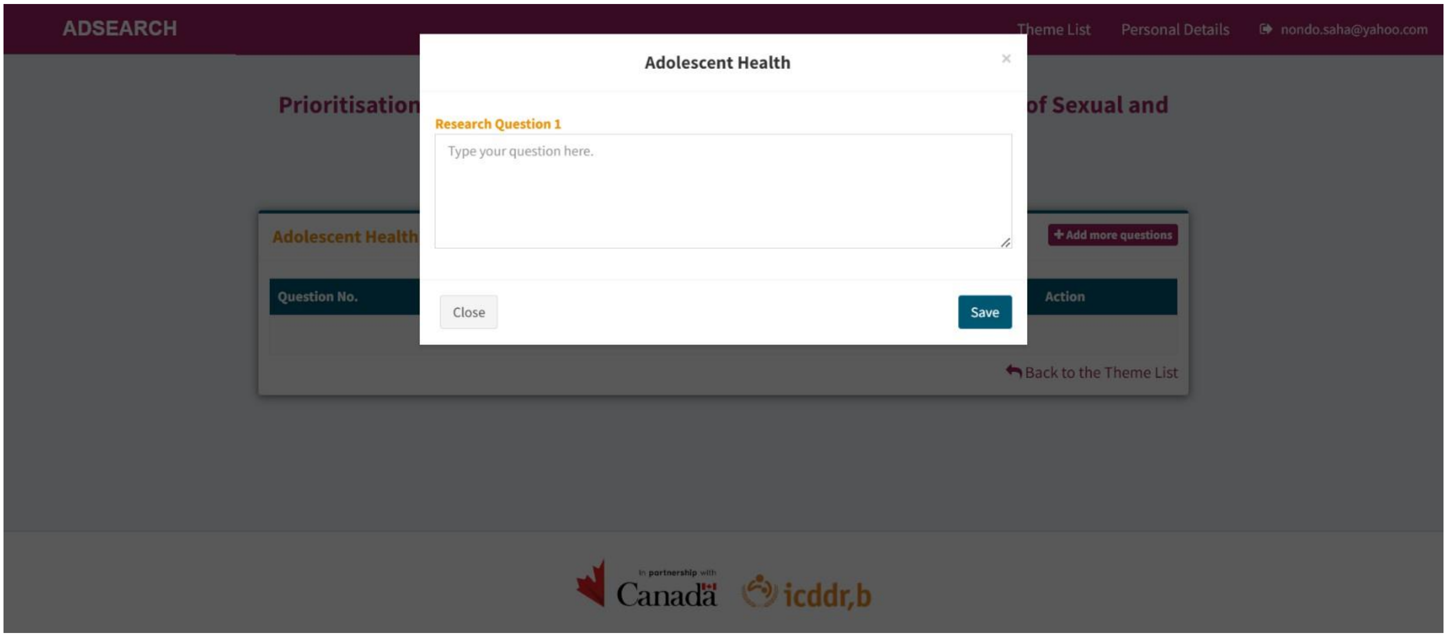


**Layout 13:** Pop Up RQs Input Page (Blank)

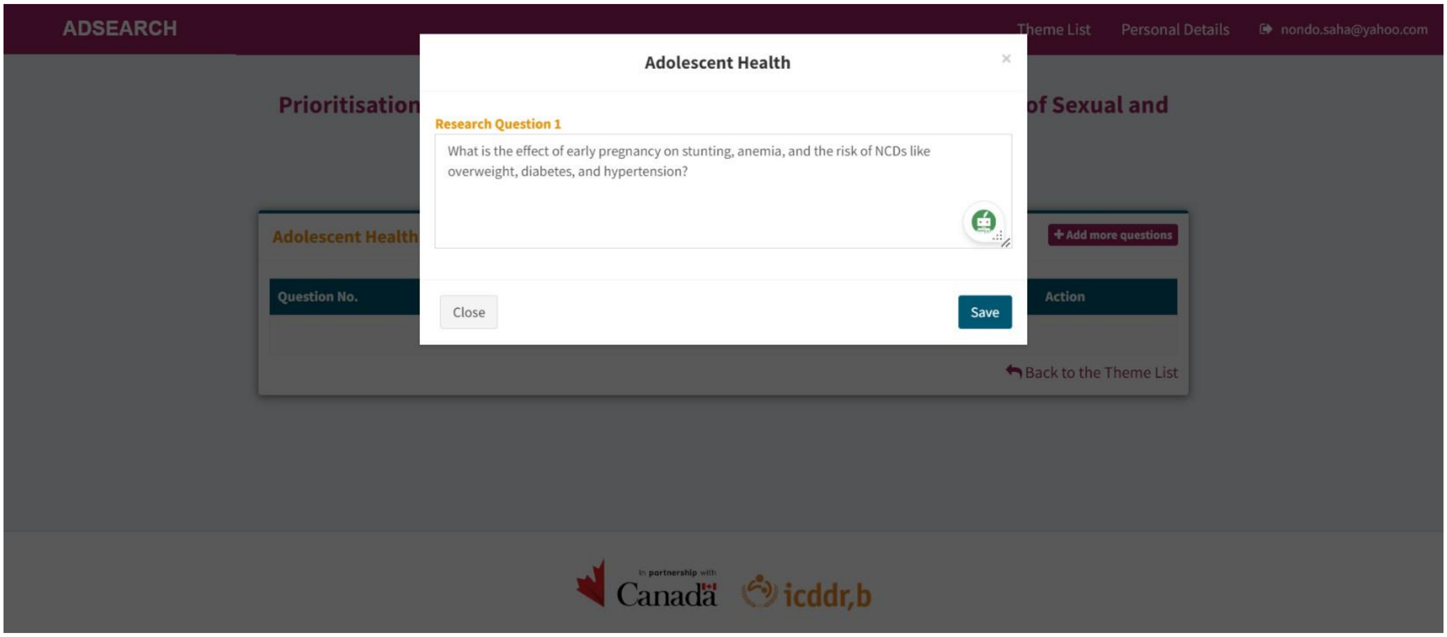


**Layout 14:** Pop Up RQs Input Page (Filled)

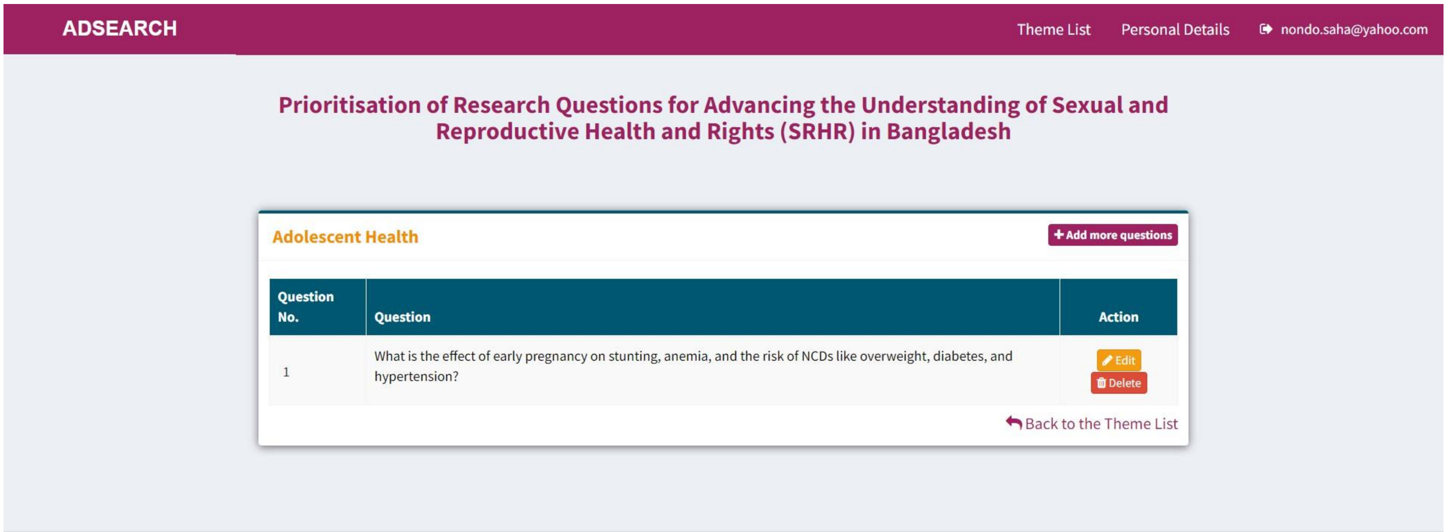


**Layout 15:** RQs Submitting Page (Filled)

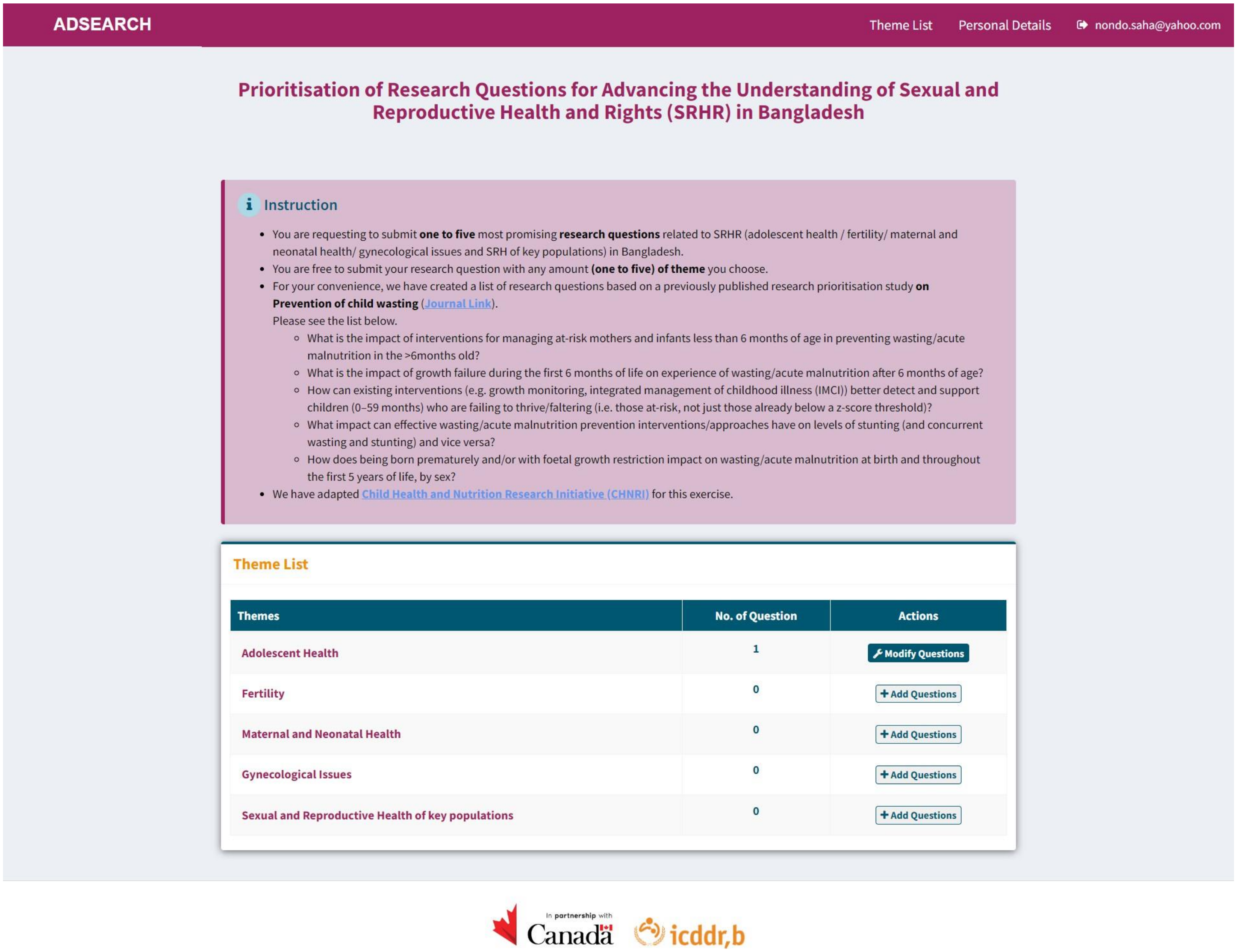

ADSEARCH

Theme List | Personal Details | nondo.saha@yahoo.com

## Prioritisation of Research Questions for Advancing the Understanding of Sexual and Reproductive Health and Rights (SRHR) in Bangladesh

### Instruction

- You are requesting to submit **one to five** most promising **research questions** related to SRHR (adolescent health / fertility/ maternal and neonatal health/ gynecological issues and SRH of key populations) in Bangladesh.
- You are free to submit your research question with any amount **(one to five) of theme** you choose.
- For your convenience, we have created a list of research questions based on a previously published research prioritisation study **on Prevention of child wasting** (Journal Link).
  Please see the list below.
  - What is the impact of interventions for managing at-risk mothers and infants less than 6 months of age in preventing wasting/acute malnutrition in the >6months old?
  - What is the impact of growth failure during the first 6 months of life on experience of wasting/acute malnutrition after 6 months of age?
  - How can existing interventions (e.g. growth monitoring, integrated management of childhood illness (IMCI)) better detect and support children (0–59 months) who are failing to thrive/faltering (i.e. those at-risk, not just those already below a z-score threshold)?
  - What impact can effective wasting/acute malnutrition prevention interventions/approaches have on levels of stunting (and concurrent wasting and stunting) and vice versa?
  - How does being born prematurely and/or with foetal growth restriction impact on wasting/acute malnutrition at birth and throughout the first 5 years of life, by sex?
- We have adapted Child Health and Nutrition Research Initiative (CHNRI) for this exercise.

### Theme List

| Themes | No. of Question | Actions |
|---|---|---|
| Adolescent Health | 1 | Modify Questions |
| Fertility | 0 | + Add Questions |
| Maternal and Neonatal Health | 0 | + Add Questions |
| Gynecological Issues | 0 | + Add Questions |
| Sexual and Reproductive Health of key populations | 0 | + Add Questions |

In partnership with Canada | icddr,b

**Layout 16:** Count of Submitted RQs

# Appendix S2

# Online Data Collection Platform (Phase 2)

## Prioritisation of Research Questions for Advancing the Understanding of Sexual and Reproductive Health and Rights (SRHR) in Bangladesh

# PHASE 2

### Welcome to phase-2 of the AdSEARCH research prioritisation exercise on SRHR!!!!!!!

**About AdSEARCH**

The Department of Foreign Affairs, Trade and Development (DFADT) funded the Advancing Sexual and Reproductive Health and Rights (AdSEARCH) project of icddr,b which aims to improve sexual and reproductive health (SRH) outcomes and realise rights among different population groups with distinct SRH needs in Bangladesh.

**About AdSEARCH research priority setting exercise**

One of the objectives of AdSEARCH is to Prioritisation of Research Questions for Advancing the Understanding of Sexual and Reproductive Health and Rights (SRHR) in Bangladesh. AdSEARCH research prioritisation team has adopted the **Child Health and Nutrition Research Initiative (CHNRI)** method for the research prioritisation exercise involving technical experts and stakeholders.

**Your role**

You are approached to participate in the research prioritisation exercise as a **Technical Expert** based on your expertise and significant contribution in the field of SRHR (Adolescent Health/Fertility/Maternal and Neonatal Health/Gynecological issues/Sexual and reproductive health of key populations). The exercise requires your input in **two phases.**

- We have already completed the data collection of **first phase**
- In the **second phase**, we require you to score Research questions (collected in first phase) based on the theme(s) (Adolescent Health/Fertility/Maternal and Neonatal Health/Gynecological issues/Sexual and reproductive health of key populations) of interest. The second phase will take approximately 15-20 minutes. Your participation is completely voluntary, and you can withdraw your participation at any time during the process. Anonymity and confidentiality will be strictly maintained throughout the process. However, you will be acknowledged for your contribution in the planned scientific outputs of this research i.e., publications and reports (subject to your consent).

☐ **I agree to participate in phase 2 of the exercise after reading the consent form**

Register

**Privacy Policy**

For any query or assistance, please contact us at adsearch_rp@icddrb.org

In partnership with Canada icddr,b

**Layout 1:** Welcome Page of RP Research Prioritisation Exercise Phase 2

## Prioritisation of Research Questions for Advancing the Understanding of Sexual and Reproductive Health and Rights (SRHR) in Bangladesh

## PHASE 2

### Consent Form

**Title of the study:** Prioritisation of Research Questions for Advancing the Understanding of Sexual and Reproductive Health and Rights (SRHR) in Bangladesh

**Principal Investigator: Abu Sayeed**

**Research Organizations:** This research is part of the Advancing Sexual and Reproductive Health and Rights (AdSEARCH) research prioritisation exercise, conducted by International Centre for Diarrhoeal Disease Research, Bangladesh (icddr,b).

**Purpose of the research:**
Dear Sir/Mam,

We are contacting you from the International Centre for Diarrhoeal Disease Research, Bangladesh (icddr,b). We are conducting a research named **"Prioritisation of Research Questions for Advancing the Understanding of Sexual and Reproductive Health and Rights (SRHR) in Bangladesh"** to identify health research priorities related to SRHR in Bangladesh.

**Why have we invited you?**
Given your expertise in research related to SRHR (adolescent health / fertility/ maternal and neonatal health/ gynecological issues / sexual and reproductive health of key populations) and/or your publication(s) in this area, we are inviting you to kindly participate in this study. Your participation will help us by providing valuable expert information related to the SRHR of Bangladesh.

**Methods and Procedures:**
If you agree to participate in this study, we will ask you to submit one to five most promising research questions related to SRHR (adolescent health / fertility/ maternal and neonatal health/ gynecological issues and sexual health/ SRH of key populations) of Bangladesh. And you will be requested to submit your questions within four weeks. After that, we will send you **a scoring sheet with brief instruction and a set of collated research questions. We will ask you to provide your score according to the instructions.** It will take around thirty minutes for you to submit research questions and **during the scoring of the combined research questions it will take around five to eight minutes for each theme.**

**Risks and Benefits:**
There will be no or minimal risks related to your participation in this study. The information collected from you will be kept confidential and will only be used for research purpose. Participation in this study may not benefit you directly but the information you share will be very useful for improving SRHR in Bangladesh.

**Privacy, anonymity and confidentiality:**
All information collected from you during the study will be kept strictly confidential and not shared with anyone outside the study team. The data will be coded so that the personal identity and individual data from are traceable only with the code key which will be held by the study researchers, no one else will have access to it.

**Future use of information:**
Anonymous or abstracted information and data may be shared with other researchers. However, this will not conflict with or violate the maintenance of privacy, anonymity and confidentiality of information identifying participants in any way.

**Right not to participate and withdraw:**
You have the sole authority to decide about your own participation in this study. You would also be able to withdraw from participation any time during the study, without any explanation required.

**Principle of compensation:** Your participation in this study is completely voluntary and you will not get any payment for participating in this study.

**Contact person:**
If you have questions about this study or if you feel that you have been treated unfairly or have been affected in any way by joining the study, you may communicate with the Principal Investigator of the study Abu Sayeed, Maternal and Child Health Division, icddr,b by calling Mobile: +8801753837266 .

You can also contact icddr,b secretariat, Mr. M. A. Salam Khan (Phone: (+88 02) 9827084 or PABX (+88 02) 9827001-10, ext. 3206).

If you agree to our proposal of enrolling in our study, please indicate that by adding your signature in the specified space below.

Thank you for your consideration.

**Participant:** I certify that all the above information was adequately explained to me and I understand the explanation.

☐ **I agree to participate in phase 2 of the exercise**

Submit

In partnership with Canada icddr,b

**Layout 2:** Consent Form Page for Participation

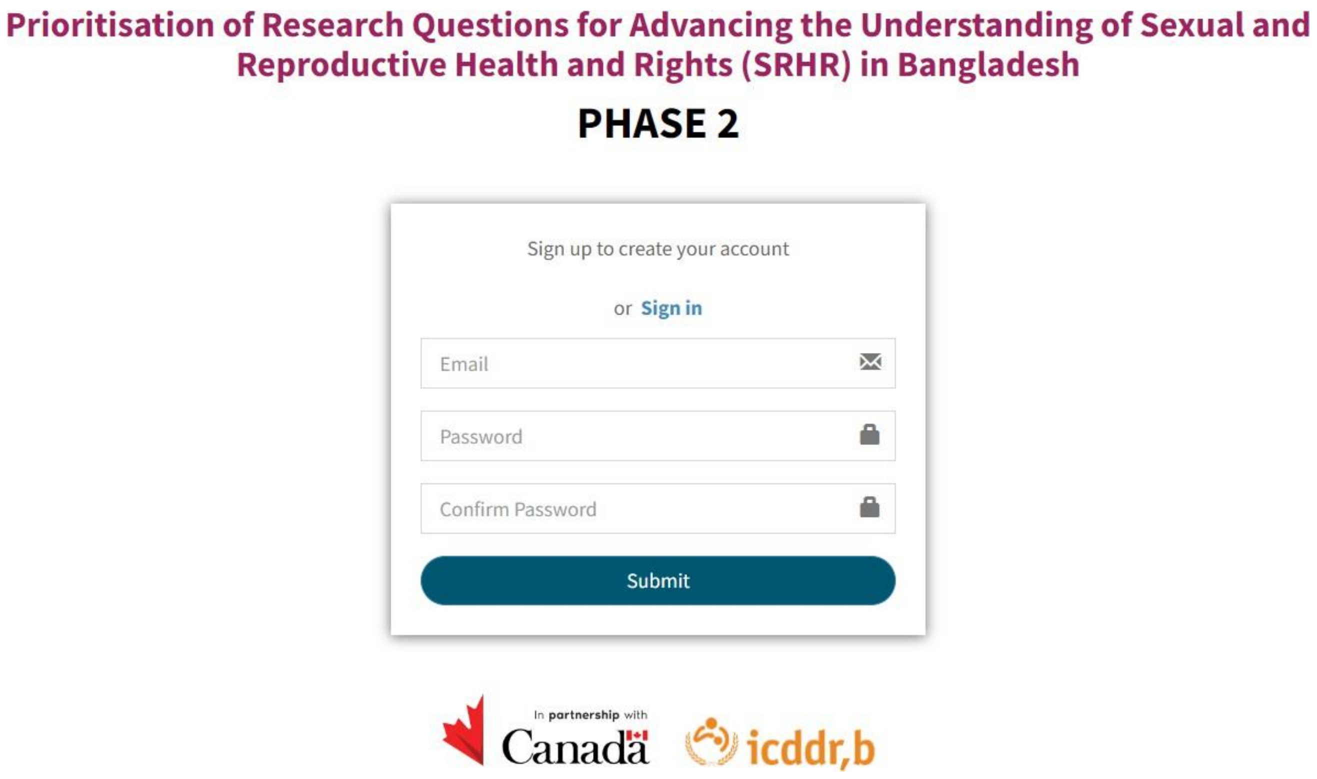


**Layout 3:** Online Registration Page (Blank)

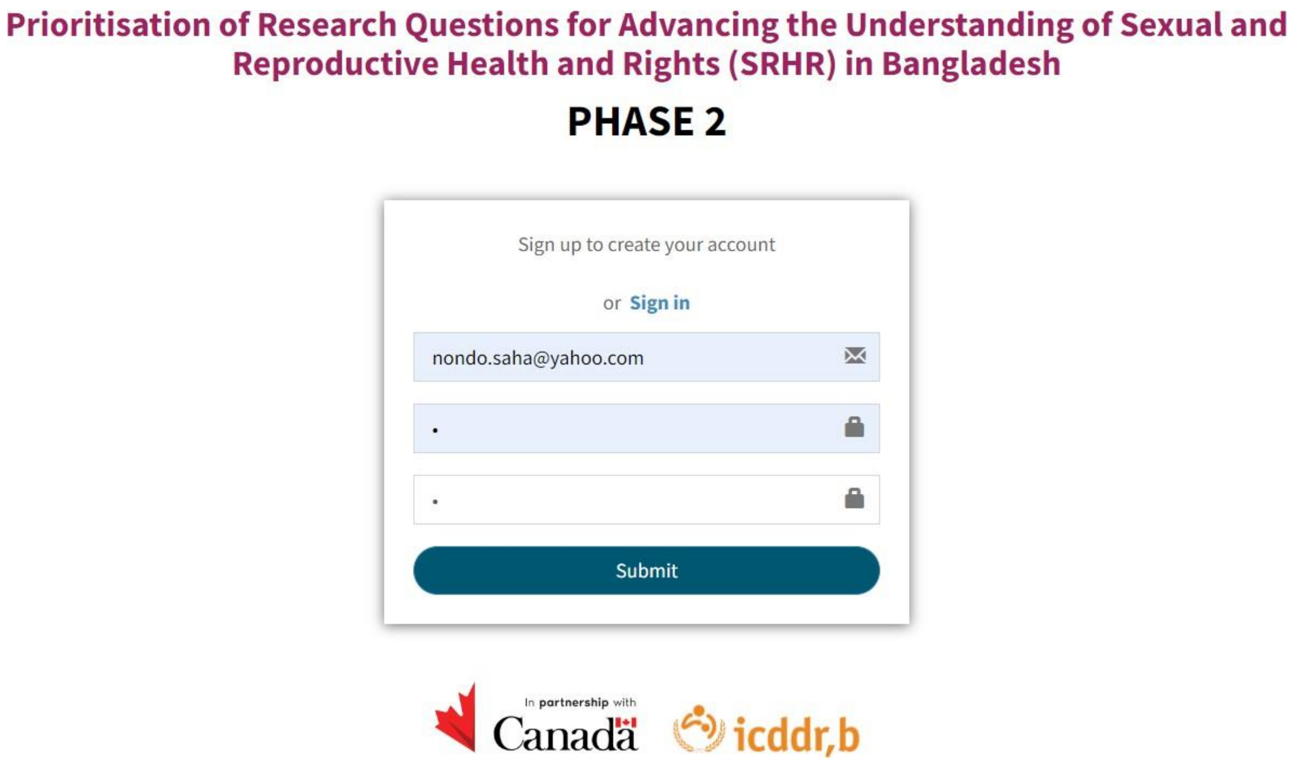


**Layout 4:** Online Registration Page (Filled)

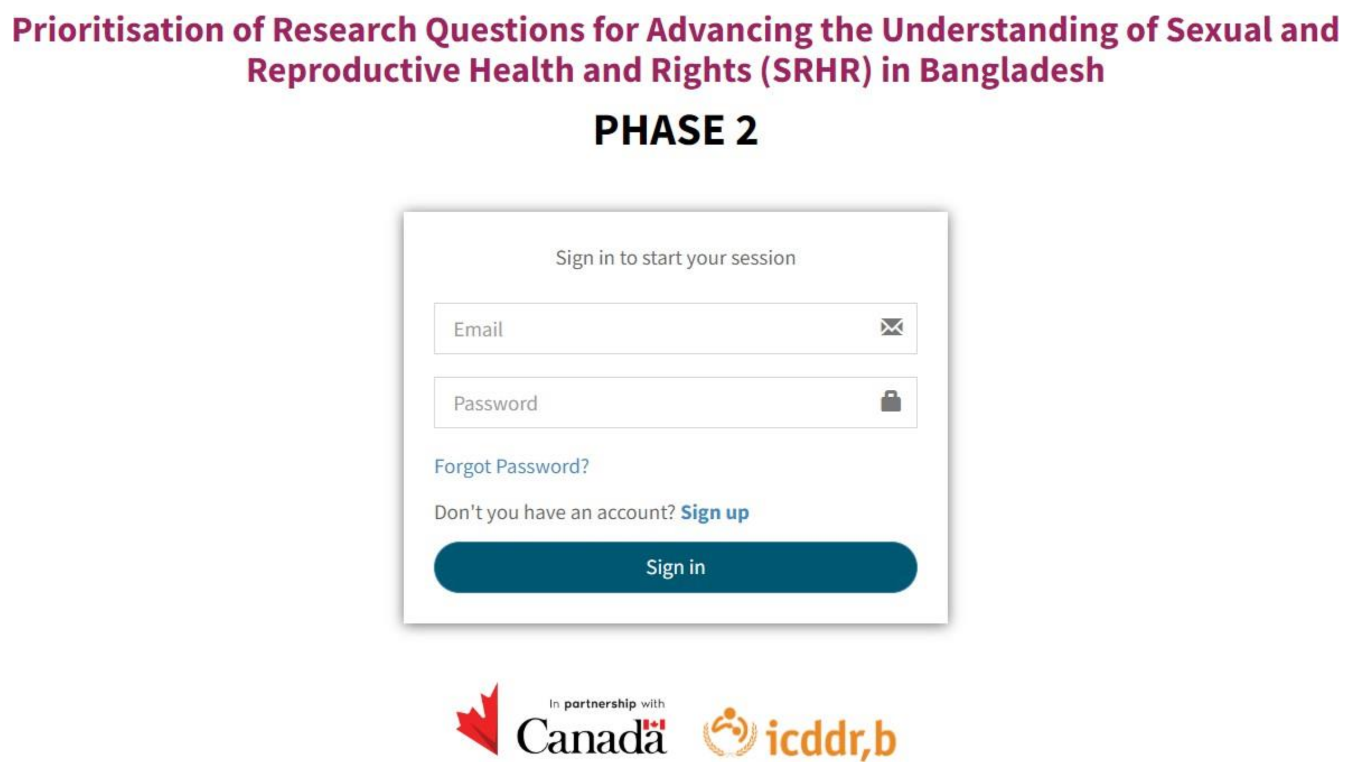


**Layout 5:** Online Log in Page (Blank)

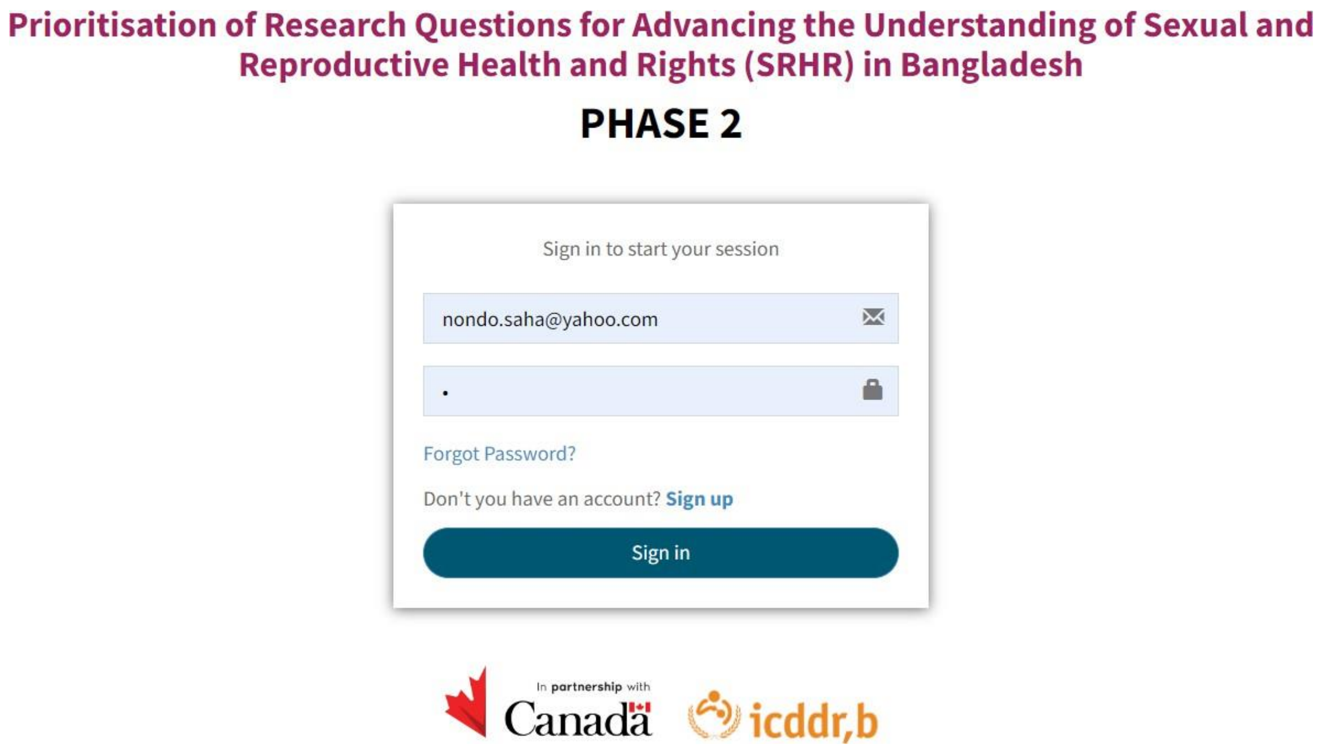


**Layout 6:** Online Log in Page (Filled)

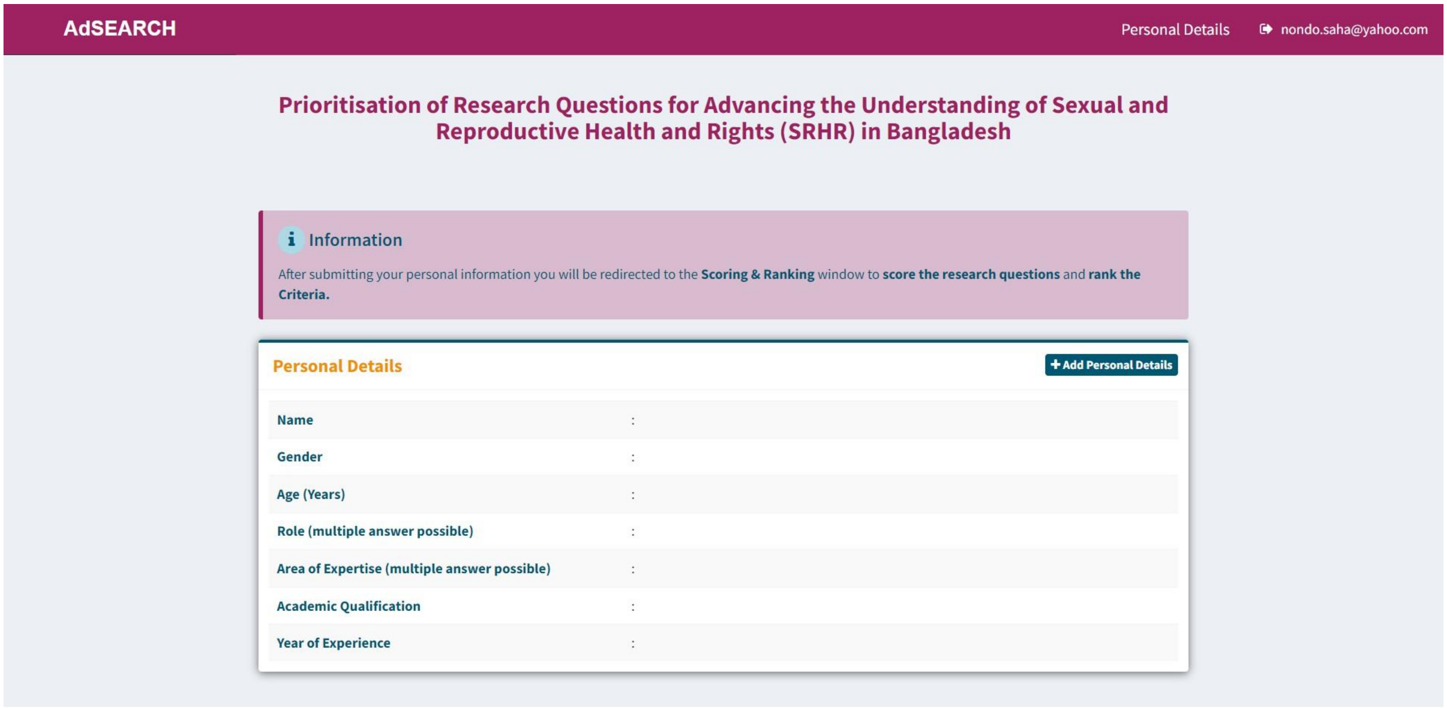


**Layout 7:** Personal details Page (Blank)

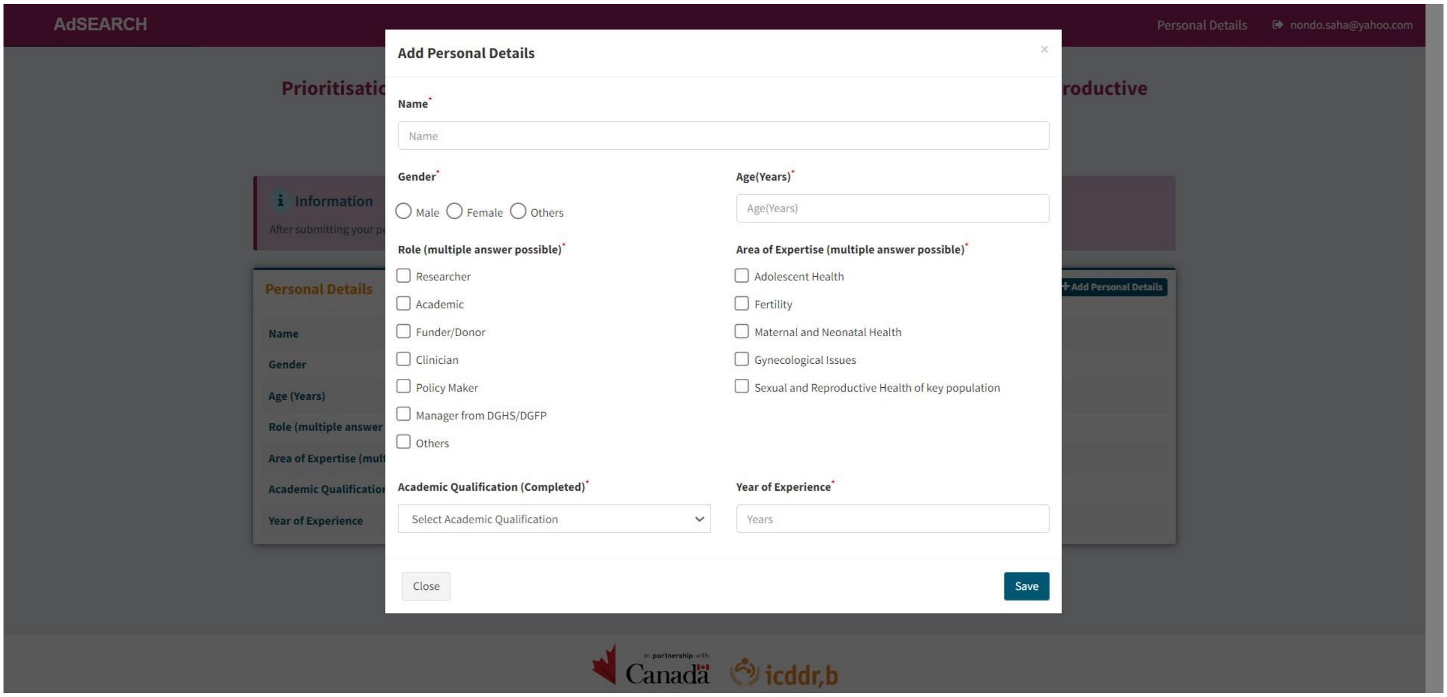


**Layout 8:** Pop UP Personal details Page (Blank)

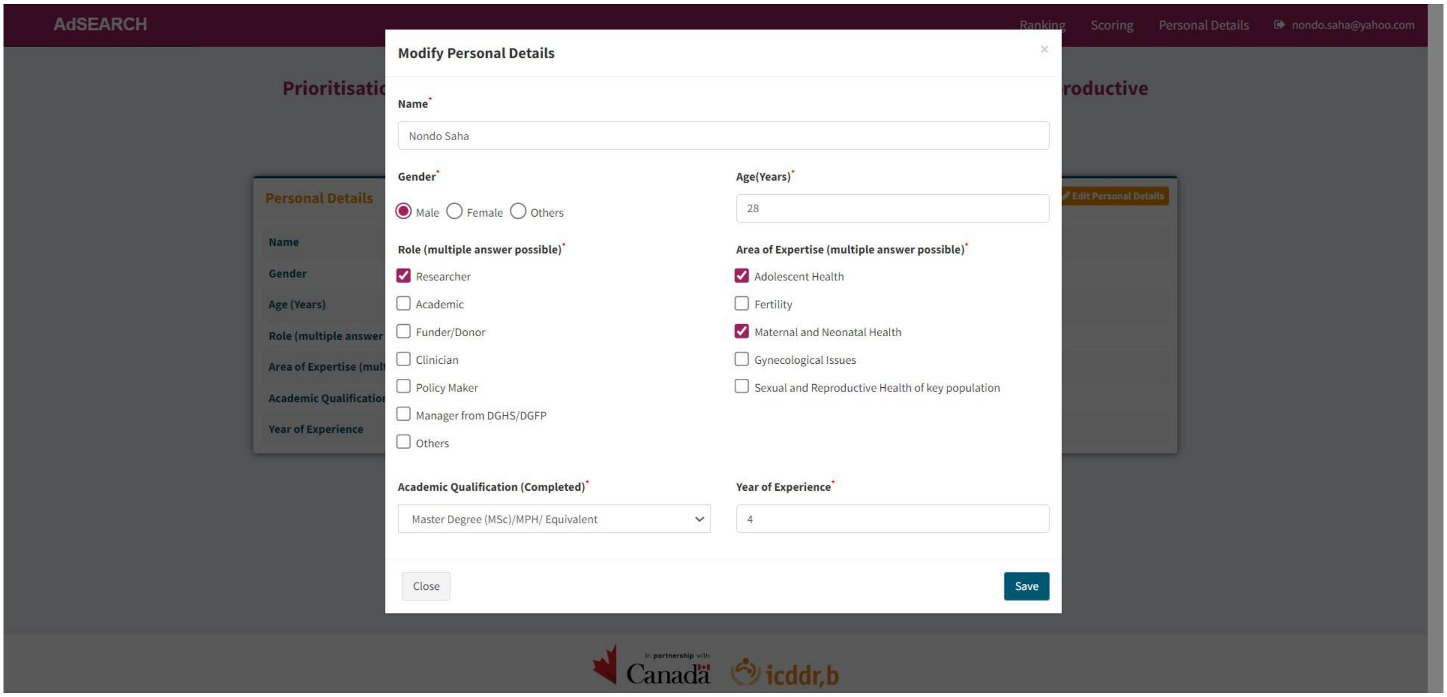


**Layout 9:** Pop UP Personal details Page (Filled)

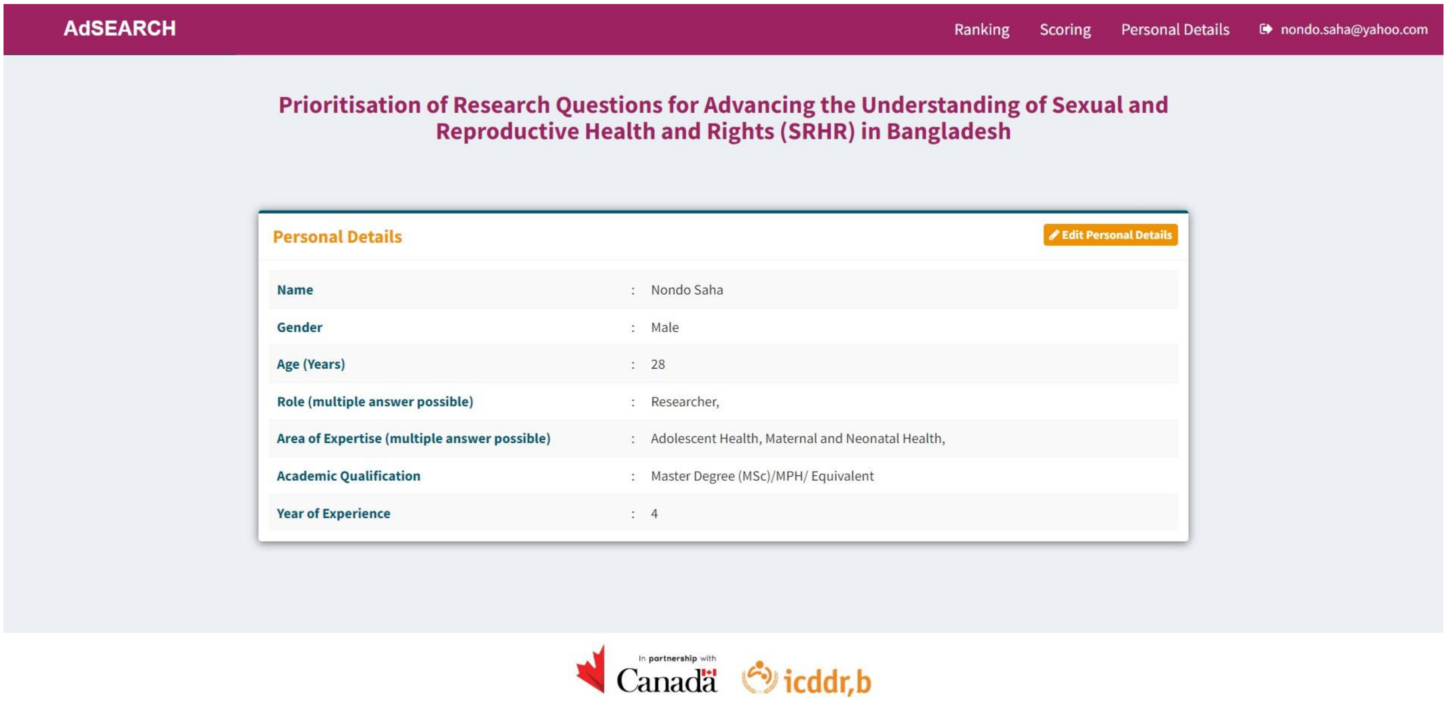


**Layout 10:** Personal details Page (Filled)

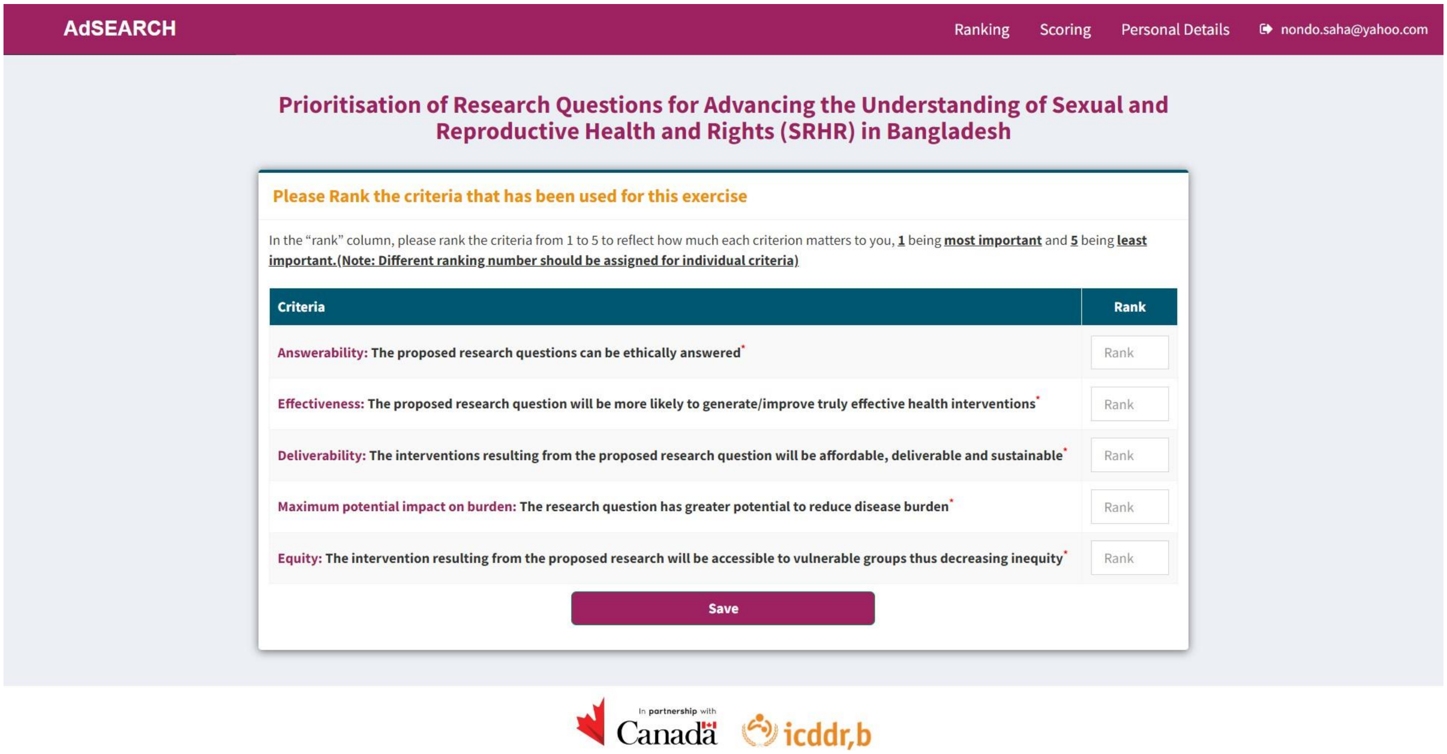


**Layout 11:** Criteria Ranking Page (Blank)

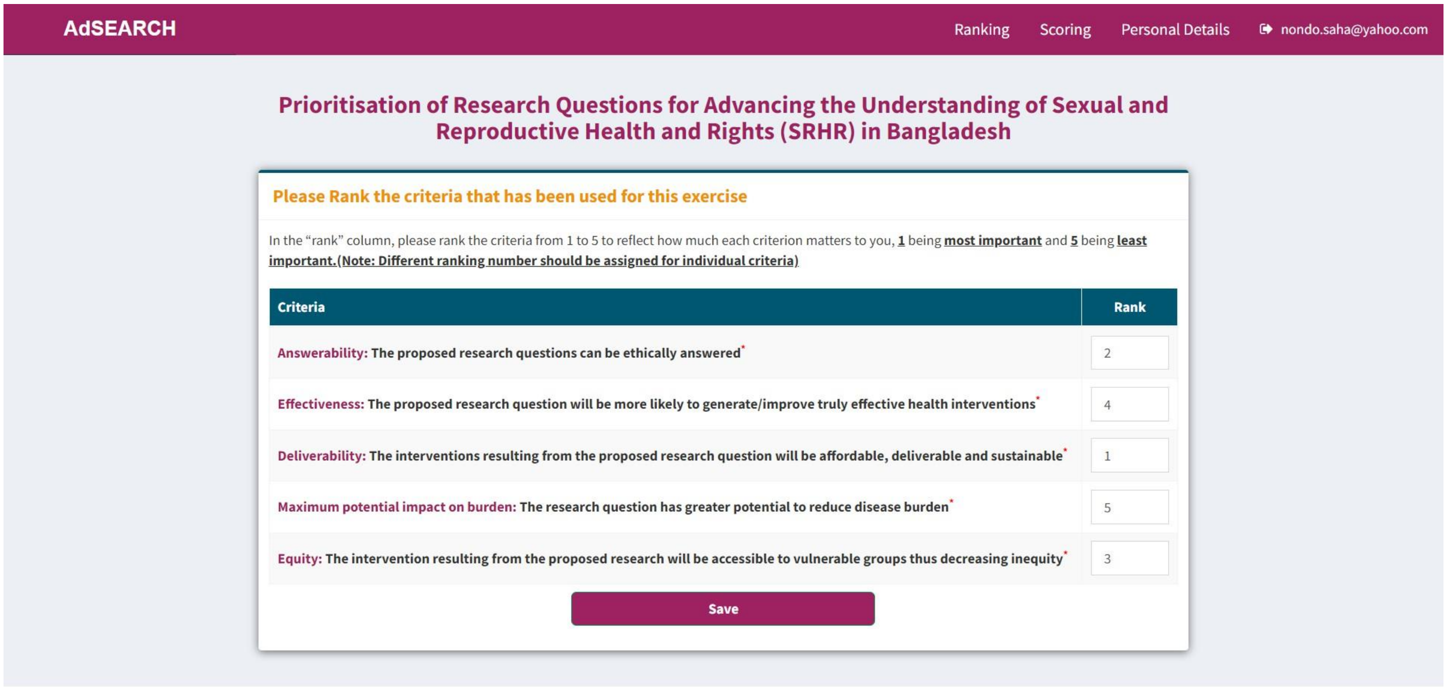
AdSEARCH

Ranking Scoring Personal Details nondo.saha@yahoo.com

**Prioritisation of Research Questions for Advancing the Understanding of Sexual and Reproductive Health and Rights (SRHR) in Bangladesh**

**Please Rank the criteria that has been used for this exercise**

In the "rank" column, please rank the criteria from 1 to 5 to reflect how much each criterion matters to you, **1** being **most important** and **5** being **least important.(Note: Different ranking number should be assigned for individual criteria)**

| Criteria | Rank |
| --- | --- |
| **Answerability:** The proposed research questions can be ethically answered* | 2 |
| **Effectiveness:** The proposed research question will be more likely to generate/improve truly effective health interventions* | 4 |
| **Deliverability:** The interventions resulting from the proposed research question will be affordable, deliverable and sustainable* | 1 |
| **Maximum potential impact on burden:** The research question has greater potential to reduce disease burden* | 5 |
| **Equity:** The intervention resulting from the proposed research will be accessible to vulnerable groups thus decreasing inequity* | 3 |

Save

In partnership with Canada icddr,b

**Layout 12:** Criteria Ranking Page (Filled)

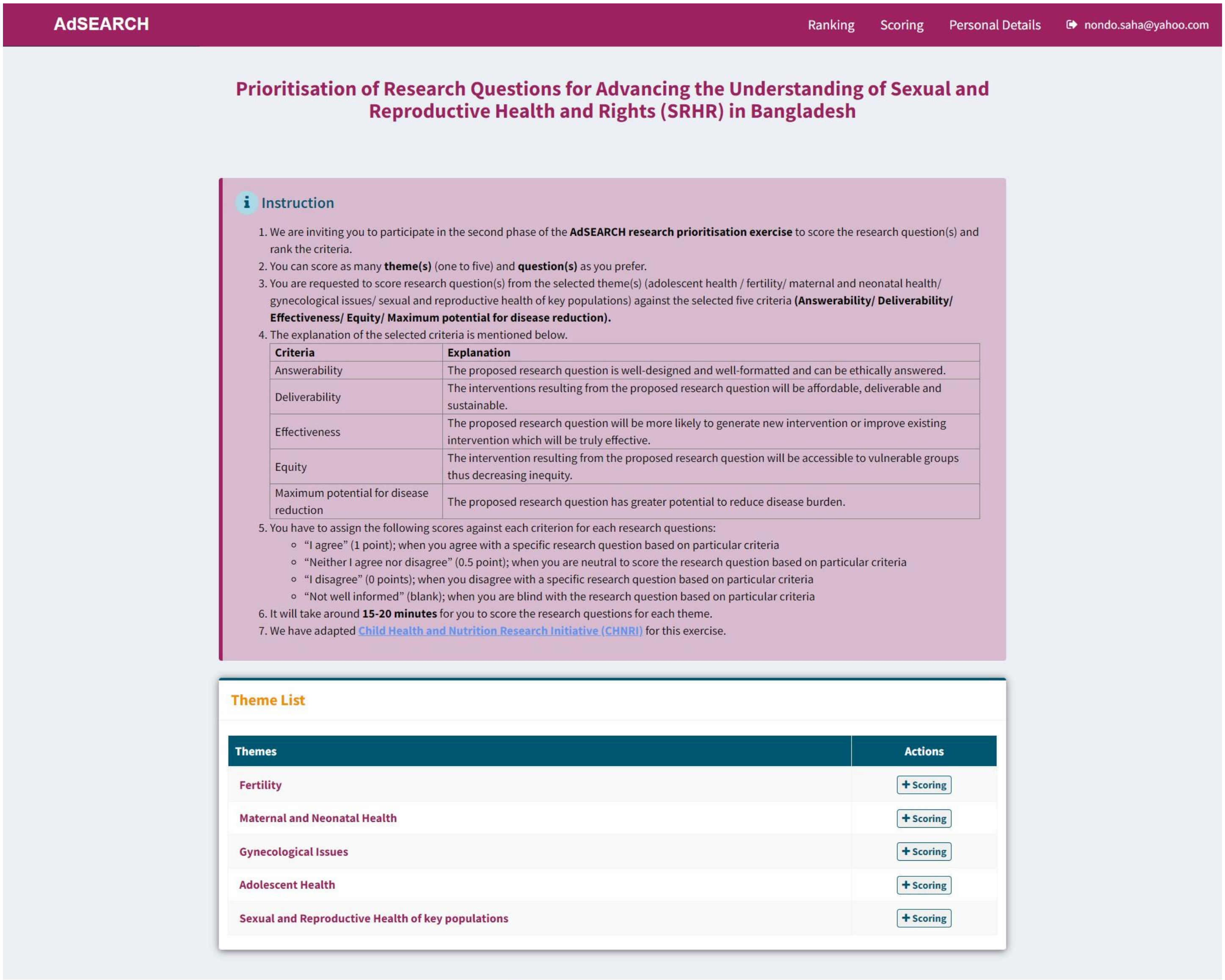

AdSEARCH　Ranking　Scoring　Personal Details　nondo.saha@yahoo.com

## Prioritisation of Research Questions for Advancing the Understanding of Sexual and Reproductive Health and Rights (SRHR) in Bangladesh

### Instruction

1. We are inviting you to participate in the second phase of the **AdSEARCH research prioritisation exercise** to score the research question(s) and rank the criteria.
2. You can score as many **theme(s)** (one to five) and **question(s)** as you prefer.
3. You are requested to score research question(s) from the selected theme(s) (adolescent health / fertility/ maternal and neonatal health/ gynecological issues/ sexual and reproductive health of key populations) against the selected five criteria **(Answerability/ Deliverability/ Effectiveness/ Equity/ Maximum potential for disease reduction).**
4. The explanation of the selected criteria is mentioned below.

| Criteria | Explanation |
|---|---|
| Answerability | The proposed research question is well-designed and well-formatted and can be ethically answered. |
| Deliverability | The interventions resulting from the proposed research question will be affordable, deliverable and sustainable. |
| Effectiveness | The proposed research question will be more likely to generate new intervention or improve existing intervention which will be truly effective. |
| Equity | The intervention resulting from the proposed research question will be accessible to vulnerable groups thus decreasing inequity. |
| Maximum potential for disease reduction | The proposed research question has greater potential to reduce disease burden. |

5. You have to assign the following scores against each criterion for each research questions:
   - "I agree" (1 point); when you agree with a specific research question based on particular criteria
   - "Neither I agree nor disagree" (0.5 point); when you are neutral to score the research question based on particular criteria
   - "I disagree" (0 points); when you disagree with a specific research question based on particular criteria
   - "Not well informed" (blank); when you are blind with the research question based on particular criteria
6. It will take around **15-20 minutes** for you to score the research questions for each theme.
7. We have adapted Child Health and Nutrition Research Initiative (CHNRI) for this exercise.

### Theme List

| Themes | Actions |
|---|---|
| Fertility | + Scoring |
| Maternal and Neonatal Health | + Scoring |
| Gynecological Issues | + Scoring |
| Adolescent Health | + Scoring |
| Sexual and Reproductive Health of key populations | + Scoring |

In partnership with Canada　icddr,b

**Layout 13:** Instruction for Scoring RQs

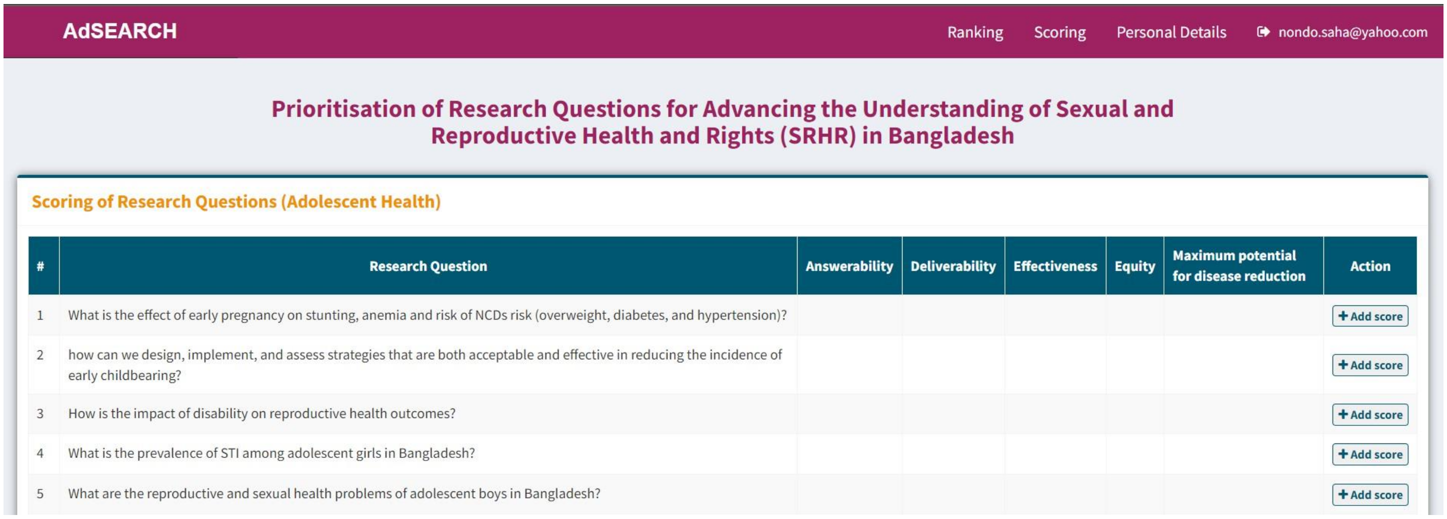


**Layout 14:** RQs Scoring Page (Blank)

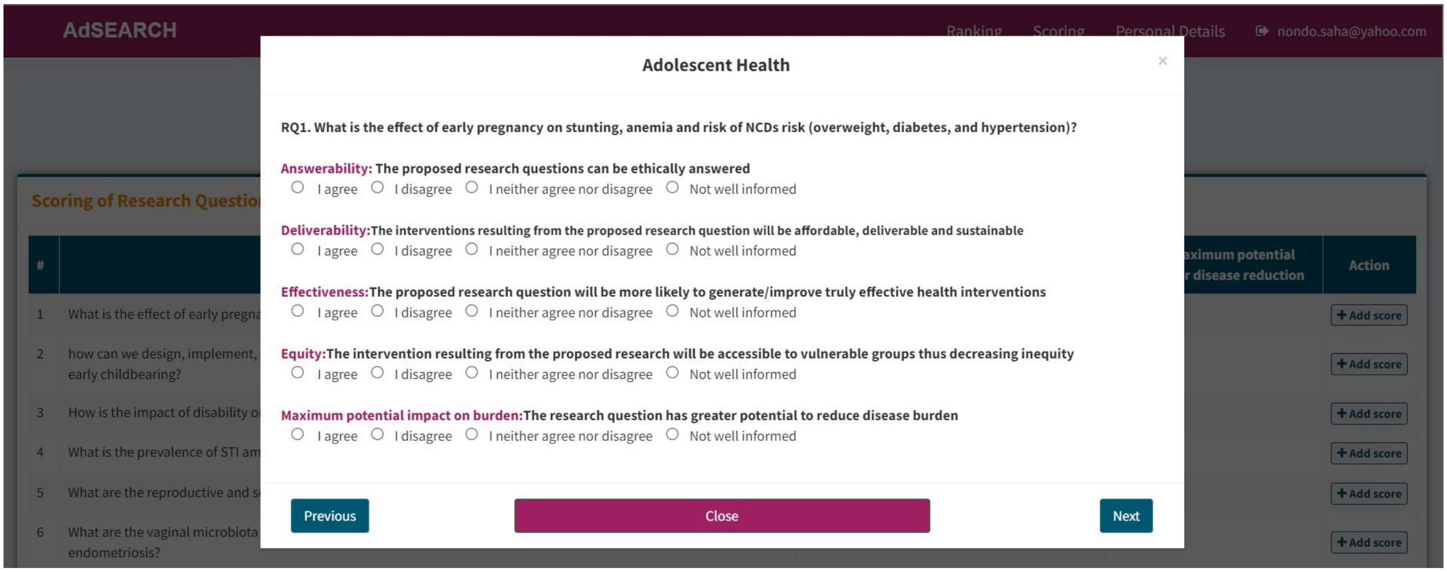


**Layout 14:** Pop Up RQs Scoring Page (Blank)

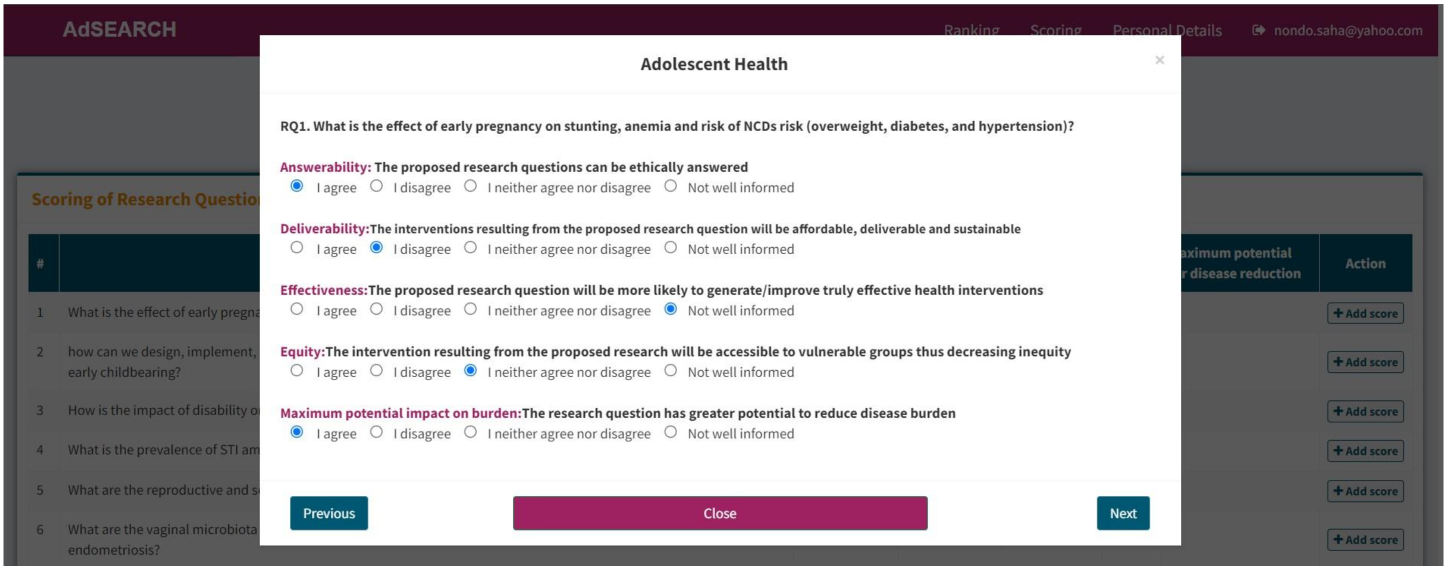


**Layout 16:** Pop Up RQs Scoring Page (Filled)

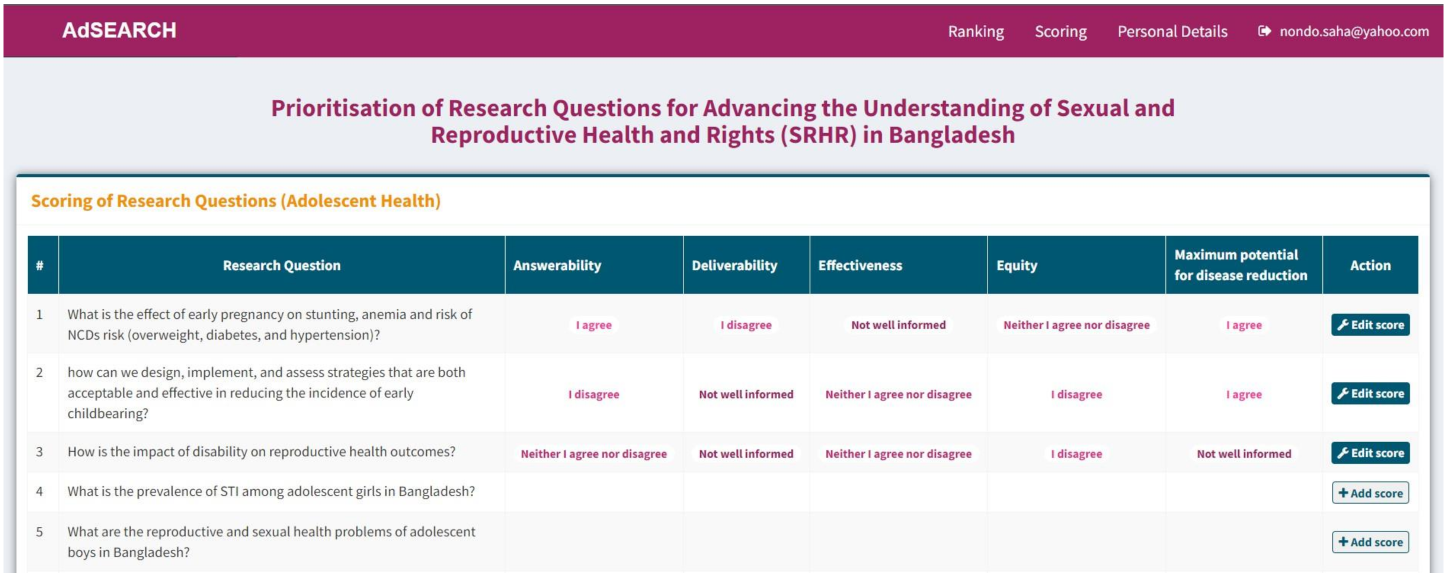

AdSEARCH

Ranking Scoring Personal Details nondo.saha@yahoo.com

## Prioritisation of Research Questions for Advancing the Understanding of Sexual and Reproductive Health and Rights (SRHR) in Bangladesh

### Scoring of Research Questions (Adolescent Health)

| # | Research Question | Answerability | Deliverability | Effectiveness | Equity | Maximum potential for disease reduction | Action |
|---|---|---|---|---|---|---|---|
| 1 | What is the effect of early pregnancy on stunting, anemia and risk of NCDs risk (overweight, diabetes, and hypertension)? | I agree | I disagree | Not well informed | Neither I agree nor disagree | I agree | Edit score |
| 2 | how can we design, implement, and assess strategies that are both acceptable and effective in reducing the incidence of early childbearing? | I disagree | Not well informed | Neither I agree nor disagree | I disagree | I agree | Edit score |
| 3 | How is the impact of disability on reproductive health outcomes? | Neither I agree nor disagree | Not well informed | Neither I agree nor disagree | I disagree | Not well informed | Edit score |
| 4 | What is the prevalence of STI among adolescent girls in Bangladesh? | | | | | | + Add score |
| 5 | What are the reproductive and sexual health problems of adolescent boys in Bangladesh? | | | | | | + Add score |

**Layout 17:** RQs Scoring Page (Filled)

**Table S2.** All the 197 Research questions with domain, theme, scores for each criterion, weighted RPS, and AEA

| Question No. | Rank | Research Question | Domain | Theme | Answerability iRPS | Deliverability iRPS | Effectiveness iRPS | Equity iRPS | Maximum impact on burden iRPS | Weighted RPS | AEA (Excluding NWI) |
|---|---|---|---|---|---|---|---|---|---|---|---|
| Q193 | 1 | How can stigma and discrimination against Key Populations be reduced to improve their sexual and reproductive health? | Discovery | SRHKP | 0.975 | 1.010 | 1.170 | 0.776 | 0.872 | 0.945 | 0.906 |
| Q151 | 2 | What barriers hinder implementing Newborn Stabilizing Units in upazila hospitals? | Delivery | MNH | 0.995 | 0.989 | 1.133 | 0.783 | 0.836 | 0.932 | 0.891 |
| Q169 | 3 | What are the obstacles in raising awareness about the Sexual and Reproductive Health Rights (SRHR) of Key Populations (KPs) in Bangladesh? | Delivery | SRHKP | 0.996 | 0.985 | 1.096 | 0.757 | 0.873 | 0.926 | 0.882 |
| Q152 | 4 | What is the impact of the Postpartum Haemorrhage (PPH) bundle approach on the management of PPH cases? | Description | MNH | 0.985 | 1.006 | 1.099 | 0.756 | 0.854 | 0.925 | 0.890 |
| Q033 | 5 | How is adolescent pregnancy associated with child undernutrition, maternal anemia, and the risk of NCDs such as diabetes, and hypertension of the mother? | Description | AH | 1.003 | 1.003 | 1.123 | 0.685 | 0.886 | 0.925 | 0.869 |
| Q122 | 6 | Does the enhancement of counseling on danger signs during antenatal care (ANC) contribute to a significant reduction in adverse birth outcomes? | Description | MNH | 0.966 | 1.008 | 1.112 | 0.766 | 0.846 | 0.925 | 0.895 |
| Q055 | 7 | What key factors hinder or support birth spacing strategies for newlywed and underage married women, and how can they be integrated into healthcare systems? | Delivery | F | 0.972 | 0.965 | 1.153 | 0.786 | 0.820 | 0.924 | 0.885 |
| Q141 | 8 | What is the burden and risk factors for mental health issues among pregnant women? | Description | MNH | 0.994 | 0.968 | 1.119 | 0.743 | 0.869 | 0.924 | 0.893 |
| Q132 | 9 | What are the bottlenecks, barriers, and challenges in identifying and managing high-risk pregnancies in low- and middle-income countries like Bangladesh? | Delivery | MNH | 0.985 | 0.976 | 1.124 | 0.747 | 0.854 | 0.922 | 0.868 |
| Q008 | 10 | What is the impact of school-based comprehensive sexuality education given to young adolescents on prevention of gender-based violence, unsafe abortion, and smooth transitioning to adolescence from childhood? | Description | AH | 0.959 | 0.980 | 1.125 | 0.755 | 0.855 | 0.920 | 0.872 |
| Q020 | 11 | What are the barriers faced by health care provider to provide mental health services among adolescents? | Delivery | AH | 0.965 | 0.993 | 1.122 | 0.752 | 0.836 | 0.919 | 0.861 |
| Q031 | 12 | What is the impact of adolescent pregnancy on maternal mortality/morbidity? | Description | AH | 0.972 | 0.991 | 1.117 | 0.759 | 0.820 | 0.917 | 0.854 |
| Q103 | 13 | What are the challenges, key factors, and enablers of implementing HPV testing for cervical cancer screening? | Delivery | GI | 0.981 | 0.955 | 1.138 | 0.751 | 0.824 | 0.915 | 0.878 |
| Q183 | 14 | What strategies need to be implemented to get better sexual and reproductive health & family planning service from government health system by key populations (KP)? | Discovery | SRHKP | 0.945 | 0.982 | 1.112 | 0.735 | 0.872 | 0.914 | 0.870 |
| Q163 | 15 | What are the most effective interventions for the prevention and treatment of low birth weight infants? | Description | MNH | 0.964 | 0.973 | 1.119 | 0.745 | 0.834 | 0.912 | 0.867 |
| Q098 | 16 | What strategies can be implemented to scaling up the early detection of gynecological cancers in Bangladesh? | Discovery | GI | 0.959 | 0.993 | 1.125 | 0.731 | 0.826 | 0.912 | 0.888 |
| Q007 | 17 | What is the impact of introducing life skills lessons in the school educational curriculum on empowerment of adolescent girls and prevention of child marriage? | Description | AH | 0.992 | 0.983 | 1.100 | 0.720 | 0.826 | 0.910 | 0.861 |
| Q024 | 18 | What is the impact of health education on the preference of using menstrual kit among adolescent girl? | Description | AH | 0.993 | 1.000 | 1.142 | 0.696 | 0.781 | 0.908 | 0.841 |
| Q192 | 19 | How do stigma and discriminatory practices impact the sexual and reproductive health behaviors of Key Populations? | Description | SRHKP | 0.972 | 0.973 | 1.061 | 0.747 | 0.843 | 0.905 | 0.842 |

| | | | | | | | | | | | |
|---|---|---|---|---|---|---|---|---|---|---|---|
| Q170 | 20 | What challenges do Key Populations encounter in the diagnosis of COVID-19, treatment when COVID-19 positive, and the administration of vaccination against COVID-19? | Delivery | SRHKP | 0.983 | 0.922 | 1.121 | 0.761 | 0.807 | 0.904 | 0.848 |
| Q197 | 21 | What is the current situation regarding the Sexual and Reproductive Health and Rights (SRHR) of Key Populations (KPs) in Bangladesh and the discrepancy between policy and the grounded reality? | Description | SRHKP | 0.963 | 0.959 | 1.094 | 0.726 | 0.851 | 0.904 | 0.859 |
| Q147 | 22 | What are the current availability, quality, and accessibility of maternal and newborn health (MNH) services in hard-to-reach areas? | Description | MNH | 0.986 | 0.939 | 1.078 | 0.735 | 0.854 | 0.904 | 0.874 |
| Q196 | 23 | What types of interventions and/or new technologies can be used to help key populations overcome the obstacles that threaten their sexual and reproductive health right (SRHR) outcomes? | Discovery | SRHKP | 0.971 | 1.005 | 1.061 | 0.696 | 0.849 | 0.902 | 0.835 |
| Q195 | 24 | To what extent do key populations face socio-structural challenges that put their sexual and reproductive health right (SRHR) outcomes in jeopardy? | Description | SRHKP | 0.954 | 0.991 | 1.058 | 0.754 | 0.823 | 0.901 | 0.833 |
| Q021 | 25 | What are the effects of unwanted pregnancy on peripartum depression among adolescent? | Description | AH | 0.952 | 0.968 | 1.097 | 0.693 | 0.860 | 0.899 | 0.833 |
| Q124 | 26 | What is the status, impacts, and barriers of antenatal and postnatal care seeking of newborn both at facility and community? | Delivery | MNH | 0.962 | 0.965 | 1.092 | 0.737 | 0.814 | 0.899 | 0.846 |
| Q027 | 27 | How does the quality and accessibility of healthcare services for adolescents impact their knowledge and decision-making skills related to health? | Description | AH | 0.991 | 0.908 | 1.076 | 0.738 | 0.854 | 0.899 | 0.837 |
| Q121 | 28 | How does the implementation of depression screening tools in antenatal and postnatal care affect the early detection of peripartum depression? | Description | MNH | 0.952 | 0.956 | 1.113 | 0.723 | 0.822 | 0.899 | 0.845 |
| Q117 | 29 | What is the impact of school or academic institution's support on adolescents in dealing with their gynecological concerns? | Description | GI | 0.947 | 0.983 | 1.071 | 0.729 | 0.830 | 0.897 | 0.855 |
| Q168 | 30 | What strategy can be adapted to improve newborn interventions (ENC, KMC, Newborn Signal Functions etc.) for providing better support for premature and low birth weight newborns across all levels of healthcare facilities? | Discovery | MNH | 0.947 | 0.954 | 1.075 | 0.749 | 0.828 | 0.896 | 0.833 |
| Q032 | 31 | What is the most acceptable and effective strategy for educating young adolescents about adverse effects of self-termination of pregnancy's using traditional medicine? | Development | AH | 0.935 | 0.958 | 1.071 | 0.734 | 0.855 | 0.896 | 0.817 |
| Q088 | 32 | What is the knowledge, attitude and practice of menstrual regulation among reproductive aged women in Bangladesh? | Description | F | 0.991 | 0.952 | 1.068 | 0.714 | 0.824 | 0.895 | 0.831 |
| Q158 | 33 | What are the barriers to implement effective maternal health referral systems in hard-to-reach areas? | Delivery | MNH | 0.985 | 0.925 | 1.087 | 0.719 | 0.828 | 0.894 | 0.842 |
| Q157 | 34 | What is the burden and risk factors for pregnancy loss in Bangladesh? | Description | MNH | 0.964 | 0.929 | 1.097 | 0.702 | 0.852 | 0.894 | 0.811 |
| Q120 | 35 | What interventions can be adapted to improve maternal health counselling during periconceptional, ANC & PNC period? | Development | MNH | 0.936 | 0.976 | 1.111 | 0.713 | 0.802 | 0.893 | 0.816 |
| Q018 | 36 | What psychosocial interventions are available to support adolescent's wellbeing and mental health in Bangladesh? | Description | AH | 0.962 | 0.953 | 1.082 | 0.714 | 0.823 | 0.892 | 0.834 |
| Q143 | 37 | What is the impact of implementing telehealth/mHealth technology by community health workers during home visits on neonatal survival? | Description | MNH | 0.983 | 0.933 | 1.070 | 0.737 | 0.805 | 0.891 | 0.811 |
| Q179 | 38 | What strategies and interventions can be adapted to improve access to and uptake of HIV testing services among Key Populations (KPs)? | Discovery | SRHKP | 0.956 | 0.952 | 1.080 | 0.711 | 0.820 | 0.889 | 0.817 |

| | | | | | | | | | | | |
|---|---|---|---|---|---|---|---|---|---|---|---|
| Q150 | 39 | What is the impact of hypothermia prevention bundle care to prevent neonatal morbidity and mortality among facility delivered newborn? | Description | MNH | 0.952 | 0.956 | 1.082 | 0.696 | 0.831 | 0.889 | 0.817 |
| Q186 | 40 | What strategy can be adapted to increase the access of socially neglected lesbian, gay, bisexual, transgender, queer or questioning (LGBTQ) individuals towards SRH services? | Discovery | SRHKP | 0.928 | 0.946 | 1.100 | 0.717 | 0.818 | 0.887 | 0.821 |
| Q090 | 41 | What is the cost-effectiveness of different gynecological cancer screening approaches in Bangladesh considering the local health system and socio-economic context? | Description | GI | 0.953 | 0.961 | 1.105 | 0.694 | 0.796 | 0.887 | 0.821 |
| Q019 | 42 | What are the barriers to implementing adolescent-friendly mental health services at primary health care level? | Delivery | AH | 0.966 | 0.951 | 1.078 | 0.713 | 0.800 | 0.887 | 0.836 |
| Q182 | 43 | How can structural interventions be utilised to promote a gender-inclusive sexual and reproductive health right (SRHR) intervention agenda for key populations (KPs)? | Development | SRHKP | 0.919 | 0.882 | 1.131 | 0.743 | 0.823 | 0.885 | 0.813 |
| Q176 | 44 | What strategies or interventions can be employed to support pregnant adolescents living with HIV and to improve both maternal and child health outcomes? | Discovery | SRHKP | 0.933 | 0.976 | 1.066 | 0.705 | 0.818 | 0.885 | 0.839 |
| Q009 | 45 | Does school-based comprehensive health and nutrition education program improve sexual and reproductive health, mental health, hygiene practice, and dietary diversity among adolescents? | Development | AH | 0.919 | 0.953 | 1.064 | 0.717 | 0.839 | 0.884 | 0.862 |
| Q110 | 46 | What effects do free or low-cost menstrual supplies have on the health of women who experience menstruation? | Description | GI | 0.993 | 0.906 | 1.087 | 0.723 | 0.781 | 0.884 | 0.829 |
| Q081 | 47 | What would be the most cost-effective, affordable, and feasible package of interventions for influencing women's access to sexual and reproductive health services? | Development | F | 0.969 | 0.958 | 1.071 | 0.698 | 0.783 | 0.882 | 0.804 |
| Q135 | 48 | What is the level of knowledge, attitude, practice, challenges, and barriers of health care providers (Doctors, Nurse, Midwives) regarding establishing, practicing newborn interventions (Essential Newborn Care, Kangaroo Mother Care, Newborn Signal Functions etc.)? | Description | MNH | 0.976 | 0.918 | 1.066 | 0.711 | 0.809 | 0.882 | 0.828 |
| Q123 | 49 | What strategies can be implemented at community and facility level to increase at least four ANC with medically providers in low-resource settings? | Discovery | MNH | 0.952 | 0.956 | 1.045 | 0.711 | 0.814 | 0.881 | 0.820 |
| Q078 | 50 | What strategies can be implemented to make infertility services more available, accessible, and affordable for marginalized populations? | Discovery | F | 0.939 | 0.956 | 1.092 | 0.696 | 0.793 | 0.881 | 0.824 |
| Q174 | 51 | What extent do health care providers encounter difficulties or roadblocks when attending to Key Populations (KPs) sexual and reproductive health (SRH) needs? | Description | SRHKP | 0.985 | 0.932 | 1.063 | 0.702 | 0.793 | 0.881 | 0.790 |
| Q029 | 52 | What is the effect of abuse (physical violence, sexual harassment and social bullies) on early marriage? | Description | AH | 0.913 | 0.926 | 1.051 | 0.741 | 0.842 | 0.880 | 0.810 |
| Q082 | 53 | What is the role of gender in fertility preferences and practices, and how does it influence access to sexual and reproductive health services for women? | Description | F | 0.963 | 0.929 | 1.070 | 0.699 | 0.801 | 0.878 | 0.796 |
| Q017 | 54 | What is the prevalence and risk factors of different mental health conditions e.g., Common Mental Disorders (CMDs), Severe Mental Disorders (SMDs) among adolescents in Bangladesh? | Description | AH | 0.972 | 0.887 | 1.064 | 0.696 | 0.841 | 0.878 | 0.807 |
| Q165 | 55 | How the health of newborn babies affected by their mothers' exposure to second-hand smoke during pregnancy? | Description | MNH | 0.964 | 0.945 | 1.048 | 0.693 | 0.807 | 0.877 | 0.822 |
| Q134 | 56 | What is the long-term health outcome of children who received Kangaroo Mother Care? | Description | MNH | 0.938 | 0.939 | 1.054 | 0.719 | 0.793 | 0.874 | 0.824 |
| Q059 | 57 | What are the most effective strategies to increase access to contraception in low resource settings like Bangladesh? | Development | F | 0.940 | 0.929 | 1.092 | 0.681 | 0.798 | 0.874 | 0.810 |

| | | | | | | | | | | | |
|---|---|---|---|---|---|---|---|---|---|---|---|
| Q194 | 58 | What forms of discrimination exist in providing sexual and reproductive health right (SRHR) services to Key Populations (KPs)? | Description | SRHKP | 0.955 | 0.922 | 1.063 | 0.671 | 0.827 | 0.873 | 0.822 |
| Q156 | 59 | What is the burden and causes of different types of high-risk pregnancy in Bangladesh? | Description | MNH | 0.950 | 0.938 | 1.051 | 0.669 | 0.830 | 0.873 | 0.829 |
| Q091 | 60 | What is the impact of a cancer screening registry (collects, utilizes, and stores cancer screening data on individuals) on program management and reporting? | Description | GI | 0.918 | 0.931 | 1.100 | 0.704 | 0.780 | 0.872 | 0.807 |
| Q048 | 61 | Is the micronutrient deficiency (Vitamin B12 and folic acid) among the adolescent pregnant women associated with adverse pregnancy outcomes? | Description | AH | 0.945 | 0.955 | 1.044 | 0.695 | 0.786 | 0.871 | 0.793 |
| Q010 | 62 | How can sexual and reproductive health (SRH) issues be incorporated in the national education curriculum? | Delivery | AH | 0.928 | 0.946 | 1.064 | 0.720 | 0.766 | 0.871 | 0.804 |
| Q026 | 63 | What is the sexual and reproductive health (SRH) knowledge status of adolescent boys and girls at district level in Bangladesh? | Description | AH | 0.939 | 0.958 | 1.048 | 0.698 | 0.776 | 0.870 | 0.798 |
| Q136 | 64 | What are the best possible way to improve the measurement of acute malnutrition in pregnant women? | Development | MNH | 0.955 | 0.918 | 1.075 | 0.697 | 0.773 | 0.869 | 0.794 |
| Q085 | 65 | What are the impacts of measurement of the quality index indicators (good counselling, privacy and confidentiality during service provision, infection control and waste management) on maintaining quality of PPFP services? | Description | F | 0.953 | 0.944 | 1.073 | 0.673 | 0.774 | 0.869 | 0.795 |
| Q131 | 66 | What are the short- and long-term maternal and child health consequences of c-section? | Discovery | MNH | 0.968 | 0.922 | 1.052 | 0.673 | 0.800 | 0.869 | 0.816 |
| Q148 | 67 | What factors are associated with the utilization of maternal healthcare services in rural areas of Bangladesh? | Description | MNH | 0.967 | 0.901 | 1.038 | 0.700 | 0.806 | 0.868 | 0.822 |
| Q155 | 68 | What strategies can be implemented to improve fetal development during pregnancy? | Discovery | MNH | 0.940 | 0.939 | 1.037 | 0.701 | 0.793 | 0.868 | 0.760 |
| Q140 | 69 | What strategies can be implemented to ensure perinatal and postnatal mental health services to the women of Bangladesh? | Discovery | MNH | 0.945 | 0.885 | 1.061 | 0.705 | 0.809 | 0.867 | 0.782 |
| Q167 | 70 | What is the incidence and factors of Gestational diabetes mellitus (GDM) among the working mothers? | Description | MNH | 0.963 | 0.880 | 1.036 | 0.726 | 0.798 | 0.866 | 0.793 |
| Q041 | 71 | What would be the sustainable scaling-up strategy to address the effects of iron-deficiency and /or anaemia on adolescent reproductive health in Bangladesh? | Delivery | AH | 0.911 | 0.943 | 1.031 | 0.714 | 0.804 | 0.866 | 0.796 |
| Q083 | 72 | What is the impact of postpartum family planning (PPFP) focused community awareness meeting among pregnant women and PPFP-focused training among service providers on PPFP services? | Description | F | 0.969 | 0.934 | 1.031 | 0.698 | 0.766 | 0.866 | 0.809 |
| Q065 | 73 | What strategies can be adapted to improve the quality of care regarding family planning across different tiers of health systems? | Discovery | F | 0.924 | 0.946 | 1.031 | 0.698 | 0.798 | 0.866 | 0.788 |
| Q126 | 74 | What is the status of knowledge, attitude, practice, determinants and common errors regarding Antepartum Haemorrhage/ Postpartum Haemorrhage care management among service providers in Bangladesh? | Description | MNH | 0.954 | 0.910 | 1.045 | 0.646 | 0.842 | 0.865 | 0.805 |
| Q067 | 75 | What would be the most cost-effective, affordable, and feasible package of interventions for infertility among general and working women? | Development | F | 0.940 | 0.950 | 1.058 | 0.678 | 0.769 | 0.865 | 0.780 |
| Q162 | 76 | What are the opportunities and challenges associated with improving postnatal support and infant care for adolescent mothers at the community level, with particular attention to preterm babies? | Delivery | MNH | 0.906 | 0.941 | 1.067 | 0.690 | 0.781 | 0.863 | 0.781 |

| | | | | | | | | | | | |
|---|---|---|---|---|---|---|---|---|---|---|---|
| Q016 | 77 | What are the most effective and culturally sensitive strategies for promoting mental health and preventing mental health disorders among adolescents, and how can these strategies be successfully implemented and accepted within diverse adolescent populations? | Development | AH | 0.919 | 0.875 | 1.067 | 0.714 | 0.810 | 0.863 | 0.781 |
| Q066 | 78 | What is the prevalence and factors associated with infertility and fertility desires among general and working women of reproductive age in Bangladesh? | Description | F | 0.960 | 0.926 | 1.044 | 0.674 | 0.781 | 0.863 | 0.784 |
| Q111 | 79 | What are the optimal tools, instruments, approaches, or measures for assessing the impact of interventions addressing menstrual health across different programmatic levels (e.g., local, national, global)? | Discovery | GI | 0.920 | 0.926 | 1.085 | 0.656 | 0.793 | 0.862 | 0.795 |
| Q015 | 80 | What is the knowledge, attitude and practices of service providers on adolescent mental health service? | Description | AH | 0.911 | 0.914 | 1.070 | 0.685 | 0.801 | 0.862 | 0.807 |
| Q104 | 81 | What are the potential strategies to scale-up human papillomavirus (HPV) vaccination in Bangladesh? | Discovery | GI | 0.904 | 0.939 | 1.058 | 0.661 | 0.816 | 0.861 | 0.799 |
| Q166 | 82 | What are potential roles of enteropathogenic and environmental enteric dysfunction indicators in hindering childhood growth? | Description | MNH | 0.960 | 0.887 | 1.044 | 0.676 | 0.806 | 0.861 | 0.763 |
| Q113 | 83 | How can information on menstruation be integrated into existing formal and non-formal educational curriculam, health services (e.g. contraceptive services, HPV vaccination, FGM support, psychosocial support), social norms, and gender equality interventions/programmes? | Delivery | GI | 0.940 | 0.860 | 1.103 | 0.678 | 0.791 | 0.860 | 0.774 |
| Q005 | 84 | How can social media promote awareness of sexual and reproductive health issues including appropriate care seeking among adolescents? | Development | AH | 0.915 | 0.941 | 1.026 | 0.705 | 0.781 | 0.860 | 0.795 |
| Q084 | 85 | What approaches can be taken to report family planning (FP)/ postpartum family planning (PPFP) performances in a unified national multisystem inflammatory syndrome (MIS)? How to measure the coverage and quality of PPFP services through the MIS for home- and facility-based deliveries? | Discovery | F | 0.925 | 0.943 | 1.046 | 0.687 | 0.760 | 0.858 | 0.776 |
| Q011 | 86 | What are the factors influencing the implementation of school health promotion tools to address sexual and reproductive health and rights (SRHR) in Bangladesh? | Description | AH | 0.906 | 0.950 | 1.038 | 0.702 | 0.762 | 0.858 | 0.807 |
| Q080 | 87 | What are the factors that influence women's access to sexual and reproductive health services? | Description | F | 0.965 | 0.922 | 1.036 | 0.654 | 0.780 | 0.857 | 0.779 |
| Q050 | 88 | What are the barriers to improving service and readiness of government-led Adolescent friendly health corners (AFHCs)? | Delivery | AH | 0.972 | 0.929 | 1.004 | 0.678 | 0.765 | 0.856 | 0.807 |
| Q101 | 89 | What are the prevalence and factors associated with the human papillomavirus (HPV) among reproductive-aged women in Bangladesh? | Description | GI | 0.959 | 0.892 | 1.031 | 0.689 | 0.776 | 0.856 | 0.796 |
| Q128 | 90 | What are the differences in physical and mental health outcomes between women who undergo normal vaginal delivery and those who undergo cesarean section? | Description | MNH | 0.946 | 0.943 | 0.992 | 0.691 | 0.773 | 0.855 | 0.797 |
| Q006 | 91 | What is the impact of problematic usage of digital technology and social media on mental and physical health and wellbeing of adolescents? | Description | AH | 0.937 | 0.962 | 1.018 | 0.661 | 0.769 | 0.855 | 0.744 |
| Q062 | 92 | How does contraception impact on breastfeeding women? | Description | F | 0.951 | 0.922 | 1.042 | 0.642 | 0.788 | 0.855 | 0.768 |
| Q161 | 93 | What is the prevalence and associated factors of birth defects among facility deliveries? | Description | MNH | 0.951 | 0.887 | 1.045 | 0.699 | 0.762 | 0.855 | 0.800 |
| Q109 | 94 | How do financial barriers impact the ability of girls, women who menstruate to manage their menstruation? | Delivery | GI | 0.926 | 0.885 | 1.048 | 0.705 | 0.772 | 0.853 | 0.792 |
| Q076 | 95 | Are there any association between primary infertility and thyroid disorders? | Development | F | 0.942 | 0.877 | 1.096 | 0.637 | 0.780 | 0.853 | 0.760 |

| | | | | | | | | | | | |
|---|---|---|---|---|---|---|---|---|---|---|---|
| Q040 | 96 | What is the prevalence of polycystic ovary syndrome (PCOS) and endometriosis among school-going adolescents and health seeking behaviors? | Description | AH | 0.940 | 0.851 | 1.023 | 0.694 | 0.818 | 0.851 | 0.755 |
| Q079 | 97 | What is the safety and quality of fertility care in Bangladesh? | Description | F | 0.940 | 0.882 | 1.064 | 0.614 | 0.820 | 0.850 | 0.780 |
| Q051 | 98 | What are the benefits and harms of routine antibiotic prophylaxis with medical abortion? | Discovery | F | 0.946 | 0.888 | 1.066 | 0.642 | 0.779 | 0.850 | 0.745 |
| Q028 | 99 | How can we design, implement, and evaluate strategies that effectively reduce the prevalence of early marriage while ensuring their acceptability within the target community? | Development | AH | 0.899 | 0.913 | 1.027 | 0.701 | 0.780 | 0.850 | 0.765 |
| Q056 | 100 | What are the factors associated with long-acting contraceptive and permanent method (LARC/PM) use among married women in Bangladesh? | Description | F | 0.950 | 0.914 | 1.035 | 0.687 | 0.733 | 0.850 | 0.781 |
| Q093 | 101 | How effective are multi-faceted strategies (such as peer-education, mass media, and community-based interventions) in improving knowledge, awareness, and access to care for reproductive cancers, particularly cervical and breast cancer? | Development | GI | 0.926 | 0.865 | 1.048 | 0.708 | 0.770 | 0.850 | 0.796 |
| Q089 | 102 | What is the relationship between Body Mass Index (BMI) and breast cancer among the women of Bangladesh? | Development | GI | 0.915 | 0.909 | 1.048 | 0.662 | 0.780 | 0.849 | 0.767 |
| Q057 | 103 | What strategies can be used to increase the use of long-acting contraceptive and permanent method (LARC/PM) to prevent unwanted pregnancy? | Discovery | F | 0.904 | 0.914 | 1.038 | 0.705 | 0.752 | 0.849 | 0.747 |
| Q096 | 104 | What is the availability and readiness of obstetric fistula care in govt health facilities? | Description | GI | 0.890 | 0.900 | 1.027 | 0.681 | 0.812 | 0.848 | 0.798 |
| Q184 | 105 | What is the level of knowledge, attitude and practices of Key Populations (KPs) in relation to SRH services and the availability of those services in Bangladesh? | Description | SRHKP | 0.920 | 0.914 | 1.023 | 0.665 | 0.787 | 0.848 | 0.781 |
| Q119 | 106 | What is the status of availability and readiness of health facilities for Post-Abortion Care in Bangladesh? | Description | MNH | 0.946 | 0.885 | 1.031 | 0.675 | 0.773 | 0.848 | 0.770 |
| Q107 | 107 | What indicators are optimal for assessing menstrual health over time (e.g. related to norms, education, health, rights, etc.)? | Discovery | GI | 0.922 | 0.897 | 1.027 | 0.676 | 0.786 | 0.848 | 0.784 |
| Q115 | 108 | What strategies can be implemented to improve the well-being of women with miscarriage or fetal loss experience? | Discovery | GI | 0.928 | 0.941 | 1.029 | 0.655 | 0.752 | 0.847 | 0.772 |
| Q154 | 109 | What strategies can be implemented to enhance existing interventions aimed at reducing the incidence of preterm delivery and intrauterine growth restriction (IUGR) in pregnant women? | Discovery | MNH | 0.909 | 0.882 | 1.064 | 0.674 | 0.772 | 0.846 | 0.753 |
| Q189 | 110 | What initiatives can be employed by government and NGO to bring sex worker's offspring out from their maternal profession and ensure rights to select their chosen profession? | Discovery | SRHKP | 0.905 | 0.885 | 1.017 | 0.685 | 0.805 | 0.845 | 0.778 |
| Q125 | 111 | What is the role of maternal-near-miss-audits in evaluating the quality of obstetric care? | Development | MNH | 0.915 | 0.887 | 1.058 | 0.647 | 0.788 | 0.845 | 0.710 |
| Q012 | 112 | What is the prevalence of risky sexual behaviors among adolescents and factors associated with it? | Description | AH | 0.901 | 0.899 | 1.008 | 0.693 | 0.786 | 0.844 | 0.762 |
| Q172 | 113 | Can the sexual harms associated with sexualized drug use in Key Populations (KPs) be mitigated by developing and refining intervention approaches? | Development | SRHKP | 0.913 | 0.877 | 1.004 | 0.718 | 0.774 | 0.843 | 0.754 |
| Q064 | 114 | What strategies can be implemented to reduce the inequality in the use of family planning methods? | Discovery | F | 0.937 | 0.902 | 1.018 | 0.670 | 0.752 | 0.842 | 0.765 |
| Q025 | 115 | What is the level of knowledge, attitude and practice of healthy sexual and reproductive health behaviors among adolescents in Bangladesh? | Description | AH | 0.930 | 0.882 | 0.986 | 0.687 | 0.793 | 0.842 | 0.751 |

| | | | | | | | | | | | |
|---|---|---|---|---|---|---|---|---|---|---|---|
| Q138 | 116 | What is the association between maternal dietary fatty acid intake during pregnancy and the early childhood development of their offspring? | Development | MNH | 0.880 | 0.899 | 1.045 | 0.672 | 0.777 | 0.841 | 0.749 |
| Q070 | 117 | What is the association between previous abortion and fertility among women of reproductive age in Bangladesh? | Development | F | 0.927 | 0.893 | 1.008 | 0.664 | 0.780 | 0.841 | 0.764 |
| Q003 | 118 | What are the determinants and barriers to accessing sexual and reproductive health (SRH) services for adolescents? | | AH | 0.959 | 0.943 | 1.022 | 0.642 | 0.705 | 0.840 | 0.774 |
| Q185 | 119 | What is level of the knowledge, attitude, practice and barriers regarding SRHR service and contraceptive use among Forcibly Displaced Myanmar Nationals (FDMN) refugee women? | Description | SRHKP | 0.936 | 0.878 | 1.028 | 0.683 | 0.744 | 0.840 | 0.773 |
| Q127 | 120 | What strategies should be implemented to enhance women's decision-making power, particularly for those facing challenges related to a lack of decision-making autonomy, with the aim of improving maternal health outcomes during pregnancy? | Discovery | MNH | 0.933 | 0.885 | 1.024 | 0.681 | 0.740 | 0.839 | 0.769 |
| Q175 | 121 | What are the most effective strategies for preventing unintended pregnancies and improving their service seeking behaviors among women with HIV? | Development | SRHKP | 0.905 | 0.880 | 1.004 | 0.700 | 0.773 | 0.839 | 0.795 |
| Q137 | 122 | What is the association between maternal pre-pregnancy body mass index (BMI) and the occurrence of hypertensive disorders in pregnancy? | Development | MNH | 0.962 | 0.887 | 0.984 | 0.659 | 0.768 | 0.838 | 0.765 |
| Q171 | 123 | What is the impact of introducing Apps for doctor appointments to get easy access to Sexual and Reproductive Health Rights (SRHR) services as well as human-centered design approaches to improve doctor-patient relationship for the female sex workers in Bangladesh? | Description | SRHKP | 0.893 | 0.891 | 1.012 | 0.705 | 0.756 | 0.838 | 0.762 |
| Q013 | 124 | What factors affect effective communication between parents and adolescents regarding sexual and reproductive health issues in Bangladesh? | Description | AH | 0.930 | 0.847 | 1.029 | 0.664 | 0.779 | 0.836 | 0.759 |
| Q060 | 125 | How to define and develop metrics for quality assessment in contraception and abortion care? | Development | F | 0.891 | 0.917 | 0.990 | 0.653 | 0.796 | 0.836 | 0.738 |
| Q047 | 126 | What is the most acceptable and effective strategy for proving context specific sexual and reproductive health and rights (SRHR) knowledge among adolescents? | Development | AH | 0.890 | 0.849 | 1.004 | 0.717 | 0.783 | 0.835 | 0.789 |
| Q187 | 127 | How does sexual and reproductive health affect Key Populations' (KP) mental health? | Description | SRHKP | 0.895 | 0.819 | 1.043 | 0.702 | 0.785 | 0.835 | 0.785 |
| Q023 | 128 | Is there any association between sexually transmitted infection (STI) and menstrual practices among adolescent girls? | Development | AH | 0.888 | 0.922 | 1.035 | 0.637 | 0.762 | 0.835 | 0.762 |
| Q164 | 129 | What are the impacts of effective Retinopathy of prematurity (ROP) prevention interventions/screening on the level of prematurity? | Description | MNH | 0.932 | 0.851 | 1.029 | 0.669 | 0.759 | 0.834 | 0.759 |
| Q022 | 130 | What is the level of knowledge, attitude and practice on menstrual hygiene among adolescent girls of Bangladesh? | Description | AH | 0.903 | 0.880 | 1.008 | 0.650 | 0.796 | 0.834 | 0.793 |
| Q130 | 131 | How to involve male family members to ensure facility-based delivery? | Development | MNH | 0.956 | 0.883 | 0.992 | 0.654 | 0.750 | 0.833 | 0.739 |
| Q045 | 132 | How to involve adolescents in adolescent-specific program designing and make sure their voices are heard? | Development | AH | 0.885 | 0.908 | 1.013 | 0.685 | 0.738 | 0.832 | 0.716 |
| Q036 | 133 | What is the prevalence of STI among adolescent girls in Bangladesh? | Description | AH | 0.859 | 0.864 | 1.042 | 0.681 | 0.777 | 0.831 | 0.751 |
| Q106 | 134 | What is the impact of menstrual hygiene practices on preventing pelvic inflammatory disease and endometriosis from the time of menarche? | Description | GI | 0.913 | 0.892 | 1.022 | 0.632 | 0.759 | 0.830 | 0.761 |

| | | | | | | | | | | | |
|---|---|---|---|---|---|---|---|---|---|---|---|
| Q039 | 135 | What are the key barriers for adolescents to access TB and TB/HIV diagnostic and treatment services in high- and low-income countries, and how can these be overcome? | Delivery | AH | 0.899 | 0.862 | 0.990 | 0.666 | 0.801 | 0.830 | 0.758 |
| Q102 | 136 | What is the perspective of women, their family members, and caregivers regarding the value and impact of HPV testing for cervical cancer screening? | Description | GI | 0.893 | 0.848 | 1.041 | 0.689 | 0.746 | 0.830 | 0.760 |
| Q053 | 137 | What are the gaps in existing abortion stigma measurement tools, including determining which tools work best in diverse settings? | Description | F | 0.903 | 0.863 | 1.014 | 0.642 | 0.793 | 0.830 | 0.730 |
| Q099 | 138 | What are the attitudes and experiences of healthcare providers towards gynecological cancer screening in Bangladesh? | Description | GI | 0.896 | 0.935 | 0.972 | 0.656 | 0.756 | 0.830 | 0.748 |
| Q046 | 139 | What policies and laws need to be revised, updated or developed to implement evidence based adolescent sexual and reproductive health (SRH) activities in Bangladesh? | Development | AH | 0.913 | 0.909 | 0.969 | 0.683 | 0.725 | 0.827 | 0.752 |
| Q181 | 140 | How can we scale up the implementation of alcohol use disorder (AUD) treatment programmes integrated into HIV treatment programmes in low- and middle-income countries? | Delivery | SRHKP | 0.890 | 0.885 | 1.013 | 0.656 | 0.749 | 0.825 | 0.710 |
| Q097 | 141 | What are the trend of incidence and prevalence of gynecological conditions among adolescents, and what factors are contributing to gynecological conditions among? | Description | GI | 0.942 | 0.882 | 0.979 | 0.627 | 0.763 | 0.825 | 0.754 |
| Q108 | 142 | Are girls, women, and others who menstruate able to access and afford their preferred menstrual product/materials and what is the quality of these products/materials? | Delivery | GI | 0.893 | 0.890 | 1.009 | 0.642 | 0.759 | 0.825 | 0.755 |
| Q043 | 143 | What types of communication strategies work best to change the adolescent’s health behaviors? | Development | AH | 0.890 | 0.897 | 0.961 | 0.676 | 0.766 | 0.825 | 0.718 |
| Q095 | 144 | What are the potential benefits and challenges of promoting non-hormonal contraceptive options for improving the lives of women in Bangladesh? | Delivery | GI | 0.934 | 0.862 | 1.013 | 0.642 | 0.738 | 0.824 | 0.714 |
| Q139 | 145 | How does intrauterine growth restriction (IUGR) effect on nutritional status among children and physical well-being among adult in later life? | Description | MNH | 0.909 | 0.882 | 0.984 | 0.676 | 0.727 | 0.822 | 0.725 |
| Q188 | 146 | What is the prevalence of sex worker's offspring going to their maternal profession? | Description | SRHKP | 0.911 | 0.859 | 0.962 | 0.669 | 0.765 | 0.820 | 0.741 |
| Q114 | 147 | What are the short-term and long-term impacts of miscarriage or fetal loss on maternal mental health status? | Description | GI | 0.882 | 0.913 | 1.035 | 0.670 | 0.665 | 0.820 | 0.756 |
| Q100 | 148 | What are the knowledge, attitude, and practice and challenges of healthcare providers regarding gynecological fistula care in Bangladesh? | | GI | 0.893 | 0.885 | 1.000 | 0.618 | 0.766 | 0.819 | 0.737 |
| Q129 | 149 | What factors contribute to women's preference for private healthcare facilities during child birth? | Description | MNH | 0.939 | 0.887 | 1.015 | 0.613 | 0.705 | 0.819 | 0.725 |
| Q190 | 150 | What strategies can be employed to effective service according to the needs of Forcibly Displaced Myanmar Nationals (FDMN) refugee women? | Discovery | SRHKP | 0.933 | 0.841 | 0.954 | 0.664 | 0.768 | 0.819 | 0.728 |
| Q049 | 151 | What innovations/innovative approaches can be adapted to address the interventions regarding adolescent’s health problems in low-cost manner and how could these be sustainable and scaled up? | Discovery | AH | 0.874 | 0.894 | 1.013 | 0.642 | 0.733 | 0.818 | 0.743 |
| Q191 | 152 | How can new approaches be used to investigate and remedy Key Populations’ (KPs') unrealized rights? | Discovery | SRHKP | 0.883 | 0.852 | 0.987 | 0.699 | 0.733 | 0.818 | 0.721 |
| Q034 | 153 | how can we design, implement, and assess strategies that are both acceptable and effective in reducing the incidence of early childbearing? | Discovery | AH | 0.915 | 0.848 | 0.987 | 0.656 | 0.742 | 0.816 | 0.704 |

| | | | | | | | | | | | |
|---|---|---|---|---|---|---|---|---|---|---|---|
| Q042 | 154 | Are there any relationships between adolescent behavioral disorders and screen time? | Development | AH | 0.882 | 0.895 | 0.976 | 0.607 | 0.788 | 0.816 | 0.707 |
| Q069 | 155 | What is the relationship between adverse mental health conditions and fertility among women of reproductive age? | Development | F | 0.911 | 0.869 | 0.976 | 0.650 | 0.731 | 0.814 | 0.736 |
| Q173 | 156 | What are the short-term and long-term impacts of the unnecessary use of sexual drug in Key Populations (KPs) of Bangladesh? | Description | SRHKP | 0.888 | 0.871 | 0.967 | 0.659 | 0.746 | 0.813 | 0.735 |
| Q014 | 157 | What methods or metrics can be employed to enhance the precision of assessing bias among adolescent sexual and reproductive health (SRH) service providers? | Development | AH | 0.888 | 0.829 | 1.004 | 0.698 | 0.702 | 0.811 | 0.697 |
| Q054 | 158 | How can manual vacuum aspiration and misoprostol impact to eliminate sharp curettage in low-resource settings? | Description | F | 0.891 | 0.855 | 0.981 | 0.631 | 0.760 | 0.810 | 0.682 |
| Q061 | 159 | What is the safety, efficacy, side effects and non-contraceptive benefit profile of hormonal as well as oral contraception in specific adolescent populations and different age-group of women? | Discovery | F | 0.845 | 0.877 | 1.009 | 0.626 | 0.748 | 0.808 | 0.680 |
| Q159 | 160 | How can a smooth referral mechanism be established for the mothers who live in hard-to-reach areas? | Delivery | MNH | 0.897 | 0.857 | 0.973 | 0.633 | 0.744 | 0.808 | 0.723 |
| Q133 | 161 | What is the prevalence of insomnia among pregnant women and what interventions can be adapted to reduce its prevalence? | | MNH | 0.936 | 0.829 | 0.988 | 0.612 | 0.738 | 0.808 | 0.719 |
| Q146 | 162 | What is the process for validating cause of death results using InSilicoVA or InterVA algorithm-based Bayesian models? | Development | MNH | 0.940 | 0.892 | 0.933 | 0.624 | 0.714 | 0.807 | 0.652 |
| Q073 | 163 | How do the fertility preferences and actual fertility practices vary between employed and unemployed women as well as between women working in government (public) and non-government (private) organisations and what are the challenges faced by them? | Delivery | F | 0.946 | 0.829 | 0.973 | 0.612 | 0.737 | 0.806 | 0.728 |
| Q118 | 164 | What are the prevalence and factors associated with the uterine prolapse and urinary incontinence among women in Bangladesh? | Description | GI | 0.938 | 0.845 | 0.976 | 0.605 | 0.723 | 0.804 | 0.735 |
| Q002 | 165 | What are the current practices of physicians and nurses in primary health care related to sexual and reproductive health and rights? | Description | AH | 0.915 | 0.823 | 0.951 | 0.637 | 0.759 | 0.804 | 0.725 |
| Q058 | 166 | What are the determinants of the discontinuation and switching of contraceptive methods? | Description | F | 0.909 | 0.882 | 0.936 | 0.605 | 0.743 | 0.802 | 0.712 |
| Q105 | 167 | What is the impact of HPV vaccination on HPV prevalence at the population level? | Description | GI | 0.869 | 0.890 | 0.947 | 0.607 | 0.759 | 0.801 | 0.700 |
| Q149 | 168 | How can multi-drug resistance organisms be a big challenge for neonatal survival? | Discovery | MNH | 0.913 | 0.840 | 0.940 | 0.620 | 0.756 | 0.801 | 0.729 |
| Q145 | 169 | What is the impact of NASG (Non pneumatic Anti Shock Garment) to reduce maternal mortality in hard to reach in selected area? | Description | MNH | 0.861 | 0.796 | 0.985 | 0.642 | 0.777 | 0.799 | 0.692 |
| Q001 | 170 | What are the risk-factors of violence against adolescents as well as violence conducted by the adolescents at various levels (individual, family, community)? | Description | AH | 0.877 | 0.853 | 0.930 | 0.614 | 0.781 | 0.798 | 0.716 |
| Q142 | 171 | What is the difference in physical and mental health outcomes between housewife and employed women in pregnancy? | Description | MNH | 0.896 | 0.864 | 0.891 | 0.656 | 0.723 | 0.793 | 0.699 |
| Q004 | 172 | Are private care health facilities ready to provide sexual and reproductive health (SRH) services to adolescents? | Description | AH | 0.899 | 0.869 | 0.933 | 0.563 | 0.759 | 0.792 | 0.689 |
| Q044 | 173 | How does exposure to environmental toxins and pollutions can impact long term physical outcomes of them? | Description | AH | 0.906 | 0.823 | 0.964 | 0.573 | 0.756 | 0.791 | 0.703 |
| Q035 | 174 | How is the impact of disability on reproductive health outcomes? | Description | AH | 0.820 | 0.836 | 0.951 | 0.656 | 0.752 | 0.790 | 0.668 |

| | | | | | | | | | | | |
|---|---|---|---|---|---|---|---|---|---|---|---|
| Q092 | 175 | What is the feasibility, acceptability, and effects of door-to-door portable visual inspection with acetic acid (VIA) test by the Family Welfare Assistants for increasing the uptake of cervical intraepithelial lesion screening tests among rural Bangladesh? | Description | GI | 0.908 | 0.829 | 0.911 | 0.666 | 0.690 | 0.788 | 0.755 |
| Q071 | 176 | What is the impact of diet quality on fertility? | Description | F | 0.890 | 0.869 | 0.947 | 0.613 | 0.680 | 0.787 | 0.700 |
| Q037 | 177 | What are the reproductive and sexual health problems of adolescent boys in Bangladesh? | Description | AH | 0.904 | 0.786 | 0.964 | 0.598 | 0.735 | 0.785 | 0.668 |
| Q075 | 178 | How do national interventions on fertility rates impacted differently across the country? | Description | F | 0.902 | 0.841 | 0.939 | 0.617 | 0.680 | 0.783 | 0.655 |
| Q153 | 179 | What is the impact of unplanned pregnancy on neonatal health outcomes? | Description | MNH | 0.865 | 0.846 | 0.964 | 0.616 | 0.685 | 0.782 | 0.689 |
| Q074 | 180 | What are the trends and Age-Period-Cohort Effects of fertility rate (actual fertility rate and desired fertility rate) in Bangladesh? | Description | F | 0.928 | 0.809 | 0.911 | 0.615 | 0.691 | 0.778 | 0.647 |
| Q160 | 181 | What are multi-omics biomarkers associated with the occurrence of stillbirth and preterm birth? | Development | MNH | 0.926 | 0.771 | 0.927 | 0.615 | 0.698 | 0.775 | 0.649 |
| Q052 | 182 | What is the relationship between Quality-of-life measures for unintended pregnancy (UIP) and abortion? | Development | F | 0.909 | 0.829 | 0.884 | 0.609 | 0.675 | 0.769 | 0.665 |
| Q112 | 183 | How can lessons learned from the delivery of menstrual health interventions be optimally documented and shared? | Delivery | GI | 0.879 | 0.779 | 0.977 | 0.579 | 0.654 | 0.761 | 0.652 |
| Q063 | 184 | How have organisational policies been practiced in protecting the right of fertility preferences and actual fertility practices of women working in government (public) and non-government (private) organisations? | Description | F | 0.869 | 0.796 | 0.926 | 0.573 | 0.680 | 0.757 | 0.636 |
| Q116 | 185 | What is the impact of antioxidant supplements on balancing anti-Müllerian hormone (AMH) levels in women aged 20-30 years? | Description | GI | 0.837 | 0.771 | 0.872 | 0.592 | 0.703 | 0.743 | 0.655 |
| Q144 | 186 | What is the impact of introducing an app-based fluid calculator on the prevention of fluid overload among post caesarean section pregnant women? | Description | MNH | 0.863 | 0.716 | 0.884 | 0.570 | 0.728 | 0.740 | 0.614 |
| Q068 | 187 | What are the impacts of staying abroad of non-resident Bangladeshi male on fertility state of their female partners? | Description | F | 0.911 | 0.754 | 0.855 | 0.586 | 0.649 | 0.739 | 0.636 |
| Q180 | 188 | Is transdiagnostic or common elements intervention approach possible for integrating alcohol use disorder (AUD) treatment into HIV clinics? | Delivery | SRHKP | 0.836 | 0.762 | 0.904 | 0.604 | 0.630 | 0.735 | 0.620 |
| Q038 | 189 | What are the vaginal microbiota status of Bangladeshi rural adolescent girls and its relation with the development of endometriosis? | Description | AH | 0.773 | 0.737 | 0.942 | 0.566 | 0.709 | 0.734 | 0.592 |
| Q030 | 190 | Can conditional cash transfer reduce early marriage among adolescent girls in rural Bangladesh? | Development | AH | 0.783 | 0.772 | 0.869 | 0.626 | 0.669 | 0.732 | 0.622 |
| Q178 | 191 | Can alcohol reduction programmes be effectively integrated into HIV prevention, treatment and care programmes? | Delivery | SRHKP | 0.783 | 0.757 | 0.884 | 0.602 | 0.666 | 0.726 | 0.595 |
| Q072 | 192 | What are the trends and patterns of proximate determinants of fertility and their contribution to overall proximate determinants of fertility indices? | Description | F | 0.816 | 0.695 | 0.896 | 0.562 | 0.691 | 0.720 | 0.577 |
| Q094 | 193 | What is the effectiveness of non-hormonal contraceptive options on women in Bangladesh after menopause? | Description | GI | 0.707 | 0.732 | 0.912 | 0.637 | 0.663 | 0.718 | 0.623 |
| Q177 | 194 | What is the link between alcohol use and adherence to HIV medication? | Development | SRHKP | 0.838 | 0.756 | 0.807 | 0.578 | 0.635 | 0.711 | 0.597 |
| Q087 | 195 | How does a SARS-CoV-2 infection affect a man's chance of developing damage to his testicles and his overall sexual and reproductive health? | Description | F | 0.787 | 0.707 | 0.850 | 0.490 | 0.635 | 0.683 | 0.526 |
| Q077 | 196 | What is the impact of gonadotrophins on ovulation induction among couples receiving fertility treatment, especially in cases where the husband is an expatriate? | Description | F | 0.754 | 0.753 | 0.809 | 0.507 | 0.637 | 0.681 | 0.527 |

| | | | | | | | | | | | |
|---|---|---|---|---|---|---|---|---|---|---|---|
| Q086 | 197 | What is the impact of pre-marital sexual activity, cohabitation and non-marital childbearing on overall fertility? | Description | F | 0.692 | 0.602 | 0.781 | 0.473 | 0.625 | 0.624 | 0.482 |

RPS - research priority score
iRPS – intermediate research priority score
AEA – average expert agreement
AH – adolescent health
F – fertility
GI – gynecological issues
MNH – maternal and neonatal health
SRHKP – sexual and reproductive health of key populations

**Table S3.** All the 197 Research questions with domain, theme, scores for each criterion, weighted RPS, and AEA based on Theme-wise expert input

| Question No. | Rank | Research Question | Domain | Theme | Answerability iRPS | Deliverability iRPS | Effectiveness iRPS | Equity iRPS | Maximum impact on burden iRPS | Weighted RPS | AEA (Excluding NWI) |
|---|---|---|---|---|---|---|---|---|---|---|---|
| Q078 | 1 | What strategies can be implemented to make infertility services more available, accessible, and affordable for marginalized populations? | Discovery | F | 1.014 | 1.106 | 1.205 | 0.802 | 0.907 | 0.991 | 0.978 |
| Q084 | 2 | What approaches can be taken to report family planning (FP)/ postpartum family planning (PPFP) performances in a unified national multisystem inflammatory syndrome (MIS)? How to measure the coverage and quality of PPFP services through the MIS for home- and facility-based deliveries? | Discovery | F | 1.014 | 1.106 | 1.205 | 0.849 | 0.850 | 0.989 | 0.975 |
| Q057 | 3 | What strategies can be used to increase the use of long-acting contraceptive and permanent method (LARC/PM) to prevent unwanted pregnancy? | Discovery | F | 0.968 | 1.106 | 1.205 | 0.849 | 0.866 | 0.983 | 0.963 |
| Q169 | 4 | What are the obstacles in raising awareness about the Sexual and Reproductive Health Rights (SRHR) of Key Populations (KPs) in Bangladesh? | Delivery | SRHKP | 1.014 | 1.052 | 1.147 | 0.836 | 0.892 | 0.972 | 0.948 |
| Q103 | 5 | What are the challenges, key factors, and enablers of implementing HPV testing for cervical cancer screening? | Delivery | GI | 1.014 | 1.051 | 1.205 | 0.807 | 0.861 | 0.972 | 0.940 |
| Q098 | 6 | What strategies can be implemented to scaling up the early detection of gynecological cancers in Bangladesh? | Discovery | GI | 1.014 | 1.044 | 1.205 | 0.807 | 0.861 | 0.971 | 0.939 |
| Q193 | 7 | How can stigma and discrimination against Key Populations be reduced to improve their sexual and reproductive health? | Discovery | SRHKP | 0.979 | 1.030 | 1.184 | 0.835 | 0.875 | 0.965 | 0.931 |
| Q082 | 8 | What is the role of gender in fertility preferences and practices, and how does it influence access to sexual and reproductive health services for women? | Description | F | 0.964 | 1.106 | 1.205 | 0.807 | 0.793 | 0.959 | 0.917 |
| Q101 | 9 | What are the prevalence and factors associated with the human papillomavirus (HPV) among reproductive-aged women in Bangladesh? | Description | GI | 1.014 | 0.995 | 1.145 | 0.807 | 0.861 | 0.949 | 0.900 |
| Q115 | 10 | What strategies can be implemented to improve the well-being of women with miscarriage or fetal loss experience? | Discovery | GI | 1.014 | 1.037 | 1.205 | 0.802 | 0.756 | 0.947 | 0.891 |
| Q195 | 11 | To what extent do key populations face socio-structural challenges that put their sexual and reproductive health right (SRHR) outcomes in jeopardy? | Description | SRHKP | 0.978 | 1.047 | 1.141 | 0.818 | 0.823 | 0.946 | 0.899 |
| Q110 | 12 | What effects do free or low-cost menstrual supplies have on the health of women who experience menstruation? | Description | GI | 1.014 | 0.983 | 1.138 | 0.807 | 0.861 | 0.945 | 0.896 |
| Q183 | 13 | What strategies need to be implemented to get better sexual and reproductive health & family planning service from government health system by key populations (KP)? | Discovery | SRHKP | 0.962 | 1.044 | 1.125 | 0.779 | 0.892 | 0.945 | 0.918 |
| Q192 | 14 | How do stigma and discriminatory practices impact the sexual and reproductive health behaviors of Key Populations? | Description | SRHKP | 0.981 | 1.014 | 1.105 | 0.807 | 0.892 | 0.944 | 0.913 |
| Q027 | 15 | How does the quality and accessibility of healthcare services for adolescents impact their knowledge and decision-making skills related to health? | Description | AH | 1.014 | 0.912 | 1.205 | 0.782 | 0.883 | 0.944 | 0.917 |

| | | | | | | | | | | | |
|---|---|---|---|---|---|---|---|---|---|---|---|
| Q197 | 16 | What is the current situation regarding the Sexual and Reproductive Health and Rights (SRHR) of Key Populations (KPs) in Bangladesh and the discrepancy between policy and the grounded reality? | Description | SRHKP | 0.979 | 1.027 | 1.122 | 0.791 | 0.875 | 0.944 | 0.910 |
| Q196 | 17 | What types of interventions and/or new technologies can be used to help key populations overcome the obstacles that threaten their sexual and reproductive health right (SRHR) outcomes? | Discovery | SRHKP | 0.996 | 1.062 | 1.119 | 0.774 | 0.842 | 0.943 | 0.891 |
| Q091 | 18 | What is the impact of a cancer screening registry (collects, utilizes, and stores cancer screening data on individuals) on program management and reporting? | Description | GI | 0.958 | 0.968 | 1.205 | 0.802 | 0.856 | 0.943 | 0.886 |
| Q170 | 19 | What challenges do Key Populations encounter in the diagnosis of COVID-19, treatment when COVID-19 positive, and the administration of vaccination against COVID-19? | Delivery | SRHKP | 0.996 | 1.000 | 1.141 | 0.818 | 0.826 | 0.941 | 0.898 |
| Q179 | 20 | What strategies and interventions can be adapted to improve access to and uptake of HIV testing services among Key Populations (KPs)? | Discovery | SRHKP | 0.996 | 1.011 | 1.122 | 0.774 | 0.874 | 0.940 | 0.894 |
| Q176 | 21 | What strategies or interventions can be employed to support pregnant adolescents living with HIV and to improve both maternal and child health outcomes? | Discovery | SRHKP | 0.996 | 1.027 | 1.119 | 0.774 | 0.842 | 0.936 | 0.893 |
| Q073 | 22 | How do the fertility preferences and actual fertility practices vary between employed and unemployed women as well as between women working in government (public) and non-government (private) organisations and what are the challenges faced by them? | Delivery | F | 1.014 | 0.995 | 1.205 | 0.722 | 0.816 | 0.935 | 0.900 |
| Q041 | 23 | What would be the sustainable scaling-up strategy to address the effects of iron-deficiency and /or anaemia on adolescent reproductive health in Bangladesh? | Delivery | AH | 0.989 | 0.989 | 1.145 | 0.764 | 0.861 | 0.935 | 0.879 |
| Q018 | 24 | What psychosocial interventions are available to support adolescent's wellbeing and mental health in Bangladesh? | Description | AH | 1.014 | 0.948 | 1.150 | 0.764 | 0.866 | 0.933 | 0.887 |
| Q064 | 25 | What strategies can be implemented to reduce the inequality in the use of family planning methods? | Discovery | F | 0.958 | 1.044 | 1.130 | 0.796 | 0.793 | 0.929 | 0.857 |
| Q186 | 26 | What strategy can be adapted to increase the access of socially neglected lesbian, gay, bisexual, transgender, queer or questioning (LGBTQ) individuals towards SRH services? | Discovery | SRHKP | 0.975 | 0.973 | 1.112 | 0.784 | 0.872 | 0.928 | 0.868 |
| Q055 | 27 | What key factors hinder or support birth spacing strategies for newlywed and underage married women, and how can they be integrated into healthcare systems? | Delivery | F | 1.014 | 0.995 | 1.085 | 0.849 | 0.771 | 0.928 | 0.900 |
| Q033 | 28 | How is adolescent pregnancy associated with child undernutrition, maternal anemia, and the risk of NCDs such as diabetes, and hypertension of the mother? | Description | AH | 1.014 | 0.960 | 1.085 | 0.743 | 0.907 | 0.927 | 0.879 |
| Q151 | 29 | What barriers hinder implementing Newborn Stabilizing Units in upazila hospitals? | Delivery | MNH | 0.993 | 0.976 | 1.125 | 0.776 | 0.828 | 0.924 | 0.879 |
| Q025 | 30 | What is the level of knowledge, attitude and practice of healthy sexual and reproductive health behaviors among adolescents in Bangladesh? | Description | AH | 0.992 | 0.955 | 1.142 | 0.753 | 0.848 | 0.923 | 0.862 |
| Q013 | 31 | What factors affect effective communication between parents and adolescents regarding sexual and reproductive health issues in Bangladesh? | Description | AH | 1.014 | 0.905 | 1.150 | 0.753 | 0.864 | 0.922 | 0.853 |
| Q028 | 32 | How can we design, implement, and evaluate strategies that effectively reduce the prevalence of early marriage while ensuring their acceptability within the target community? | Development | AH | 1.014 | 0.995 | 1.115 | 0.764 | 0.793 | 0.921 | 0.880 |

| | | | | | | | | | | | |
|---|---|---|---|---|---|---|---|---|---|---|---|
| Q067 | 33 | What would be the most cost-effective, affordable, and feasible package of interventions for infertility among general and working women? | Development | F | 0.958 | 1.044 | 1.071 | 0.755 | 0.850 | 0.921 | 0.841 |
| Q032 | 34 | What is the most acceptable and effective strategy for educating young adolescents about adverse effects of self-termination of pregnancy's using traditional medicine? | Development | AH | 0.961 | 0.931 | 1.142 | 0.782 | 0.859 | 0.920 | 0.874 |
| Q182 | 35 | How can structural interventions be utilised to promote a gender-inclusive sexual and reproductive health right (SRHR) intervention agenda for key populations (KPs)? | Development | SRHKP | 0.962 | 0.948 | 1.164 | 0.789 | 0.813 | 0.920 | 0.853 |
| Q049 | 36 | What innovations/innovative approaches can be adapted to address the interventions regarding adolescent's health problems in low-cost manner and how could these be sustainable and scaled up? | Discovery | AH | 0.991 | 1.005 | 1.150 | 0.695 | 0.824 | 0.918 | 0.864 |
| Q070 | 37 | What is the association between previous abortion and fertility among women of reproductive age in Bangladesh? | Development | F | 1.014 | 0.940 | 1.085 | 0.764 | 0.861 | 0.918 | 0.900 |
| Q194 | 38 | What forms of discrimination exist in providing sexual and reproductive health right (SRHR) services to Key Populations (KPs)? | Description | SRHKP | 0.962 | 0.991 | 1.122 | 0.728 | 0.860 | 0.918 | 0.882 |
| Q152 | 39 | What is the impact of the Postpartum Haemorrhage (PPH) bundle approach on the management of PPH cases? | Description | MNH | 0.981 | 0.995 | 1.087 | 0.746 | 0.849 | 0.917 | 0.878 |
| Q020 | 40 | What are the barriers faced by health care provider to provide mental health services among adolescents? | Delivery | AH | 0.951 | 0.955 | 1.130 | 0.772 | 0.850 | 0.917 | 0.845 |
| Q122 | 41 | Does the enhancement of counseling on danger signs during antenatal care (ANC) contribute to a significant reduction in adverse birth outcomes? | Description | MNH | 0.960 | 0.998 | 1.103 | 0.757 | 0.839 | 0.916 | 0.884 |
| Q141 | 42 | What is the burden and risk factors for mental health issues among pregnant women? | Description | MNH | 0.992 | 0.952 | 1.109 | 0.731 | 0.865 | 0.915 | 0.881 |
| Q090 | 43 | What is the cost-effectiveness of different gynecological cancer screening approaches in Bangladesh considering the local health system and socio-economic context? | Description | GI | 0.958 | 0.983 | 1.138 | 0.755 | 0.806 | 0.913 | 0.822 |
| Q132 | 44 | What are the bottlenecks, barriers, and challenges in identifying and managing high-risk pregnancies in low- and middle-income countries like Bangladesh? | Delivery | MNH | 0.981 | 0.958 | 1.113 | 0.734 | 0.848 | 0.912 | 0.850 |
| Q019 | 45 | What are the barriers to implementing adolescent-friendly mental health services at primary health care level? | Delivery | AH | 0.995 | 0.968 | 1.136 | 0.713 | 0.820 | 0.911 | 0.843 |
| Q113 | 46 | How can information on menstruation be integrated into existing formal and non-formal educational curriculam, health services (e.g. contraceptive services, HPV vaccination, FGM support, psychosocial support), social norms, and gender equality interventions/programmes? | Delivery | GI | 0.964 | 0.968 | 1.138 | 0.755 | 0.806 | 0.911 | 0.822 |
| Q017 | 47 | What is the prevalence and risk factors of different mental health conditions e.g., Common Mental Disorders (CMDs), Severe Mental Disorders (SMDs) among adolescents in Bangladesh? | Description | AH | 0.992 | 0.865 | 1.150 | 0.753 | 0.867 | 0.911 | 0.850 |
| Q124 | 48 | What is the status, impacts, and barriers of antenatal and postnatal care seeking of newborn both at facility and community? | Delivery | MNH | 0.979 | 0.974 | 1.107 | 0.743 | 0.824 | 0.911 | 0.852 |
| Q112 | 49 | How can lessons learned from the delivery of menstrual health interventions be optimally documented and shared? | Delivery | GI | 1.014 | 0.948 | 1.119 | 0.743 | 0.793 | 0.909 | 0.816 |
| Q174 | 50 | What extent do health care providers encounter difficulties or roadblocks when attending to Key | Description | SRHKP | 0.997 | 0.972 | 1.080 | 0.722 | 0.831 | 0.906 | 0.831 |

| | | | | | | | | | | |
|---|---|---|---|---|---|---|---|---|---|---|
| | | Populations (KPs) sexual and reproductive health (SRH) needs? | | | | | | | | |
| Q024 | 51 | What is the impact of health education on the preference of using menstrual kit among adolescent girl? | Description | AH | 0.991 | 0.948 | 1.150 | 0.748 | 0.756 | 0.904 | 0.832 |
| Q100 | 52 | What are the knowledge, attitude, and practice and challenges of healthcare providers regarding gynecological fistula care in Bangladesh? | | GI | 0.913 | 0.995 | 1.145 | 0.722 | 0.816 | 0.904 | 0.840 |
| Q031 | 53 | What is the impact of adolescent pregnancy on maternal mortality/morbidity? | Description | AH | 0.968 | 0.955 | 1.096 | 0.769 | 0.799 | 0.903 | 0.843 |
| Q163 | 54 | What are the most effective interventions for the prevention and treatment of low birth weight infants? | Description | MNH | 0.958 | 0.958 | 1.109 | 0.734 | 0.826 | 0.902 | 0.852 |
| Q008 | 55 | What is the impact of school-based comprehensive sexuality education given to young adolescents on prevention of gender-based violence, unsafe abortion, and smooth transitioning to adolescence from childhood? | Description | AH | 0.942 | 0.902 | 1.119 | 0.786 | 0.835 | 0.902 | 0.820 |
| Q015 | 56 | What is the knowledge, attitude and practices of service providers on adolescent mental health service? | Description | AH | 0.990 | 0.922 | 1.119 | 0.728 | 0.820 | 0.901 | 0.829 |
| Q034 | 57 | how can we design, implement, and assess strategies that are both acceptable and effective in reducing the incidence of early childbearing? | Discovery | AH | 0.991 | 0.922 | 1.074 | 0.714 | 0.861 | 0.898 | 0.815 |
| Q021 | 58 | What are the effects of unwanted pregnancy on peripartum depression among adolescent? | Description | AH | 0.948 | 0.930 | 1.054 | 0.739 | 0.888 | 0.897 | 0.819 |
| Q185 | 59 | What is level of the knowledge, attitude, practice and barriers regarding SRHR service and contraceptive use among Forcibly Displaced Myanmar Nationals (FDMN) refugee women? | Description | SRHKP | 0.979 | 0.968 | 1.076 | 0.774 | 0.761 | 0.897 | 0.865 |
| Q104 | 60 | What are the potential strategies to scale-up human papillomavirus (HPV) vaccination in Bangladesh? | Discovery | GI | 0.958 | 0.899 | 1.138 | 0.755 | 0.806 | 0.897 | 0.795 |
| Q009 | 61 | Does school-based comprehensive health and nutrition education program improve sexual and reproductive health, mental health, hygiene practice, and dietary diversity among adolescents? | Development | AH | 0.918 | 0.912 | 1.085 | 0.728 | 0.907 | 0.895 | 0.853 |
| Q147 | 62 | What are the current availability, quality, and accessibility of maternal and newborn health (MNH) services in hard-to-reach areas? | Description | MNH | 0.983 | 0.922 | 1.064 | 0.723 | 0.849 | 0.893 | 0.861 |
| Q026 | 63 | What is the sexual and reproductive health (SRH) knowledge status of adolescent boys and girls at district level in Bangladesh? | Description | AH | 0.990 | 0.940 | 1.062 | 0.708 | 0.839 | 0.893 | 0.816 |
| Q189 | 64 | What initiatives can be employed by government and NGO to bring sex worker's offspring out from their maternal profession and ensure rights to select their chosen profession? | Discovery | SRHKP | 0.979 | 0.934 | 1.054 | 0.728 | 0.826 | 0.890 | 0.845 |
| Q175 | 65 | What are the most effective strategies for preventing unintended pregnancies and improving their service seeking behaviors among women with HIV? | Development | SRHKP | 0.960 | 0.942 | 1.054 | 0.758 | 0.793 | 0.887 | 0.842 |
| Q184 | 66 | What is the level of knowledge, attitude and practices of Key Populations (KPs) in relation to SRH services and the availability of those services in Bangladesh? | Description | SRHKP | 0.949 | 0.958 | 1.069 | 0.726 | 0.804 | 0.887 | 0.838 |
| Q121 | 67 | How does the implementation of depression screening tools in antenatal and postnatal care affect the early detection of peripartum depression? | Description | MNH | 0.945 | 0.939 | 1.102 | 0.708 | 0.812 | 0.887 | 0.826 |
| Q150 | 68 | What is the impact of hypothermia prevention bundle care to prevent neonatal morbidity and mortality among facility delivered newborn? | Description | MNH | 0.968 | 0.964 | 1.068 | 0.681 | 0.822 | 0.886 | 0.806 |

| | | | | | | | | | | | |
|---|---|---|---|---|---|---|---|---|---|---|---|
| Q114 | 69 | What are the short-term and long-term impacts of miscarriage or fetal loss on maternal mental health status? | Description | GI | 1.014 | 0.968 | 1.071 | 0.796 | 0.655 | 0.886 | 0.791 |
| Q088 | 70 | What is the knowledge, attitude and practice of menstrual regulation among reproductive aged women in Bangladesh? | Description | F | 1.014 | 0.899 | 1.071 | 0.708 | 0.806 | 0.885 | 0.841 |
| Q168 | 71 | What strategy can be adapted to improve newborn interventions (ENC, KMC, Newborn Signal Functions etc.) for providing better support for premature and low birth weight newborns across all levels of healthcare facilities? | Discovery | MNH | 0.940 | 0.938 | 1.061 | 0.739 | 0.820 | 0.885 | 0.815 |
| Q059 | 72 | What are the most effective strategies to increase access to contraception in low resource settings like Bangladesh? | Development | F | 0.913 | 0.983 | 1.024 | 0.764 | 0.806 | 0.884 | 0.854 |
| Q043 | 73 | What types of communication strategies work best to change the adolescent’s health behaviors? | Development | AH | 0.964 | 0.940 | 1.047 | 0.722 | 0.816 | 0.883 | 0.778 |
| Q158 | 74 | What are the barriers to implement effective maternal health referral systems in hard-to-reach areas? | Delivery | MNH | 0.981 | 0.914 | 1.071 | 0.702 | 0.818 | 0.883 | 0.825 |
| Q010 | 75 | How can sexual and reproductive health (SRH) issues be incorporated in the national education curriculum? | Delivery | AH | 0.964 | 0.931 | 1.085 | 0.764 | 0.740 | 0.882 | 0.796 |
| Q188 | 76 | What is the prevalence of sex worker's offspring going to their maternal profession? | Description | SRHKP | 0.975 | 0.945 | 1.004 | 0.743 | 0.816 | 0.882 | 0.837 |
| Q157 | 77 | What is the burden and risk factors for pregnancy loss in Bangladesh? | Description | MNH | 0.958 | 0.909 | 1.085 | 0.685 | 0.846 | 0.882 | 0.790 |
| Q123 | 78 | What strategies can be implemented at community and facility level to increase at least four ANC with medically providers in low-resource settings? | Discovery | MNH | 0.967 | 0.948 | 1.051 | 0.691 | 0.822 | 0.882 | 0.813 |
| Q120 | 79 | What interventions can be adapted to improve maternal health counselling during periconceptional, ANC & PNC period? | Development | MNH | 0.928 | 0.962 | 1.100 | 0.698 | 0.791 | 0.882 | 0.797 |
| Q016 | 80 | What are the most effective and culturally sensitive strategies for promoting mental health and preventing mental health disorders among adolescents, and how can these strategies be successfully implemented and accepted within diverse adolescent populations? | Development | AH | 0.945 | 0.855 | 1.068 | 0.753 | 0.845 | 0.879 | 0.827 |
| Q109 | 81 | How do financial barriers impact the ability of girls, women who menstruate to manage their menstruation? | Delivery | GI | 0.845 | 0.968 | 1.138 | 0.708 | 0.806 | 0.879 | 0.750 |
| Q012 | 82 | What is the prevalence of risky sexual behaviors among adolescents and factors associated with it? | Description | AH | 0.899 | 0.922 | 1.068 | 0.772 | 0.804 | 0.879 | 0.789 |
| Q143 | 83 | What is the impact of implementing telehealth/mHealth technology by community health workers during home visits on neonatal survival? | Description | MNH | 0.979 | 0.922 | 1.051 | 0.721 | 0.791 | 0.878 | 0.790 |
| Q007 | 84 | What is the impact of introducing life skills lessons in the school educational curriculum on empowerment of adolescent girls and prevention of child marriage? | Description | AH | 0.989 | 0.857 | 1.054 | 0.722 | 0.839 | 0.878 | 0.810 |
| Q023 | 85 | Is there any association between sexually transmitted infection (STI) and menstrual practices among adolescent girls? | Development | AH | 0.930 | 0.938 | 1.100 | 0.672 | 0.812 | 0.876 | 0.780 |
| Q134 | 86 | What is the long-term health outcome of children who received Kangaroo Mother Care? | Description | MNH | 0.926 | 0.950 | 1.071 | 0.708 | 0.796 | 0.876 | 0.828 |
| Q135 | 87 | What is the level of knowledge, attitude, practice, challenges, and barriers of health care providers (Doctors, Nurse, Midwives) regarding establishing, practicing newborn interventions (Essential Newborn Care, Kangaroo Mother Care, Newborn Signal Functions etc.)? | Description | MNH | 0.982 | 0.906 | 1.061 | 0.692 | 0.806 | 0.875 | 0.823 |

| | | | | | | | | | | | |
|---|---|---|---|---|---|---|---|---|---|---|---|
| Q111 | 88 | What are the optimal tools, instruments, approaches, or measures for assessing the impact of interventions addressing menstrual health across different programmatic levels (e.g., local, national, global)? | Discovery | GI | 0.958 | 0.899 | 1.071 | 0.708 | 0.806 | 0.874 | 0.750 |
| Q003 | 89 | What are the determinants and barriers to accessing sexual and reproductive health (SRH) services for adolescents? | | AH | 0.990 | 0.960 | 1.024 | 0.693 | 0.764 | 0.872 | 0.806 |
| Q099 | 90 | What are the attitudes and experiences of healthcare providers towards gynecological cancer screening in Bangladesh? | Description | GI | 0.913 | 0.940 | 1.085 | 0.722 | 0.771 | 0.872 | 0.840 |
| Q063 | 91 | How have organisational policies been practiced in protecting the right of fertility preferences and actual fertility practices of women working in government (public) and non-government (private) organisations? | Description | F | 1.014 | 0.968 | 1.033 | 0.667 | 0.737 | 0.870 | 0.842 |
| Q029 | 92 | What is the effect of abuse (physical violence, sexual harassment and social bullies) on early marriage? | Description | AH | 0.845 | 0.902 | 1.047 | 0.743 | 0.861 | 0.866 | 0.808 |
| Q105 | 93 | What is the impact of HPV vaccination on HPV prevalence at the population level? | Description | GI | 0.942 | 0.829 | 1.119 | 0.728 | 0.777 | 0.865 | 0.735 |
| Q126 | 94 | What is the status of knowledge, attitude, practice, determinants and common errors regarding Antepartum Haemorrhage/ Postpartum Haemorrhage care management among service providers in Bangladesh? | Description | MNH | 0.959 | 0.905 | 1.044 | 0.637 | 0.846 | 0.864 | 0.810 |
| Q045 | 95 | How to involve adolescents in adolescent-specific program designing and make sure their voices are heard? | Development | AH | 0.942 | 0.885 | 1.115 | 0.701 | 0.748 | 0.864 | 0.762 |
| Q165 | 96 | How the health of newborn babies affected by their mothers' exposure to second-hand smoke during pregnancy? | Description | MNH | 0.958 | 0.926 | 1.031 | 0.676 | 0.796 | 0.863 | 0.802 |
| Q074 | 97 | What are the trends and Age-Period-Cohort Effects of fertility rate (actual fertility rate and desired fertility rate) in Bangladesh? | Description | F | 1.014 | 1.037 | 0.947 | 0.667 | 0.712 | 0.862 | 0.806 |
| Q172 | 98 | Can the sexual harms associated with sexualized drug use in Key Populations (KPs) be mitigated by developing and refining intervention approaches? | Development | SRHKP | 0.888 | 0.863 | 1.048 | 0.781 | 0.798 | 0.861 | 0.762 |
| Q093 | 99 | How effective are multi-faceted strategies (such as peer-education, mass media, and community-based interventions) in improving knowledge, awareness, and access to care for reproductive cancers, particularly cervical and breast cancer? | Development | GI | 0.913 | 0.940 | 1.024 | 0.722 | 0.771 | 0.860 | 0.820 |
| Q068 | 100 | What are the impacts of staying abroad of non-resident Bangladeshi male on fertility state of their female partners? | Description | F | 1.014 | 0.968 | 0.904 | 0.690 | 0.793 | 0.860 | 0.850 |
| Q140 | 101 | What strategies can be implemented to ensure perinatal and postnatal mental health services to the women of Bangladesh? | Discovery | MNH | 0.947 | 0.867 | 1.054 | 0.695 | 0.806 | 0.860 | 0.775 |
| Q094 | 102 | What is the effectiveness of non-hormonal contraceptive options on women in Bangladesh after menopause? | Description | GI | 0.824 | 0.948 | 1.054 | 0.796 | 0.737 | 0.858 | 0.718 |
| Q156 | 103 | What is the burden and causes of different types of high-risk pregnancy in Bangladesh? | Description | MNH | 0.942 | 0.917 | 1.033 | 0.647 | 0.820 | 0.858 | 0.809 |
| Q011 | 104 | What are the factors influencing the implementation of school health promotion tools to address sexual and reproductive health and rights (SRHR) in Bangladesh? | Description | AH | 0.918 | 0.940 | 1.033 | 0.688 | 0.777 | 0.857 | 0.769 |
| Q171 | 105 | What is the impact of introducing Apps for doctor appointments to get easy access to Sexual and Reproductive Health Rights (SRHR) services as well as human-centered design approaches to improve doctor-patient relationship for the female sex workers in Bangladesh? | Description | SRHKP | 0.913 | 0.858 | 1.044 | 0.736 | 0.801 | 0.857 | 0.772 |

| | | | | | | | | | | | |
|---|---|---|---|---|---|---|---|---|---|---|---|
| Q136 | 106 | What are the best possible way to improve the measurement of acute malnutrition in pregnant women? | Development | MNH | 0.958 | 0.905 | 1.058 | 0.676 | 0.756 | 0.856 | 0.776 |
| Q166 | 107 | What are potential roles of enteropathogenic and environmental enteric dysfunction indicators in hindering childhood growth? | Description | MNH | 0.966 | 0.873 | 1.039 | 0.664 | 0.805 | 0.856 | 0.759 |
| Q148 | 108 | What factors are associated with the utilization of maternal healthcare services in rural areas of Bangladesh? | Description | MNH | 0.963 | 0.880 | 1.021 | 0.685 | 0.796 | 0.855 | 0.804 |
| Q167 | 109 | What is the incidence and factors of Gestational diabetes mellitus (GDM) among the working mothers? | Description | MNH | 0.958 | 0.860 | 1.022 | 0.714 | 0.788 | 0.855 | 0.774 |
| Q102 | 110 | What is the perspective of women, their family members, and caregivers regarding the value and impact of HPV testing for cervical cancer screening? | Description | GI | 0.862 | 0.940 | 1.085 | 0.722 | 0.725 | 0.853 | 0.800 |
| Q131 | 111 | What are the short- and long-term maternal and child health consequences of c-section? | Discovery | MNH | 0.963 | 0.899 | 1.033 | 0.651 | 0.786 | 0.852 | 0.793 |
| Q161 | 112 | What is the prevalence and associated factors of birth defects among facility deliveries? | Description | MNH | 0.967 | 0.887 | 1.027 | 0.681 | 0.766 | 0.852 | 0.791 |
| Q002 | 113 | What are the current practices of physicians and nurses in primary health care related to sexual and reproductive health and rights? | Description | AH | 0.964 | 0.829 | 1.054 | 0.671 | 0.811 | 0.852 | 0.776 |
| Q155 | 114 | What strategies can be implemented to improve fetal development during pregnancy? | Discovery | MNH | 0.930 | 0.917 | 1.015 | 0.682 | 0.779 | 0.851 | 0.728 |
| Q048 | 115 | Is the micronutrient deficiency (Vitamin B12 and folic acid) among the adolescent pregnant women associated with adverse pregnancy outcomes? | Description | AH | 0.938 | 0.902 | 1.024 | 0.701 | 0.748 | 0.849 | 0.788 |
| Q128 | 116 | What are the differences in physical and mental health outcomes between women who undergo normal vaginal delivery and those who undergo cesarean section? | Description | MNH | 0.939 | 0.926 | 0.996 | 0.692 | 0.759 | 0.848 | 0.784 |
| Q050 | 117 | What are the barriers to improving service and readiness of government-led Adolescent friendly health corners (AFHCs)? | Delivery | AH | 0.991 | 0.869 | 0.986 | 0.656 | 0.804 | 0.847 | 0.798 |
| Q162 | 118 | What are the opportunities and challenges associated with improving postnatal support and infant care for adolescent mothers at the community level, with particular attention to preterm babies? | Delivery | MNH | 0.894 | 0.922 | 1.051 | 0.672 | 0.766 | 0.847 | 0.755 |
| Q187 | 119 | How does sexual and reproductive health affect Key Populations' (KP) mental health? | Description | SRHKP | 0.888 | 0.856 | 1.024 | 0.736 | 0.786 | 0.844 | 0.778 |
| Q005 | 120 | How can social media promote awareness of sexual and reproductive health issues including appropriate care seeking among adolescents? | Development | AH | 0.913 | 0.940 | 0.964 | 0.701 | 0.771 | 0.844 | 0.760 |
| Q058 | 121 | What are the determinants of the discontinuation and switching of contraceptive methods? | Description | F | 0.913 | 0.922 | 1.004 | 0.722 | 0.725 | 0.843 | 0.792 |
| Q051 | 122 | What are the benefits and harms of routine antibiotic prophylaxis with medical abortion? | Discovery | F | 0.951 | 0.899 | 1.054 | 0.584 | 0.793 | 0.843 | 0.700 |
| Q014 | 123 | What methods or metrics can be employed to enhance the precision of assessing bias among adolescent sexual and reproductive health (SRH) service providers? | Development | AH | 0.930 | 0.878 | 1.038 | 0.755 | 0.680 | 0.842 | 0.764 |
| Q125 | 124 | What is the role of maternal-near-miss-audits in evaluating the quality of obstetric care? | Development | MNH | 0.928 | 0.873 | 1.054 | 0.631 | 0.787 | 0.841 | 0.705 |
| Q039 | 125 | What are the key barriers for adolescents to access TB and TB/HIV diagnostic and treatment services in high- and low-income countries, and how can these be overcome? | Delivery | AH | 0.938 | 0.815 | 1.015 | 0.701 | 0.793 | 0.839 | 0.755 |
| Q119 | 126 | What is the status of availability and readiness of health facilities for Post-Abortion Care in Bangladesh? | Description | MNH | 0.939 | 0.872 | 1.026 | 0.656 | 0.769 | 0.839 | 0.758 |

| | | | | | | | | | | | |
|---|---|---|---|---|---|---|---|---|---|---|---|
| Q022 | 127 | What is the level of knowledge, attitude and practice on menstrual hygiene among adolescent girls of Bangladesh? | Description | AH | 0.904 | 0.804 | 1.048 | 0.676 | 0.828 | 0.838 | 0.779 |
| Q046 | 128 | What policies and laws need to be revised, updated or developed to implement evidence based adolescent sexual and reproductive health (SRH) activities in Bangladesh? | Development | AH | 0.938 | 0.912 | 1.024 | 0.658 | 0.725 | 0.838 | 0.720 |
| Q036 | 129 | What is the prevalence of STI among adolescent girls in Bangladesh? | Description | AH | 0.845 | 0.829 | 1.054 | 0.708 | 0.820 | 0.838 | 0.757 |
| Q006 | 130 | What is the impact of problematic usage of digital technology and social media on mental and physical health and wellbeing of adolescents? | Description | AH | 0.961 | 0.931 | 1.015 | 0.559 | 0.787 | 0.837 | 0.705 |
| Q065 | 131 | What strategies can be adapted to improve the quality of care regarding family planning across different tiers of health systems? | Discovery | F | 0.902 | 0.983 | 1.004 | 0.708 | 0.655 | 0.837 | 0.778 |
| Q117 | 132 | What is the impact of school or academic institution's support on adolescents in dealing with their gynecological concerns? | Description | GI | 0.862 | 0.899 | 0.964 | 0.755 | 0.771 | 0.836 | 0.809 |
| Q047 | 133 | What is the most acceptable and effective strategy for proving context specific sexual and reproductive health and rights (SRHR) knowledge among adolescents? | Development | AH | 0.964 | 0.815 | 0.994 | 0.701 | 0.771 | 0.835 | 0.798 |
| Q127 | 134 | What strategies should be implemented to enhance women's decision-making power, particularly for those facing challenges related to a lack of decision-making autonomy, with the aim of improving maternal health outcomes during pregnancy? | Discovery | MNH | 0.935 | 0.872 | 1.018 | 0.672 | 0.732 | 0.832 | 0.766 |
| Q138 | 135 | What is the association between maternal dietary fatty acid intake during pregnancy and the early childhood development of their offspring? | Development | MNH | 0.865 | 0.887 | 1.041 | 0.662 | 0.773 | 0.832 | 0.739 |
| Q095 | 136 | What are the potential benefits and challenges of promoting non-hormonal contraceptive options for improving the lives of women in Bangladesh? | Delivery | GI | 0.951 | 0.790 | 1.054 | 0.690 | 0.737 | 0.831 | 0.692 |
| Q191 | 137 | How can new approaches be used to investigate and remedy Key Populations' (KPs') unrealized rights? | Discovery | SRHKP | 0.867 | 0.885 | 0.984 | 0.740 | 0.741 | 0.830 | 0.737 |
| Q154 | 138 | What strategies can be implemented to enhance existing interventions aimed at reducing the incidence of preterm delivery and intrauterine growth restriction (IUGR) in pregnant women? | Discovery | MNH | 0.896 | 0.856 | 1.047 | 0.653 | 0.759 | 0.829 | 0.725 |
| Q190 | 139 | What strategies can be employed to effective service according to the needs of Forcibly Displaced Myanmar Nationals (FDMN) refugee women? | Discovery | SRHKP | 0.933 | 0.866 | 0.944 | 0.694 | 0.756 | 0.825 | 0.762 |
| Q080 | 140 | What are the factors that influence women's access to sexual and reproductive health services? | Description | F | 0.930 | 0.829 | 1.054 | 0.708 | 0.659 | 0.823 | 0.754 |
| Q079 | 141 | What is the safety and quality of fertility care in Bangladesh? | Description | F | 0.922 | 0.774 | 1.041 | 0.618 | 0.824 | 0.822 | 0.815 |
| Q066 | 142 | What is the prevalence and factors associated with infertility and fertility desires among general and working women of reproductive age in Bangladesh? | Description | F | 0.902 | 0.860 | 0.937 | 0.661 | 0.806 | 0.820 | 0.822 |
| Q137 | 143 | What is the association between maternal pre-pregnancy body mass index (BMI) and the occurrence of hypertensive disorders in pregnancy? | Development | MNH | 0.955 | 0.862 | 0.959 | 0.637 | 0.752 | 0.820 | 0.739 |
| Q159 | 144 | How can a smooth referral mechanism be established for the mothers who live in hard-to-reach areas? | Delivery | MNH | 0.937 | 0.860 | 0.982 | 0.632 | 0.752 | 0.819 | 0.735 |
| Q129 | 145 | What factors contribute to women's preference for private healthcare facilities during child birth? | Description | MNH | 0.942 | 0.887 | 1.021 | 0.607 | 0.703 | 0.819 | 0.717 |

| | | | | | | | | | | | |
|---|---|---|---|---|---|---|---|---|---|---|---|
| Q164 | 146 | What are the impacts of effective Retinopathy of prematurity (ROP) prevention interventions/screening on the level of prematurity? | Description | MNH | 0.921 | 0.829 | 1.021 | 0.643 | 0.738 | 0.817 | 0.736 |
| Q130 | 147 | How to involve male family members to ensure facility-based delivery? | Development | MNH | 0.948 | 0.865 | 0.964 | 0.637 | 0.729 | 0.816 | 0.714 |
| Q081 | 148 | What would be the most cost-effective, affordable, and feasible package of interventions for influencing women's access to sexual and reproductive health services? | Development | F | 0.913 | 0.940 | 0.964 | 0.722 | 0.605 | 0.815 | 0.735 |
| Q146 | 149 | What is the process for validating cause of death results using InSilicoVA or InterVA algorithm-based Bayesian models? | Development | MNH | 0.964 | 0.901 | 0.937 | 0.622 | 0.719 | 0.815 | 0.674 |
| Q139 | 150 | How does intrauterine growth restriction (IUGR) effect on nutritional status among children and physical well-being among adult in later life? | Description | MNH | 0.920 | 0.882 | 0.959 | 0.653 | 0.728 | 0.815 | 0.706 |
| Q056 | 151 | What are the factors associated with long-acting contraceptive and permanent method (LARC/PM) use among married women in Bangladesh? | Description | F | 0.913 | 0.860 | 1.024 | 0.680 | 0.635 | 0.809 | 0.776 |
| Q083 | 152 | What is the impact of postpartum family planning (PPFP) focused community awareness meeting among pregnant women and PPFP-focused training among service providers on PPFP services? | Description | F | 0.913 | 0.829 | 0.964 | 0.680 | 0.725 | 0.809 | 0.800 |
| Q060 | 153 | How to define and develop metrics for quality assessment in contraception and abortion care? | Development | F | 0.845 | 0.968 | 0.979 | 0.607 | 0.712 | 0.809 | 0.744 |
| Q054 | 154 | How can manual vacuum aspiration and misoprostol impact to eliminate sharp curettage in low-resource settings? | Description | F | 0.951 | 0.899 | 0.829 | 0.690 | 0.737 | 0.808 | 0.750 |
| Q042 | 155 | Are there any relationships between adolescent behavioral disorders and screen time? | Development | AH | 0.874 | 0.891 | 0.937 | 0.590 | 0.811 | 0.807 | 0.703 |
| Q076 | 156 | Are there any association between primary infertility and thyroid disorders? | Development | F | 0.902 | 0.799 | 1.071 | 0.613 | 0.705 | 0.805 | 0.667 |
| Q181 | 157 | How can we scale up the implementation of alcohol use disorder (AUD) treatment programmes integrated into HIV treatment programmes in low- and middle-income countries? | Delivery | SRHKP | 0.839 | 0.893 | 0.997 | 0.663 | 0.699 | 0.805 | 0.701 |
| Q173 | 158 | What are the short-term and long-term impacts of the unnecessary use of sexual drug in Key Populations (KPs) of Bangladesh? | Description | SRHKP | 0.888 | 0.819 | 0.937 | 0.698 | 0.739 | 0.803 | 0.730 |
| Q037 | 159 | What are the reproductive and sexual health problems of adolescent boys in Bangladesh? | Description | AH | 0.908 | 0.786 | 0.951 | 0.648 | 0.787 | 0.803 | 0.674 |
| Q061 | 160 | What is the safety, efficacy, side effects and non-contraceptive benefit profile of hormonal as well as oral contraception in specific adolescent populations and different age-group of women? | Discovery | F | 0.902 | 0.922 | 0.937 | 0.637 | 0.680 | 0.802 | 0.698 |
| Q085 | 161 | What are the impacts of measurement of the quality index indicators (good counselling, privacy and confidentiality during service provision, infection control and waste management) on maintaining quality of PPFP services? | Description | F | 0.958 | 0.860 | 0.937 | 0.661 | 0.655 | 0.801 | 0.689 |
| Q096 | 162 | What is the availability and readiness of obstetric fistula care in govt health facilities? | Description | GI | 0.789 | 0.760 | 1.071 | 0.708 | 0.737 | 0.800 | 0.767 |
| Q053 | 163 | What are the gaps in existing abortion stigma measurement tools, including determining which tools work best in diverse settings? | Description | F | 0.958 | 0.922 | 0.870 | 0.566 | 0.737 | 0.798 | 0.705 |
| Q001 | 164 | What are the risk-factors of violence against adolescents as well as violence conducted by the adolescents at various levels (individual, family, community)? | Description | AH | 0.845 | 0.857 | 0.964 | 0.607 | 0.777 | 0.797 | 0.689 |

| | | | | | | | | | | | |
|---|---|---|---|---|---|---|---|---|---|---|---|
| Q089 | 165 | What is the relationship between Body Mass Index (BMI) and breast cancer among the women of Bangladesh? | Development | GI | 0.830 | 0.774 | 1.041 | 0.680 | 0.725 | 0.797 | 0.615 |
| Q149 | 166 | How can multi-drug resistance organisms be a big challenge for neonatal survival? | Discovery | MNH | 0.902 | 0.811 | 0.937 | 0.613 | 0.759 | 0.792 | 0.712 |
| Q052 | 167 | What is the relationship between Quality-of-life measures for unintended pregnancy (UIP) and abortion? | Development | F | 0.913 | 0.774 | 0.964 | 0.680 | 0.680 | 0.789 | 0.720 |
| Q178 | 168 | Can alcohol reduction programmes be effectively integrated into HIV prevention, treatment and care programmes? | Delivery | SRHKP | 0.794 | 0.890 | 0.954 | 0.680 | 0.680 | 0.787 | 0.689 |
| Q118 | 169 | What are the prevalence and factors associated with the uterine prolapse and urinary incontinence among women in Bangladesh? | Description | GI | 0.862 | 0.829 | 0.964 | 0.661 | 0.680 | 0.786 | 0.681 |
| Q133 | 170 | What is the prevalence of insomnia among pregnant women and what interventions can be adapted to reduce its prevalence? | | MNH | 0.928 | 0.799 | 0.964 | 0.585 | 0.719 | 0.786 | 0.689 |
| Q040 | 171 | What is the prevalence of polycystic ovary syndrome (PCOS) and endometriosis among school-going adolescents and health seeking behaviors? | Description | AH | 0.865 | 0.691 | 1.004 | 0.625 | 0.800 | 0.784 | 0.647 |
| Q116 | 172 | What is the impact of antioxidant supplements on balancing anti-Müllerian hormone (AMH) levels in women aged 20-30 years? | Description | GI | 0.862 | 0.829 | 0.904 | 0.708 | 0.680 | 0.784 | 0.673 |
| Q145 | 173 | What is the impact of NASG (Non pneumatic Anti Shock Garment) to reduce maternal mortality in hard to reach in selected area? | Description | MNH | 0.864 | 0.806 | 0.947 | 0.607 | 0.756 | 0.783 | 0.663 |
| Q107 | 174 | What indicators are optimal for assessing menstrual health over time (e.g. related to norms, education, health, rights, etc.)? | Discovery | GI | 0.789 | 0.799 | 1.004 | 0.661 | 0.705 | 0.779 | 0.689 |
| Q097 | 175 | What are the trend of incidence and prevalence of gynecological conditions among adolescents, and what factors are contributing to gynecological conditions among? | Description | GI | 0.845 | 0.737 | 1.004 | 0.661 | 0.705 | 0.778 | 0.689 |
| Q106 | 176 | What is the impact of menstrual hygiene practices on preventing pelvic inflammatory disease and endometriosis from the time of menarche? | Description | GI | 0.845 | 0.829 | 1.004 | 0.613 | 0.655 | 0.777 | 0.659 |
| Q004 | 177 | Are private care health facilities ready to provide sexual and reproductive health (SRH) services to adolescents? | Description | AH | 0.908 | 0.846 | 0.886 | 0.543 | 0.756 | 0.775 | 0.640 |
| Q035 | 178 | How is the impact of disability on reproductive health outcomes? | Description | AH | 0.845 | 0.774 | 0.904 | 0.648 | 0.764 | 0.774 | 0.667 |
| Q142 | 179 | What is the difference in physical and mental health outcomes between housewife and employed women in pregnancy? | Description | MNH | 0.882 | 0.836 | 0.855 | 0.632 | 0.702 | 0.769 | 0.664 |
| Q062 | 180 | How does contraception impact on breastfeeding women? | Description | F | 0.845 | 0.799 | 0.937 | 0.613 | 0.705 | 0.767 | 0.689 |
| Q108 | 181 | Are girls, women, and others who menstruate able to access and afford their preferred menstrual product/materials and what is the quality of these products/materials? | Delivery | GI | 0.888 | 0.760 | 0.979 | 0.566 | 0.705 | 0.767 | 0.643 |
| Q160 | 182 | What are multi-omics biomarkers associated with the occurrence of stillbirth and preterm birth? | Development | MNH | 0.916 | 0.764 | 0.930 | 0.600 | 0.687 | 0.767 | 0.632 |
| Q153 | 183 | What is the impact of unplanned pregnancy on neonatal health outcomes? | Description | MNH | 0.849 | 0.817 | 0.937 | 0.591 | 0.660 | 0.759 | 0.655 |
| Q180 | 184 | Is transdiagnostic or common elements intervention approach possible for integrating alcohol use disorder (AUD) treatment into HIV clinics? | Delivery | SRHKP | 0.828 | 0.786 | 0.937 | 0.637 | 0.655 | 0.756 | 0.628 |

| | | | | | | | | | | | |
|---|---|---|---|---|---|---|---|---|---|---|---|
| Q044 | 185 | How does exposure to environmental toxins and pollutions can impact long term physical outcomes of them? | Description | AH | 0.930 | 0.728 | 0.937 | 0.495 | 0.716 | 0.749 | 0.641 |
| Q030 | 186 | Can conditional cash transfer reduce early marriage among adolescent girls in rural Bangladesh? | Development | AH | 0.797 | 0.768 | 0.888 | 0.690 | 0.655 | 0.747 | 0.641 |
| Q144 | 187 | What is the impact of introducing an app-based fluid calculator on the prevention of fluid overload among post caesarean section pregnant women? | Description | MNH | 0.869 | 0.723 | 0.874 | 0.578 | 0.752 | 0.747 | 0.631 |
| Q071 | 188 | What is the impact of diet quality on fertility? | Description | F | 0.824 | 0.691 | 0.904 | 0.637 | 0.680 | 0.735 | 0.625 |
| Q072 | 189 | What are the trends and patterns of proximate determinants of fertility and their contribution to overall proximate determinants of fertility indices? | Description | F | 0.845 | 0.676 | 0.937 | 0.613 | 0.605 | 0.723 | 0.644 |
| Q075 | 190 | How do national interventions on fertility rates impacted differently across the country? | Description | F | 0.888 | 0.760 | 0.904 | 0.546 | 0.518 | 0.712 | 0.605 |
| Q177 | 191 | What is the link between alcohol use and adherence to HIV medication? | Development | SRHKP | 0.832 | 0.796 | 0.747 | 0.595 | 0.598 | 0.702 | 0.552 |
| Q092 | 192 | What is the feasibility, acceptability, and effects of door-to-door portable visual inspection with acetic acid (VIA) test by the Family Welfare Assistants for increasing the uptake of cervical intraepithelial lesion screening tests among rural Bangladesh? | Description | GI | 0.812 | 0.737 | 0.783 | 0.680 | 0.544 | 0.700 | 0.694 |
| Q038 | 193 | What are the vaginal microbiota status of Bangladeshi rural adolescent girls and its relation with the development of endometriosis? | Description | AH | 0.716 | 0.651 | 0.942 | 0.566 | 0.680 | 0.699 | 0.512 |
| Q069 | 194 | What is the relationship between adverse mental health conditions and fertility among women of reproductive age? | Development | F | 0.652 | 0.711 | 0.829 | 0.584 | 0.648 | 0.674 | 0.595 |
| Q087 | 195 | How does a SARS-CoV-2 infection affect a man's chance of developing damage to his testicles and his overall sexual and reproductive health? | Description | F | 0.845 | 0.829 | 0.736 | 0.472 | 0.453 | 0.657 | 0.523 |
| Q077 | 196 | What is the impact of gonadotrophins on ovulation induction among couples receiving fertility treatment, especially in cases where the husband is an expatriate? | Description | F | 0.652 | 0.622 | 0.753 | 0.478 | 0.648 | 0.620 | 0.421 |
| Q086 | 197 | What is the impact of pre-marital sexual activity, cohabitation and non-marital childbearing on overall fertility? | Description | F | 0.676 | 0.492 | 0.536 | 0.378 | 0.403 | 0.489 | 0.400 |

RPS - research priority score
iRPS – intermediate research priority score
AEA – average expert agreement
AH – adolescent health
F – fertility
GI – gynecological issues
MNH – maternal and neonatal health
SRHKP – sexual and reproductive health of key populations

**Table S4.** Top 20 research questions with domain, theme, scores for each criterion, weighted RPS, and AEA based on Theme-wise expert input

| Question No. | Rank | Research Question | Domain | Theme | Answerability iRPS | Deliverability iRPS | Effectiveness iRPS | Equity iRPS | Maximum impact on burden iRPS | Weighted RPS | AEA (Excluding NWI) |
|---|---|---|---|---|---|---|---|---|---|---|---|
| Q078 | 1 | What strategies can be implemented to make infertility services more available, accessible, and affordable for marginalized populations? | Discovery | F | 1.014 | 1.106 | 1.205 | 0.802 | 0.907 | 0.991 | 0.978 |
| Q084 | 2 | What approaches can be taken to report family planning (FP)/ postpartum family planning (PPFP) performances in a unified national multisystem inflammatory syndrome (MIS)? How to measure the coverage and quality of PPFP services through the MIS for home- and facility-based deliveries? | Discovery | F | 1.014 | 1.106 | 1.205 | 0.849 | 0.850 | 0.989 | 0.975 |
| Q057 | 3 | What strategies can be used to increase the use of long-acting contraceptive and permanent method (LARC/PM) to prevent unwanted pregnancy? | Discovery | F | 0.968 | 1.106 | 1.205 | 0.849 | 0.866 | 0.983 | 0.963 |
| Q169 | 4 | What are the obstacles in raising awareness about the Sexual and Reproductive Health Rights (SRHR) of Key Populations (KPs) in Bangladesh? | Delivery | SRHKP | 1.014 | 1.052 | 1.147 | 0.836 | 0.892 | 0.972 | 0.948 |
| Q103 | 5 | What are the challenges, key factors, and enablers of implementing HPV testing for cervical cancer screening? | Delivery | GI | 1.014 | 1.051 | 1.205 | 0.807 | 0.861 | 0.972 | 0.940 |
| Q098 | 6 | What strategies can be implemented to scaling up the early detection of gynecological cancers in Bangladesh? | Discovery | GI | 1.014 | 1.044 | 1.205 | 0.807 | 0.861 | 0.971 | 0.939 |
| Q193 | 7 | How can stigma and discrimination against Key Populations be reduced to improve their sexual and reproductive health? | Discovery | SRHKP | 0.979 | 1.030 | 1.184 | 0.835 | 0.875 | 0.965 | 0.931 |
| Q082 | 8 | What is the role of gender in fertility preferences and practices, and how does it influence access to sexual and reproductive health services for women? | Description | F | 0.964 | 1.106 | 1.205 | 0.807 | 0.793 | 0.959 | 0.917 |
| Q101 | 9 | What are the prevalence and factors associated with the human papillomavirus (HPV) among reproductive-aged women in Bangladesh? | Description | GI | 1.014 | 0.995 | 1.145 | 0.807 | 0.861 | 0.949 | 0.900 |
| Q115 | 10 | What strategies can be implemented to improve the well-being of women with miscarriage or fetal loss experience? | Discovery | GI | 1.014 | 1.037 | 1.205 | 0.802 | 0.756 | 0.947 | 0.891 |
| Q195 | 11 | To what extent do key populations face socio-structural challenges that put their sexual and reproductive health right (SRHR) outcomes in jeopardy? | Description | SRHKP | 0.978 | 1.047 | 1.141 | 0.818 | 0.823 | 0.946 | 0.899 |
| Q110 | 12 | What effects do free or low-cost menstrual supplies have on the health of women who experience menstruation? | Description | GI | 1.014 | 0.983 | 1.138 | 0.807 | 0.861 | 0.945 | 0.896 |
| Q183 | 13 | What strategies need to be implemented to get better sexual and reproductive health & family planning service from government health system by key populations (KP)? | Discovery | SRHKP | 0.962 | 1.044 | 1.125 | 0.779 | 0.892 | 0.945 | 0.918 |
| Q192 | 14 | How do stigma and discriminatory practices impact the sexual and reproductive health behaviors of Key Populations? | Description | SRHKP | 0.981 | 1.014 | 1.105 | 0.807 | 0.892 | 0.944 | 0.913 |
| Q027 | 15 | How does the quality and accessibility of healthcare services for adolescents impact their knowledge and decision-making skills related to health? | Description | AH | 1.014 | 0.912 | 1.205 | 0.782 | 0.883 | 0.944 | 0.917 |
| Q197 | 16 | What is the current situation regarding the Sexual and Reproductive Health and Rights (SRHR) of Key Populations (KPs) in Bangladesh and the discrepancy between policy and the grounded reality? | Description | SRHKP | 0.979 | 1.027 | 1.122 | 0.791 | 0.875 | 0.944 | 0.910 |

| | | | | | | | | | | |
|---|---|---|---|---|---|---|---|---|---|---|
| Q196 | 17 | What types of interventions and/or new technologies can be used to help key populations overcome the obstacles that threaten their sexual and reproductive health right (SRHR) outcomes? | Discovery | SRHKP | 0.996 | 1.062 | 1.119 | 0.774 | 0.842 | 0.943 | 0.891 |
| Q091 | 18 | What is the impact of a cancer screening registry (collects, utilizes, and stores cancer screening data on individuals) on program management and reporting? | Description | GI | 0.958 | 0.968 | 1.205 | 0.802 | 0.856 | 0.943 | 0.886 |
| Q170 | 19 | What challenges do Key Populations encounter in the diagnosis of COVID-19, treatment when COVID-19 positive, and the administration of vaccination against COVID-19? | Delivery | SRHKP | 0.996 | 1.000 | 1.141 | 0.818 | 0.826 | 0.941 | 0.898 |
| Q179 | 20 | What strategies and interventions can be adapted to improve access to and uptake of HIV testing services among Key Populations (KPs)? | Discovery | SRHKP | 0.996 | 1.011 | 1.122 | 0.774 | 0.874 | 0.940 | 0.894 |

RPS - research priority score
iRPS – intermediate research priority score
AEA – average expert agreement
AH – adolescent health
F – fertility
GI – gynecological issues
MNH – maternal and neonatal health
SRHKP – sexual and reproductive health of key populations

**Table S5.** Top 10 research questions by AH theme based on AH experts input with domain, scores for each criterion, weighted RPS, and AEA

| Question No. | Overall Rank | Theme wise Rank | Research Question | Domain | Answerability iRPS | Deliverability iRPS | Effectiveness iRPS | Equity iRPS | Maximum impact on burden iRPS | Weighted RPS | AEA (Excluding NWI) |
|---|---|---|---|---|---|---|---|---|---|---|---|
| Q027 | 15 | 1 | How does the quality and accessibility of healthcare services for adolescents impact their knowledge and decision-making skills related to health? | Description | 1.014 | 0.912 | 1.205 | 0.782 | 0.883 | 0.944 | 0.917 |
| Q041 | 23 | 2 | What would be the sustainable scaling-up strategy to address the effects of iron-deficiency and /or anaemia on adolescent reproductive health in Bangladesh? | Delivery | 0.989 | 0.989 | 1.145 | 0.764 | 0.861 | 0.935 | 0.879 |
| Q018 | 24 | 3 | What psychosocial interventions are available to support adolescent's wellbeing and mental health in Bangladesh? | Description | 1.014 | 0.948 | 1.150 | 0.764 | 0.866 | 0.933 | 0.887 |
| Q033 | 28 | 4 | How is adolescent pregnancy associated with child undernutrition, maternal anemia, and the risk of NCDs such as diabetes, and hypertension of the mother? | Description | 1.014 | 0.960 | 1.085 | 0.743 | 0.907 | 0.927 | 0.879 |
| Q025 | 30 | 5 | What is the level of knowledge, attitude and practice of healthy sexual and reproductive health behaviors among adolescents in Bangladesh? | Description | 0.992 | 0.955 | 1.142 | 0.753 | 0.848 | 0.923 | 0.862 |
| Q013 | 31 | 6 | What factors affect effective communication between parents and adolescents regarding sexual and reproductive health issues in Bangladesh? | Description | 1.014 | 0.905 | 1.150 | 0.753 | 0.864 | 0.922 | 0.853 |
| Q028 | 32 | 7 | How can we design, implement, and evaluate strategies that effectively reduce the prevalence of early marriage while ensuring their acceptability within the target community? | Development | 1.014 | 0.995 | 1.115 | 0.764 | 0.793 | 0.921 | 0.880 |
| Q032 | 34 | 8 | What is the most acceptable and effective strategy for educating young adolescents about adverse effects of self-termination of pregnancy's using traditional medicine? | Development | 0.961 | 0.931 | 1.142 | 0.782 | 0.859 | 0.920 | 0.874 |
| Q049 | 36 | 9 | What innovations/innovative approaches can be adapted to address the interventions regarding adolescent's health problems in low-cost manner and how could these be sustainable and scaled up? | Discovery | 0.991 | 1.005 | 1.150 | 0.695 | 0.824 | 0.918 | 0.864 |
| Q020 | 40 | 10 | What are the barriers faced by health care provider to provide mental health services among adolescents? | Delivery | 0.951 | 0.955 | 1.130 | 0.772 | 0.850 | 0.917 | 0.845 |

RPS - research priority score

iRPS – intermediate research priority score

AEA – average expert agreement

AH – adolescent health

**Table S6.** Top 10 research questions by Fertility theme based on Fertility experts input with domain, scores for each criterion, weighted RPS, and AEA

| Question No. | Overall Rank | Theme wise Rank | Research Question | Domain | Answerability iRPS | Deliverability iRPS | Effectiveness iRPS | Equity iRPS | Maximum impact on burden iRPS | Weighted RPS | AEA (Excluding NWI) |
|---|---|---|---|---|---|---|---|---|---|---|---|
| Q078 | 1 | 1 | What strategies can be implemented to make infertility services more available, accessible, and affordable for marginalized populations? | Discovery | 1.014 | 1.106 | 1.205 | 0.802 | 0.907 | 0.991 | 0.978 |
| Q084 | 2 | 2 | What approaches can be taken to report family planning (FP)/ postpartum family planning (PPFP) performances in a unified national multisystem inflammatory syndrome (MIS)? How to measure the coverage and quality of PPFP services through the MIS for home- and facility-based deliveries? | Discovery | 1.014 | 1.106 | 1.205 | 0.849 | 0.850 | 0.989 | 0.975 |
| Q057 | 3 | 3 | What strategies can be used to increase the use of long-acting contraceptive and permanent method (LARC/PM) to prevent unwanted pregnancy? | Discovery | 0.968 | 1.106 | 1.205 | 0.849 | 0.866 | 0.983 | 0.963 |
| Q082 | 8 | 4 | What is the role of gender in fertility preferences and practices, and how does it influence access to sexual and reproductive health services for women? | Description | 0.964 | 1.106 | 1.205 | 0.807 | 0.793 | 0.959 | 0.917 |
| Q073 | 22 | 5 | How do the fertility preferences and actual fertility practices vary between employed and unemployed women as well as between women working in government (public) and non-government (private) organisations and what are the challenges faced by them? | Delivery | 1.014 | 0.995 | 1.205 | 0.722 | 0.816 | 0.935 | 0.900 |
| Q064 | 25 | 6 | What strategies can be implemented to reduce the inequality in the use of family planning methods? | Discovery | 0.958 | 1.044 | 1.130 | 0.796 | 0.793 | 0.929 | 0.857 |
| Q055 | 27 | 7 | What key factors hinder or support birth spacing strategies for newlywed and underage married women, and how can they be integrated into healthcare systems? | Delivery | 1.014 | 0.995 | 1.085 | 0.849 | 0.771 | 0.928 | 0.900 |
| Q067 | 33 | 8 | What would be the most cost-effective, affordable, and feasible package of interventions for infertility among general and working women? | Development | 0.958 | 1.044 | 1.071 | 0.755 | 0.850 | 0.921 | 0.841 |
| Q070 | 37 | 9 | What is the association between previous abortion and fertility among women of reproductive age in Bangladesh? | Development | 1.014 | 0.940 | 1.085 | 0.764 | 0.861 | 0.918 | 0.900 |
| Q088 | 70 | 10 | What is the knowledge, attitude and practice of menstrual regulation among reproductive aged women in Bangladesh? | Description | 1.014 | 0.899 | 1.071 | 0.708 | 0.806 | 0.885 | 0.841 |

RPS - research priority score
iRPS – intermediate research priority score
AEA – average expert agreement

**Table S7.** Top 10 research questions by GI theme based on GI experts input with domain, scores for each criterion, weighted RPS, and AEA

| Question No. | Overall Rank | Theme wise Rank | Research Question | Domain | Answerability iRPS | Deliverability iRPS | Effectiveness iRPS | Equity iRPS | Maximum impact on burden iRPS | Weighted RPS | AEA (Excluding NWI) |
|---|---|---|---|---|---|---|---|---|---|---|---|
| Q103 | 5 | 1 | What are the challenges, key factors, and enablers of implementing HPV testing for cervical cancer screening? | Delivery | 1.014 | 1.051 | 1.205 | 0.807 | 0.861 | 0.972 | 0.940 |
| Q098 | 6 | 2 | What strategies can be implemented to scaling up the early detection of gynecological cancers in Bangladesh? | Discovery | 1.014 | 1.044 | 1.205 | 0.807 | 0.861 | 0.971 | 0.939 |
| Q101 | 9 | 3 | What are the prevalence and factors associated with the human papillomavirus (HPV) among reproductive-aged women in Bangladesh? | Description | 1.014 | 0.995 | 1.145 | 0.807 | 0.861 | 0.949 | 0.900 |
| Q115 | 10 | 4 | What strategies can be implemented to improve the well-being of women with miscarriage or fetal loss experience? | Discovery | 1.014 | 1.037 | 1.205 | 0.802 | 0.756 | 0.947 | 0.891 |
| Q110 | 12 | 5 | What effects do free or low-cost menstrual supplies have on the health of women who experience menstruation? | Description | 1.014 | 0.983 | 1.138 | 0.807 | 0.861 | 0.945 | 0.896 |
| Q091 | 18 | 6 | What is the impact of a cancer screening registry (collects, utilizes, and stores cancer screening data on individuals) on program management and reporting? | Description | 0.958 | 0.968 | 1.205 | 0.802 | 0.856 | 0.943 | 0.886 |
| Q090 | 43 | 7 | What is the cost-effectiveness of different gynecological cancer screening approaches in Bangladesh considering the local health system and socio-economic context? | Description | 0.958 | 0.983 | 1.138 | 0.755 | 0.806 | 0.913 | 0.822 |
| Q113 | 46 | 8 | How can information on menstruation be integrated into existing formal and non-formal educational curriculam, health services (e.g. contraceptive services, HPV vaccination, FGM support, psychosocial support), social norms, and gender equality interventions/programmes? | Delivery | 0.964 | 0.968 | 1.138 | 0.755 | 0.806 | 0.911 | 0.822 |
| Q112 | 49 | 9 | How can lessons learned from the delivery of menstrual health interventions be optimally documented and shared? | Delivery | 1.014 | 0.948 | 1.119 | 0.743 | 0.793 | 0.909 | 0.816 |
| Q100 | 52 | 10 | What are the knowledge, attitude, and practice and challenges of healthcare providers regarding gynecological fistula care in Bangladesh? | | 0.913 | 0.995 | 1.145 | 0.722 | 0.816 | 0.904 | 0.840 |

RPS - research priority score

iRPS – intermediate research priority score

AEA – average expert agreement

GI – gynecological issues

**Table S8.** Top 10 research questions by MNH theme based on MNH experts input with domain, scores for each criterion, weighted RPS, and AEA

| Question No. | Overall Rank | Theme wise Rank | Research Question | Domain | Answerability iRPS | Deliverability iRPS | Effectiveness iRPS | Equity iRPS | Maximum impact on burden iRPS | Weighted RPS | AEA (Excluding NWI) |
|---|---|---|---|---|---|---|---|---|---|---|---|
| Q151 | 29 | 1 | What barriers hinder implementing Newborn Stabilizing Units in upazila hospitals? | Delivery | 0.993 | 0.976 | 1.125 | 0.776 | 0.828 | 0.924 | 0.879 |
| Q152 | 39 | 2 | What is the impact of the Postpartum Haemorrhage (PPH) bundle approach on the management of PPH cases? | Description | 0.981 | 0.995 | 1.087 | 0.746 | 0.849 | 0.917 | 0.878 |
| Q122 | 41 | 3 | Does the enhancement of counseling on danger signs during antenatal care (ANC) contribute to a significant reduction in adverse birth outcomes? | Description | 0.960 | 0.998 | 1.103 | 0.757 | 0.839 | 0.916 | 0.884 |
| Q141 | 42 | 4 | What is the burden and risk factors for mental health issues among pregnant women? | Description | 0.992 | 0.952 | 1.109 | 0.731 | 0.865 | 0.915 | 0.881 |
| Q132 | 44 | 5 | What are the bottlenecks, barriers, and challenges in identifying and managing high-risk pregnancies in low- and middle-income countries like Bangladesh? | Delivery | 0.981 | 0.958 | 1.113 | 0.734 | 0.848 | 0.912 | 0.850 |
| Q124 | 48 | 6 | What is the status, impacts, and barriers of antenatal and postnatal care seeking of newborn both at facility and community? | Delivery | 0.979 | 0.974 | 1.107 | 0.743 | 0.824 | 0.911 | 0.852 |
| Q163 | 54 | 7 | What are the most effective interventions for the prevention and treatment of low birth weight infants? | Description | 0.958 | 0.958 | 1.109 | 0.734 | 0.826 | 0.902 | 0.852 |
| Q147 | 62 | 8 | What are the current availability, quality, and accessibility of maternal and newborn health (MNH) services in hard-to-reach areas? | Description | 0.983 | 0.922 | 1.064 | 0.723 | 0.849 | 0.893 | 0.861 |
| Q121 | 67 | 9 | How does the implementation of depression screening tools in antenatal and postnatal care affect the early detection of peripartum depression? | Description | 0.945 | 0.939 | 1.102 | 0.708 | 0.812 | 0.887 | 0.826 |
| Q150 | 68 | 10 | What is the impact of hypothermia prevention bundle care to prevent neonatal morbidity and mortality among facility delivered newborn? | Description | 0.968 | 0.964 | 1.068 | 0.681 | 0.822 | 0.886 | 0.806 |

RPS - research priority score
iRPS – intermediate research priority score
AEA – average expert agreement
MNH – maternal and neonatal health

**Table S9.** Top 10 research questions by SRHKP theme based on SRHKP experts input with domain, scores for each criterion, weighted RPS, and AEA

| Question No. | Overall Rank | Theme wise Rank | Research Question | Domain | Answerability iRPS | Deliverability iRPS | Effectiveness iRPS | Equity iRPS | Maximum impact on burden iRPS | Weighted RPS | AEA (Excluding NWI) |
|---|---|---|---|---|---|---|---|---|---|---|---|
| Q169 | 4 | 1 | What are the obstacles in raising awareness about the Sexual and Reproductive Health Rights (SRHR) of Key Populations (KPs) in Bangladesh? | Delivery | 1.014 | 1.052 | 1.147 | 0.836 | 0.892 | 0.972 | 0.948 |
| Q193 | 7 | 2 | How can stigma and discrimination against Key Populations be reduced to improve their sexual and reproductive health? | Discovery | 0.979 | 1.030 | 1.184 | 0.835 | 0.875 | 0.965 | 0.931 |
| Q195 | 11 | 3 | To what extent do key populations face socio-structural challenges that put their sexual and reproductive health right (SRHR) outcomes in jeopardy? | Description | 0.978 | 1.047 | 1.141 | 0.818 | 0.823 | 0.946 | 0.899 |
| Q183 | 13 | 4 | What strategies need to be implemented to get better sexual and reproductive health & family planning service from government health system by key populations (KP)? | Discovery | 0.962 | 1.044 | 1.125 | 0.779 | 0.892 | 0.945 | 0.918 |
| Q192 | 14 | 5 | How do stigma and discriminatory practices impact the sexual and reproductive health behaviors of Key Populations? | Description | 0.981 | 1.014 | 1.105 | 0.807 | 0.892 | 0.944 | 0.913 |
| Q197 | 16 | 6 | What is the current situation regarding the Sexual and Reproductive Health and Rights (SRHR) of Key Populations (KPs) in Bangladesh and the discrepancy between policy and the grounded reality? | Description | 0.979 | 1.027 | 1.122 | 0.791 | 0.875 | 0.944 | 0.910 |
| Q196 | 17 | 7 | What types of interventions and/or new technologies can be used to help key populations overcome the obstacles that threaten their sexual and reproductive health right (SRHR) outcomes? | Discovery | 0.996 | 1.062 | 1.119 | 0.774 | 0.842 | 0.943 | 0.891 |
| Q170 | 19 | 8 | What challenges do Key Populations encounter in the diagnosis of COVID-19, treatment when COVID-19 positive, and the administration of vaccination against COVID-19? | Delivery | 0.996 | 1.000 | 1.141 | 0.818 | 0.826 | 0.941 | 0.898 |
| Q179 | 20 | 9 | What strategies and interventions can be adapted to improve access to and uptake of HIV testing services among Key Populations (KPs)? | Discovery | 0.996 | 1.011 | 1.122 | 0.774 | 0.874 | 0.940 | 0.894 |
| Q176 | 21 | 10 | What strategies or interventions can be employed to support pregnant adolescents living with HIV and to improve both maternal and child health outcomes? | Discovery | 0.996 | 1.027 | 1.119 | 0.774 | 0.842 | 0.936 | 0.893 |

RPS **-** research priority score

iRPS – intermediate research priority score

AEA – average expert agreement

SRHKP – sexual and reproductive health of key populations

**Table S10.** Cross-tabulation of 'area of expertise' and 'theme-wise participant count'

| | **Scored to Adolescent Health (AH)** | | |
|---|---|---|---|
| **Expertise in AH** | **No** | **Yes** | **Total** |
| **No** | 51 | 32 | 83 |
| **Yes** | 36 | 36 | 72 |
| **Total** | 87 | 68 | 155 |

| | **Scored to Fertility** | | |
|---|---|---|---|
| **Expertise in Fertility** | **No** | **Yes** | **Total** |
| **No** | 72 | 54 | 126 |
| **Yes** | 14 | 15 | 29 |
| **Total** | 86 | 69 | 155 |

| | **Scored to Gynecological Issues (GI)** | | |
|---|---|---|---|
| **Expertise in GI** | **No** | **Yes** | **Total** |
| **No** | 88 | 47 | 135 |
| **Yes** | 8 | 12 | 20 |
| **Total** | 96 | 59 | 155 |

| | **Scored to Maternal and Neonatal Health (MNH)** | | |
|---|---|---|---|
| **Expertise in MNH** | **No** | **Yes** | **Total** |
| **No** | 36 | 8 | 44 |
| **Yes** | 49 | 62 | 111 |
| **Total** | 85 | 70 | 155 |

| | **Scored to SRH of KP (SRHKP)** | | |
|---|---|---|---|
| **Expertise in SRHKP** | **No** | **Yes** | **Total** |
| **No** | 51 | 28 | 79 |
| **Yes** | 37 | 39 | 76 |
| **Total** | 88 | 67 | 155 |

| **Theme** | **Top 20 RQs based on total experts' input** | **Top 20 RQs based on theme-wise experts' input** | **Number of similar RQs in top 10 in both groups (total experts and theme-wise experts)** |
|---|---|---|---|
| **AH** | 6 | 1 | 5 |
| **F** | 1 | 4 | 4 |
| **GI** | 2 | 6 | 6 |
| **MNH** | 6 | 0 | 9 |
| **SRHKP** | 5 | 9 | 9 |

**Table S11.** Matrix mapping of the top 20 research questions to relevant SDG targets and indicators

| Rank | Research Question | Theme | SDG 3 | | | | | | SDG 4 | SDG 5 | | | |
|---|---|---|---|---|---|---|---|---|---|---|---|---|---|
| | | | 3.1 | 3.2 | 3.3 | 3.4 | 3.7 | 3.8 | 4.7 | 5.1 | 5.2 | 5.3 | 5.6 |
| 1 | How can stigma and discrimination against Key Populations be reduced to improve their sexual and reproductive health? | SRHKP | | | | | Stated | Stated | | Linked | Linked | | Linked |
| 2 | What barriers hinder implementing Newborn Stabilizing Units in upazila hospitals? | MNH | Stated | Stated | | | | Stated | | | | | |
| 3 | What are the obstacles in raising awareness about the Sexual and Reproductive Health Rights (SRHR) of Key Populations (KPs) in Bangladesh? | SRHKP | | | | | Stated | Stated | | Linked | | | Linked |
| 4 | What is the impact of the Postpartum Haemorrhage (PPH) bundle approach on the management of PPH cases? | MNH | Stated | | | | | Stated | | | | | |
| 5 | How is adolescent pregnancy associated with child undernutrition, maternal anemia, and the risk of NCDs such as diabetes, and hypertension of the mother? | AH | | Linked | | Linked | | | | | | | Stated |
| 6 | Does the enhancement of counselling on danger signs during antenatal care (ANC) contribute to a significant reduction in adverse birth outcomes? | MNH | Stated | | | | | Stated | | | | | Stated |
| 7 | What key factors hinder or support birth spacing strategies for newlywed and underage married women, and how can they be integrated into healthcare systems? | F | | | | | Linked | Linked | | | | | Linked |
| 8 | What is the burden and risk factors for mental health issues among pregnant women? | MNH | | | | Linked | | Stated | | | | | |
| 9 | What are the bottlenecks, barriers, and challenges in identifying and managing high-risk pregnancies in low- and middle-income countries like Bangladesh? | MNH | Stated | | | | | Stated | | | | | |
| 10 | What is the impact of school-based comprehensive sexuality education given to young adolescents on prevention of gender-based violence, unsafe abortion, and smooth transitioning to adolescence from childhood? | AH | | | | | | | Linked | Linked | Linked | | Stated |
| 11 | What are the barriers faced by health care provider to provide mental health services among adolescents? | AH | | | | Linked | | Linked | | | | | |
| 12 | What is the impact of adolescent pregnancy on maternal mortality/morbidity? | AH | Stated | | | | | | | | | | Stated |
| 13 | What are the challenges, key factors, and enablers of implementing HPV testing for cervical cancer screening? | GI | | | Stated | | Linked | Linked | | | | | Stated |
| 14 | What strategies need to be implemented to get better sexual and reproductive health & family planning service from government health system by key populations (KP)? | SRHKP | | | | | Stated | Stated | | | | | Linked |
| 15 | What are the most effective interventions for the prevention and treatment of low birth weight infants? | MNH | | Stated | | | | Stated | | | | | |
| 16 | What strategies can be implemented to scaling up the early detection of gynecological cancers in Bangladesh? | GI | | | Stated | | Linked | Linked | | | | | |
| 17 | What is the impact of introducing life skills lessons in the school educational curriculum on empowerment of adolescent girls and prevention of child marriage? | AH | | | | | | | Linked | | | Linked | Stated |
| 18 | What is the impact of health education on the preference of using menstrual kit among adolescent girl? | AH | | | | | Stated | | Linked | | | | Stated |
| 19 | How do stigma and discriminatory practices impact the sexual and reproductive health behaviors of Key Populations? | SRHKP | | | | | Stated | Stated | | Linked | Linked | | Linked |
| 20 | What challenges do key populations faced during COVID-19 testing, treatment when COVID-19 positive, and the administration of vaccination against COVID-19? | SRHKP | | | Stated | | | Stated | | Linked | | | Linked |

**Stated** means the area is mentioned in the target or indicator language.
**Linked** means the area relates to the SDG target but is not specifically mentioned in the target.
